# Evolution of Disease Response Genes in Loblolly Pine: Insights from Candidate Genes

**DOI:** 10.1371/journal.pone.0014234

**Published:** 2010-12-06

**Authors:** Elhan S. Ersoz, Mark H. Wright, Santiago C. González-Martínez, Charles H. Langley, David B. Neale

**Affiliations:** 1 Department of Plant Sciences, University of California Davis, Davis, California, United States of America; 2 Department of Biological Statistics and Computational Biology, Cornell University, Ithaca, New York, United States of America; 3 Department of Forest Systems and Resources, Center of Forest Research, Centro de Investigacion Forestal, Instituto Nacional de Investigación y Tecnología Agraria y Alimentaria-CIFOR-INIA, Madrid, Spain; 4 Department of Ecology and Evolution, University of California Davis, Davis, California, United States of America; 5 Institute of Forest Genetics, United States Department of Agriculture (USDA) Forest Service, Davis, California, United States of America; Montreal Botanical Garden, Canada

## Abstract

**Background:**

Host-pathogen interactions that may lead to a competitive co-evolution of virulence and resistance mechanisms present an attractive system to study molecular evolution because strong, recent (or even current) selective pressure is expected at many genomic loci. However, it is unclear whether these selective forces would act to preserve existing diversity, promote novel diversity, or reduce linked neutral diversity during rapid fixation of advantageous alleles. In plants, the lack of adaptive immunity places a larger burden on genetic diversity to ensure survival of plant populations. This burden is even greater if the generation time of the plant is much longer than the generation time of the pathogen.

**Methodology/Principal Findings:**

Here, we present nucleotide polymorphism and substitution data for 41 candidate genes from the long-lived forest tree loblolly pine, selected primarily for their prospective influences on host-pathogen interactions. This dataset is analyzed together with 15 drought-tolerance and 13 wood-quality genes from previous studies. A wide range of neutrality tests were performed and tested against expectations from realistic demographic models.

**Conclusions/Significance:**

Collectively, our analyses found that *axr (auxin response factor)*, *caf1 (chromatin assembly factor)* and *gatabp1 (gata binding protein 1)* candidate genes carry patterns consistent with directional selection and *erd3 (early response to drought 3)* displays patterns suggestive of a selective sweep, both of which are consistent with the *arm-race* model of disease response evolution. Furthermore, we have identified patterns consistent with diversifying selection at *erf1-like (ethylene responsive factor 1)*, *ccoaoemt (caffeoyl-CoA-O-methyltransferase)*, *cyp450-like (cytochrome p450-like)* and *pr4.3 (pathogen response 4.3)*, expected under the *trench-warfare* evolution model. Finally, a drought-tolerance candidate related to the plant cell wall, *lp5*, displayed patterns consistent with balancing selection. In conclusion, both arms-race and trench-warfare models seem compatible with patterns of polymorphism found in different disease-response candidate genes, indicating a mixed strategy of disease tolerance evolution for loblolly pine, a major tree crop in southeastern United States.

## Introduction

Host-pathogen interactions have long been recognized to have a major influence over levels of biological diversity and evolutionary trends [1]. Genetic variation for resistance to pests and pathogens is commonly observed in plant populations, yet the ecological and evolutionary forces modulating this variation are often unclear [2]. Several case studies investigated whether the genetic components of pathogen resistance are polygenic [3], [4] or based on single-genes with major effects [5], [6], with evidence found in support of both models. Currently, there are two major hypotheses used to describe plant-pathogen interactions. The *trench-warfare* hypothesis proposes that advance-retreat cycles of disease epidemics maintain relatively stable forms of resistance and susceptibility alleles over long periods of time. Alternatively, the *arms-race* hypothesis proposes that both the host and pathogen continually develop new resistance and virulence alleles, with rapid and successive fixation of these alleles resulting in a competitive co-evolution of host and pathogen [7].

Cases that support both hypotheses have been reported in several angiosperm species at the candidate gene level (reviewed in [7]). For instance, a series of studies on *R*-genes in *Arabidopsis thaliana* (*Rpm1*, *Rps2* and *Rps5*) [8], [9] provided evidence in favor of the *trench-warfare* hypothesis by observing patterns of nucleotide diversity and divergence consistent with balancing selection at these known resistance loci [10], [11]. Using the same methodology, Rose et al. [12] showed that the evolution of the *Pto* disease resistance mechanism of wild tomato species (*Lycopersicon*) against bacterial pathogen *Pseudomonas syringea* is influenced by a mixture of purifying and balancing selection. However, another study by Moeller & Tiffin [13] on maize showed evidence supporting the purifying selection and *arms-race* hypothesis. It is not clear whether the majority of plant-pathogen interactions can be exclusively described by either one of these hypotheses for all loci involved in a direct interaction. Collectively, the literature on population genetic analysis of evolutionary patterns at host resistance genes indicates that the dominant form of selection acting on defense genes differs among different components of defense mechanisms [7].

Previous studies on the molecular evolution of disease resistance in plants present a retrospective analysis on genes experimentally identified as loci directly interacting with a specific pathogen. Here, we attempt a prospective molecular study to identify plant-pathogen interaction loci in the forest tree loblolly pine, by exploiting the evolutionary implications of the *arms-race* and *trench-warfare* hypotheses. Unlike *Arabidopsis*, tomato and rice, loblolly pine forms large, out-crossing and randomly mating populations with little to no spatial genetic structure. Thus, analysis with population genetic methods in this system may be more appropriate than in other model systems since most population genetic models assume these characteristics and are sensitive to even slight violations.

We consider five distinct selective models, as described in Supplementary Material Table S1. Since the *arms-race* hypothesis proposes that plant and pathogen continually develop novel alleles which must be alternately countered in order to maintain survival of both plant and pathogen, we expect that this model of interaction would result in rapid fixation of novel alleles in each system and thus response loci in the plant would be characterized by successive selective sweeps, resulting in an accelerated rate of amino acid replacement substitutions. If a selective sweep has completed recently, we may be able to detect this from polymorphism data even if the locus has not experienced repeated sweeps over time, as might be expected for a relatively new interaction. Thus, we distinguish the recent selective sweep case from the directional selection (i.e. successive selective sweeps) case.

Alternatively, the *trench-warfare* model proposes that plant and pathogen populations co-evolve in an advance-retreat cycle, similar to a predator-prey relationship. In this case, we would expect that two or more alleles at response loci are present in the plant population, but the fitness of each allele would depend on the frequency of corresponding alleles in the pathogen population. Thus, selection either maintains existing alleles over long periods of time (balancing selection) or maintains diversity by selecting against fixation of existing alleles (diversifying selection). For balancing selection, we expect an excess of mid-frequency alleles and a deficit of fixed differences. In this context, shared polymorphisms with outgroup species may also exist if selection has been maintained from the ancestral species into both extent species. For diversifying selection, we expect an excess of mid-frequency alleles and an elevated level of diversity, but no shared polymorphisms. However, weak balancing selection may generate signals very similar to diversifying selection while shared polymorphisms with outgroup species are expected only under very strong balancing selection. Note that, it may not be trivial to distinguish between the signals generated by these two modes of selection.

To find support for these hypotheses, or exclude one or more of them, we performed three complementary population genetic analyses which utilize different aspects of our data. Methods based on the site frequency spectrum (SFS) provide information about the recent history of the population and deviations from neutral expectations can be interpreted as evidence in favor of one or more selective explanations. Selective pressures acting over longer periods of evolutionary history may be revealed by the patterns of nucleotide substitution relative to polymorphism levels when comparing to an outgroup species. When polymorphism data in a closely related outgroup species is also available, the presence of shared polymorphisms may be an indicator of shared selective pressures maintaining alleles in both species, in particular for species that diverged long ago. If several loci are available, the latter may be tested by fitting an isolation model (IM) of speciation which we performed here using Scots pine (*Pinus sylvestris*), a European pine that separated from loblolly pine at least 25 MYA, as outgroup. Additionally, in order to develop an accurate expectation for the site frequency spectrum (SFS) under neutrality, we considered recent fluctuations in population size and provided the most rigorous and complete demographical analysis yet for loblolly pine.

We note that our study is based on the assumption that disease response genes are likely under selection and, therefore, if a gene appears neutral it is most likely not involved in disease response or effects are not detectable with standard methods and sample sizes. The converse, of course, as may be the case with *lp5* (see Results), is not true: a gene bearing evidence of recent selection does not necessarily mean it is involved in a disease-response pathway or plant-pathogen interaction. Thus, our approach may serve to refine disease response candidate gene lists, but by itself does not identify either specific or generalized disease response loci. However, with the limited genomic and experimental resources available in forest trees and a very long-generation time limiting the pace of common garden and genetic experiments, population genetic studies such as this one represent a promising approach for narrowing the search for phenotypically important loci and the identification of genetic variation useful for improvement and management of commercially and ecologically important tree species such as loblolly pine.

## Methods

### Plant material and DNA isolation

Sequence data was generated from a sample of 32 haploid seed megagametophytes of loblolly pine (same plant material as in [14], see Table S2 from Supplementary Material). Note that these specimens are parents to a breeding population and are different than the set of samples used by Brown et al. [15], including several accessions from Florida not considered by [15]. For outgroup sequencing, two Scots pine (*Pinus sylvestris*) seed samples were provided by Dr. Päivi Komulainen and Dr. Outi Savolainen (University of Oulu, Finland). Scots pine was chosen as an outgroup since; *i*) its genetically well studied, with a genetic map and a mapping population, *ii*) has a comparative genetic map with loblolly pine, *iii*) Among the species that has comparative genetic maps with loblolly pine, it's the most distantly related species within the pine taxa. Total DNA was isolated using the QIAGEN Plant DNA extraction kit (Valencia, CA) in 96-well microtiter plate format, according to the manufacturer's protocol.

### Candidate gene selection

Forty-one candidate genes were sequenced specifically for this study. For the references associating the indicated candidates to disease response from the literature see Supplementary Material- References S1. The selection of candidate gene loci was based on three major criteria:

(1) *Positional* candidates: Genes mapping near QTLs for cell wall chemistry [16]. These are the genes that had prior evidence from studies on other species to be involved in disease resistance response and located near QTLs for cell wall chemistry components such as lignin content, and cellulose content. (2) *Expression* candidates: Genes identified to have differential expression in northern blot or membrane array experiments [17]–[19] conducted to identify loci that are differentially expressed in loblolly pine seedlings infected by the two studied pathogens and uninfected controls or through electronic northerns between EST libraries generated from tissues under several different stress treatments and (3) *Functional* candidates: Genes that have been selected from the literature on model species whose homologs were identified in loblolly pine through homology searches within EST libraries available for loblolly pine and can be genetically mapped to a single locus in loblolly pine. Note that in this case the extent of the candidate gene list is limited to the coverage of the loblolly pine EST libraries. Initially, loci were selected from two main functional classes: genes annotated as metabolic enzymes and genes annotated as regulators of gene expression. However, upon data mining from public sequence databases, a small number of promoter-like intergenic sequences were identified as putative targets for some of the transcription factor families that were previously identified as candidate genes. These were also included in the target locus list (Table S3 from Supplementary Material).

### PCR, sequencing and alignment

Conifer seeds' endosperm tissue, called megagametophyte is haploid. All the PCR and sequencing analyses was performed on DNA extracted from haploid megagametophyte tissue from the seeds. PCR and sequencing primers were designed and sequencing was performed as described in Gill et al. [20]. For PCR primer sequences see Table S4 from Supplementary Material. Sequencing reactions were run on ABI3730 sequencers at College of Agricultural Sciences, Genomics Facility (CGF) of UC-Davis. Consensus sequence from multiple reads of individual samples were generated from the trace files through base calling and multiple sequence alignment (MSA) by *phred* [21], *phrap* [22] and MACE*-multiple alignment consed extensions* (Gilliland & Langley, University of California, Davis), and visualized by *consed* [23]. All trace files were visually scanned for overlapping peaks as indicators of amplification from multiple loci (i.e. paralogs). Since the DNA used for amplification was haploid, any overlapping peaks in any sequence traces indicated amplification from multiple loci. Only contigs that consistently yielded sequences with higher than 40 *phred* quality score at all sites (according to the MACE alignment) were subjected to SNP calling. Furthermore, once a locus was identified to be putatively single locus, in cases when it was polymorphic between the parents of mapping populations, it was considered for genetic mapping in loblolly pine mapping populations [24] and checked for co-linearity and synteny between the loblolly pine and Scots pine comparative maps.

All loci were annotated by comparing MSAs with their respective template EST sequences, followed by manual editing according to the EST template, UniGene translations and known intron splicing sites. Sequences and alignments were submitted to NCBI-GenBank database with accession numbers EU392746-EU394132.

### Exclusion of paralogous regions

In studies that heavily utilize PCR amplification for studying patterns of diversity and divergence, one serious weakness is the high incidence of amplification of high sequence similarity paralogous loci that may complicate the observations. As described in the previous section, we have tested the polymorphisms from each of the candidate genes, for their segregation between parents of two loblolly pine mapping populations, and one pair of parents from Scots pine mapping population, as well as a pair of mapping population parents for each of Monterey pine (*Pinus radiata*) and slash pine (*Pinus elliottii*) since each of these species have a comparative map generated via ESTP markers originally designed for loblolly pine. We were hoping to genetically map all these loci in all available mapping population to test for synteny and orthology across the loci amplified across species, since in case of a paralog amplification problem, segregation patterns are not expected to be robust. Unfortunately, not many of the loci included as candidates are segregating in mapping populations. Across all 71 loci screened for patterns of selection in this study coming from wood quality, drought tolerance and disease response projects, only 25 was successfully placed on the linkage map for loblolly pine. This low number is mostly due to SNPs not segregating between the parents, and assay design issues associated with designing genotyping assays for screening larger samples. As a result, 25 of the 71 loci mentioned in this study were genetically mapped to single genetic positions in mapping populations. We also have used FBRC-family based association population of loblolly pine (∼16 progeny per family on average) to check for deviations from Mendelian expectations for segregation of these and other loci. None of the loci displayed a significant deviation from expected Mendelian patterns in the FBRC-families for their segregation. (See Supplementary Material- Results S1 for data and details). Therefore, we concluded that it is unlikely that the patterns observed are due to amplification from paralogous copies of the genes either within or between species.

### Fitting neutral demographic models

The site frequency spectrum (SFS) at each locus is the vector (*x_1_, x_2_, … , x_n-1_*), where *x_i_* is the number of polymorphisms observed in a sample of size *n* with *i* derived alleles and *n-i* ancestral alleles. However, for evaluating demographic models we did not distinguish the ancestral and derived alleles, even for loci for which an outgroup sequence was available. Thus, unless otherwise stated, all SFS analyses used the *folded* SFS, where the spectrum has *n/2* frequency classes and *i≤n-i* indicates the minor allele frequency.

To estimate parameters for demographic models described below, we used coalescent simulations to draw random *ancestral recombination graphs* (ARGs) conditional on specific demographic parameter values and compute the likelihood of observing the experimental data given these ARGs using custom programs. Then, we optimized these parameters by searching for a configuration which produced the highest likelihood. To compute the likelihood given a particular ancestral recombination graph (ARG) and the scaled mutation rate Θ, the probability of observing an SFS is a product of Poisson densities
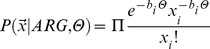
(1)where the Poisson rate *b_i_•*Θ is the sum of branch lengths in the ARG leading to a mutation of frequency *i*, times the scaled mutation rate Θ. As mentioned above, this likelihood formula depends on the demographic model and its parameters indirectly through the simulated ARG, so that all ARGs must be integrated over the space simulated to obtain the likelihood of the SFS given a particular demographic model. This integration is approximated by generating a large number of random ARGs conditional on the demographic model and averaging the likelihoods computed over this sample of ARGs. The likelihood can be computed to arbitrary precision by increasing the number of ARGs sampled. If *M* ARGs are sampled, the approximate likelihood formula for an individual locus is: 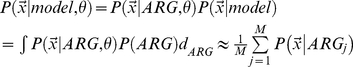
(2)


We assume all loci analyzed here are effectively unlinked with no interactions between loci (a reasonable assumption given the low levels of LD among candidate genes in this dataset), so the likelihood over all loci is simply the product of individual locus likelihoods.

The likelihood formula above is critically dependent on the scaled mutation rate Θ that is estimated from the data. Watterson's moment estimator Θ_W_ [25] is biased under non-equilibrium demography, but it is easily shown and verified with simulations that under simple demographic scenarios such as those considered here, this estimate is biased by a constant factor which does not depend itself on Θ. This constant factor can be estimated simply by simulating under the demographic model of interest with a known Θ and comparing this value with Θ_W_ estimated from the simulated data. For our evaluations here, we used this procedure to estimate the constant factor and correct Θ_W_ estimated from the data for each specific model considered. Since we expect considerable variability at each locus with respect to this parameter, we computed an average estimate across coding and putative promoter loci. This distinction is necessary since promoter loci harbor substantially more diversity than coding loci (see Table S5 from Supplementary Material).

Not shown in formulae [1] and [2] is the dependence of the ARGs on the scaled recombination rate. For generating ARGs with the coalescent, we used the average scaled recombination rate (*ρ* = 0.023/bp) [26] estimated from the data using LDHat [27] as a point estimate for this parameter. The likelihood calculation depends only weakly on the scaled recombination rate, so long as it is not zero or near zero, so results are expected to be robust to any reasonable uncertainty in this parameter.

**Figure 1:**
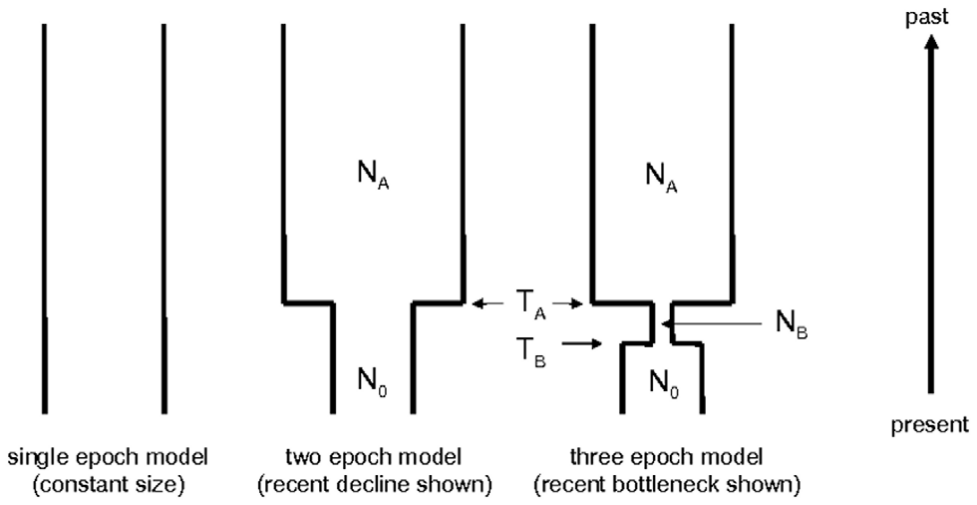
Bottleneck models tested. N_0 ,_ N_B_ and N_A_ refer to current, during bottleneck and before bottleneck effective population sizes, while T_A_ and T_B_ refer to time in 2N_0_ generations to the change from N_B_ to N_0_ and from N_A_ to N_B_ , respectively.

Previous work noted that glacial advances and retreats during recent ice ages restricted the habitable range of loblolly pine, causing fluctuations in *census* population size and most likely the *effective* population size [14], [15], [28], [29]. In order to generate a neutral expectation for the site frequency spectrum (SFS) at a locus, we must first determine if these suspected effective population size changes occurred and identify the time and magnitude of the change(s). We considered a simple model describing recent fluctuations in effective population size in two or three discrete epochs as well as the single epoch constant size (null) model (see Figure 1). Since previous studies have found little to no population structure across the extant range of this species [14], we did not investigate models incorporating structure or migration.

For the two-epoch and three-epoch demographic models, we searched the parameter space by evaluating the likelihood on a grid of points. To efficiently search this space and provide a finer density grid in regions with highest likelihoods, we first performed a search on a coarse grid of points over the widest sensible range of parameter values with *M = 10,000* samples, then selected a much smaller range of parameter values based on the regions with highest likelihood on the coarse grid. This smaller range is then searched again with finer spacing between grid points, using *M = 20,000* samples. Finally, since the highest likelihood models can differ only slightly in likelihood values and the evaluating equation [2] can result in slightly different values over multiple runs due to its stochastic nature, the highest likelihood models was evaluated again using *M = 1,000,000* samples and the average likelihood over 10 such evaluations was taken. Likewise, to increase stability of the grid searches, likelihoods were evaluated three times in both the coarse and fine grid searches, and the average of the three evaluations used to determine the range of the finer grid and the selection of models for the final deep sample evaluation.

The ranges searched for the coarse grid were T_b_ ∈ [0.01, 4.0], T_a_ ∈ [0.01, 4.0], N_b_/N_0_ ∈ [0.01, 100.0], N_a_/N_0_ ∈ [0.01, 100.0] (see Figure 1 for description of model parameters). For all parameters on the coarse grid, 15 points at regular intervals spanning these ranges on log-scale were chosen, resulting in 225 two-epoch models and 30,875 three-epoch models. In both the coarse and fine grid searches, three-epoch models specifying T_a_≤T_b_ were considered invalid and were not evaluated. The ranges for the fine grid search were T_b_ ∈ [0.01, 0.08], T_a_ ∈ [0.05, 0.5], N_b_/N_0_ ∈ [0.02, 0.4], N_a_/N_0_ ∈ [1.0, 10.0] for the three-epoch models and T_a_ ∈ [0.7, 1.8], N_a_/N_0_ ∈ [3.6, 30.0] for the two-epoch models. The finer search was conducted with 20 grid points at regular intervals on log-scale for each of the parameters, resulting in 273 two-epoch and 154,800 three-epoch models. Finally, based on the average likelihood of models evaluated in the fine grid search, all models within 4 log likelihood units of the maximum likelihood model were re-evaluated with a deeper sampling of *M = 1,000,000* ARGs, and the average of 10 evaluations taken as the final likelihood value. The top competing models within 2 log likelihood units of the maximum likelihood model in each class are presented as the final results, indicating both the maximum likelihood model and a range of “nearby” models which collectively describe the uncertainty in parameter estimates.

To test significance of the three different classes of demographic models, we note that the constant size model is a specific case of the two- and three-epoch models, and the two-epoch model is a specific case of the three-epoch model. Thus, with nested models, we can formally test the significance of higher likelihoods in the more complicated models with a standard likelihood ratio test. For the comparison of the two-epoch with the constant size model, and the three-epoch with the two-epoch model, twice the difference in log likelihood is expected to be χ^2^-distributed with two degrees of freedom, under the null hypothesis that the model with more parameters does not significantly describe the data better than the simpler model.

### Site Frequency Spectrum (SFS) diversity analysis

To assess whether individual loci were inconsistent with expectations under the best-fit neutral demographic model, coalescent samples of ARGs conditional on the best-fit model were generated placing mutations on the ARG conditional on the scaled mutation rate estimated for all loci within the coding and promoter locus classes, respectively. Then, the simulated site frequency spectra, corresponding summary statistics, and the likelihood of the simulated data were calculated. Empirical distributions of summary statistics, such as Tajima's D [30], Fu and Li's D [31] and the number of segregating sites, were compiled based on *M* coalescent simulations. Using these empirical distributions, the quantile (proportion) of the simulated values less than observed values can be calculated. Here, *M = 100,000* samples under the best-fit demographic model were used to generate the empirical distributions.

### McDonald-Kreitman Poisson Random Field (MKPRF) analysis

Information about average selective forces at a locus, over a longer period of evolutionary history, can be obtained from the level and patterns of nucleotide substitution between two closely related species. Under neutral evolution, polymorphisms represent a transient phase of the neutral substitution process, thus we should expect the ratio of substitutions between two species to polymorphisms within a species to be a function of the species divergence. Furthermore, if two distinct types of sites are both neutrally evolving, this ratio should be the same for each type. The standard McDonald-Krietman [32] test defines two types of sites within the same protein coding locus as silent or replacement based on whether or not the substitution or polymorphism alters the amino acid sequence. Under the assumption that silent sites are selectively neutral, a significant difference between the ratio of substitutions to polymorphisms for the two types of sites may indicate a locus is experiencing directional selection (too many replacement substitutions), or a strong selective constraint (too few replacement substitutions). This framework was generalized in the McDonald-Krietman Poisson Random Field (MKPRF) approach where information across multiple loci can be used to estimate global, locus-independent parameters such as the time of species divergence, allowing for more precise estimates of locus specific effects [33].

The MKPRF approach is based on a hierarchical Bayesian model which can describe not only the strength and direction of selection at individual loci but also an overall trend of selection across a group of loci. Briefly, the selection coefficient is defined for each replacement site *within* a locus, but modeled as independent, normally-distributed random variables with a common mean and variance. The program implementing the MKPRF approach provides a sample of the posterior distribution of the mean and variance of these random variables which is then summarized to describe the overall strength and direction of selection at each locus. In addition, these locus specific means and variances of selection coefficients are themselves treated as independent, normally-distributed random variables. These “hyper-parameters” describe the overall trend of selection *across* a group of loci indicating whether these loci as a group are experiencing positive or negative selection.

**Figure 2:**
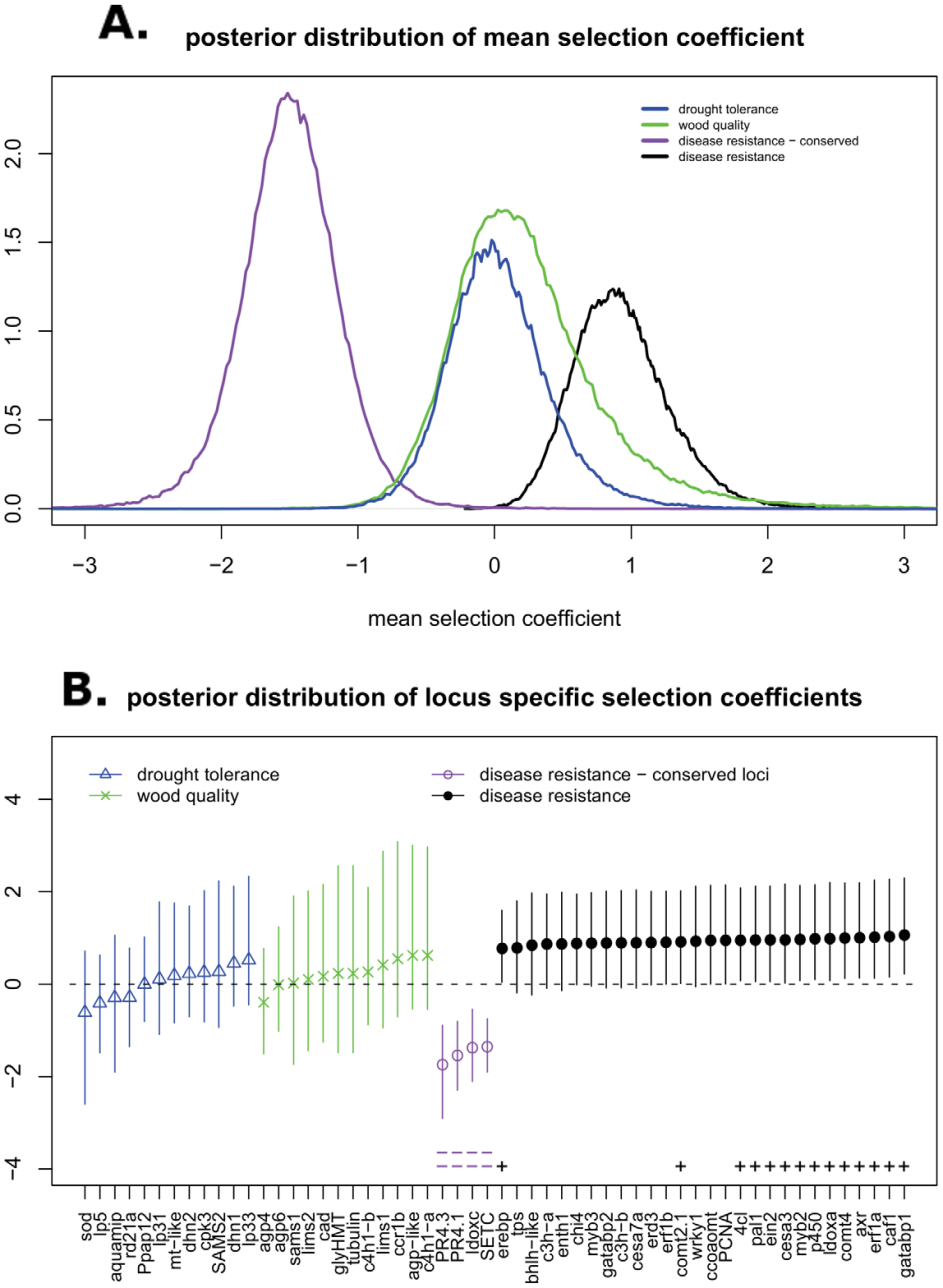
MKPRF analysis showing the posterior distribution of the mean selection coefficient across groups of loci (panel A), and the mean and 95% credible interval of the posterior distributions for per-locus selection coefficients (panel B). A selection coefficient less than zero implies selective constraint at amino acid replacement sites while a coefficient greater than zero implies accelerated fixation of amino acid replacements.

To perform the MKPRF analysis we used software provided by C. Bustamante, described in Bustamante et al. [33]. We distinguished the disease resistance loci sequenced specifically for this study from wood quality and drought tolerance candidate genes from previous studies [14], [15]. In addition, on the basis of preliminary results at the single locus level (see Figure 2), we subdivided the disease candidate loci into a conserved and non-conserved group (*pr4.3, pr4.1, ldox-c* and *set-like-c*; see Results and Supplementary Material Table S6). MKPRF was run with default parameters which specify diffuse, “uninformative” priors for the parameters of interest. MKPRF obtains samples from the posterior distribution of the parameters using the Monte Carlo Markov Chain (MCMC) approach. Ten independent chains were run for 10,000 “burn-in” iterations and then sampled every 10 iterations for a total of 10,000 samples for each chain. MKPRF outputs the Gelman-Rubin statistic for each parameter which in all cases was close to 1.0, indicating the chains have converged to the target posterior distribution. We summarized the posterior distributions of the selection coefficients for individual loci by the mean and 95% credible interval. The empirical densities obtained from 10,000 draws of the posterior distribution of the mean selection coefficient for groups of loci (see above) were directly plotted in Figure 2A. The input data can be found in Supplementary Material Table S6.

### Species Isolation Model (IM)

We used the method of Wakeley & Hey [34] as implemented in WH software to estimate the parameters of a general species isolation model with parameters *θ*_1_ = 4*N*_1_ *µ* of species 1, *θ*_2_ = 4*N*_2_ *µ* of species 2, *θ*_A_ = 4*N*_A_ *µ* of the ancestral population, and *τ = *2*µt*, the time of the divergence between the species in units of N_1_ generations. The IM model is based on counts of polymorphism specific to species 1 (S_x1_), polymorphism specific to species 2 (S_x2_), polymorphism fixed between the two species (S_F_) and polymorphism segregating in both species (S_S_). Once a general IM was fitted, we considered polymorphism types at each individual locus to assess whether observed patterns of polymorphism and divergence were consistent with expectations under this model. Similarly to the SFS analyses, coalescent simulations were performed to obtain the expected values under the fitted isolation model. Significance of deviations from these expectations for individual loci was assessed using the standard G-test (log-likelihood ratio test). Input data can be seen in Supplementary Material Table S7.

## Results

### Loblolly pine demography and SFS analysis

We explored the parameter space for the two- and three-epoch models in a maximum likelihood framework and formally compared each epoch class with a likelihood ratio test (see Methods). Parameters for the highest likelihood models in each class are given in Table 1. The likelihood ratio test between the maximum likelihood three-epoch and two-epoch models was highly significant (*p* = 4.52×10^−11^), showing that *at least* three periods of different population sizes must be considered to describe the data. This model describes a recent bottleneck in this species with about 85% reduction in effective population size, preceded by an ancestral epoch with an effective population size roughly four times the present epoch size. This is consistent with a population decline, possibly during a series of glacial advances during ice-age(s), followed by an incomplete recovery, possibly due to competition with emerging angiosperm species and a loss of range. Due to computational constraints and the exponential increase in the size of the parameter space with additional epochs, we did not consider more complex models of population size fluctuation. While it is likely that the true history of the species is more complex than the three-epoch model, this model may still be adequate to derive neutral expectations for present day population samples.

**Table 1:** Fitted model likelihoods for neutral, two-epoch and three-epoch demography models (see parameter meaning in Figure 1).

	T_B_	N_B_/N_0_	T_A_	N_A_/N_0_	log-likelihood	SE	LRT
**Three-epoch**	**0.041**	**0.155**	**0.214**	**3.793**	**-1088.38**	**0.30**	**4.52E-11**
	0.046	0.133	0.168	2.976	-1088.59	0.17	
	0.041	0.097	0.132	2.637	-1088.67	0.30	
	0.041	0.182	0.242	3.793	-1088.74	0.24	
	0.041	0.097	0.132	2.976	-1088.77	0.26	
	0.033	0.133	0.168	2.976	-1088.78	0.25	
	0.041	0.182	0.242	4.281	-1089.08	0.23	
	0.046	0.133	0.168	2.637	-1089.09	0.23	
	0.037	0.097	0.132	2.976	-1089.10	0.22	
	0.033	0.133	0.168	3.360	-1089.19	0.20	
	0.037	0.097	0.132	2.637	-1089.20	0.24	
	0.037	0.097	0.132	2.336	-1089.42	0.21	
	0.027	0.113	0.132	2.069	-1089.44	0.28	
	0.033	0.182	0.242	2.976	-1089.62	0.34	
	0.058	0.182	0.242	4.281	-1089.92	0.38	
	0.027	0.113	0.132	1.833	-1089.96	0.17	
	0.046	0.155	0.214	2.976	-1090.11	0.30	
**Two-epoch**	**-**	**-**	**1.421**	**12.847**	**-1114.43**	**0.35**	**6.14E-11**
	**-**	**-**	1.314	11.555	-1114.65	0.27	
	**-**	**-**	1.314	10.392	-1115.20	0.32	
	**-**	**-**	1.664	19.632	-1115.52	0.34	
	**-**	**-**	1.664	15.881	-1115.60	0.27	
	**-**	**-**	1.421	15.881	-1115.63	0.37	
	**-**	**-**	1.214	1 1.555	-1115.81	0.35	
	**-**	**-**	1.421	10.392	-1115.86	0.33	
	**-**	**-**	1.538	17.657	-1115.91	0.30	
	**-**	**-**	1.314	9.347	-1115.98	0.13	
	**-**	**-**	1.664	17.657	-1116.22	0.31	
	**-**	**-**	1.538	10.392	-1116.37	0.29	
**Constant Size**	**-**	**-**	**-**	**-**	**-1156.12**	**0.18**	

Best fit demographic models for each model class are shown in bold, competing models within 2 log likelihood units of the maximum likelihood model are given below. N_0 ,_ N_B_ and N_A_ refer to current, during bottleneck and before bottleneck effective population sizes, while T_A_ and T_B_ refer to time in 2N_0_ generations to the change from N_B_ to N_0_ and from N_A_ to N_B_ , respectively. SE stands for standard error while LRT stands for likelihood-ratio test.

Given the parameters for the three-epoch model fit to the data for all loci, we then considered whether any of the loci showed patterns of nucleotide polymorphism that were inconsistent with this neutral bottleneck expectation. The results for all loci are shown in Supplementary Material Table S8. At the *α* = 0.05 significance level, four loci (*c4h1b, cad, erf1-like* and *ccoaoemt*) showed an excess of mid-frequency alleles (higher than expected Tajima's D or Fu and Li's D), five (*cyp450-like, c3h-f4r6, erd3, pr4.1* and *myb3*) showed an excess of rare alleles (lower than expected Tajima's D or Fu and Li's D), three had an excess of polymorphisms (*agp4, wrky-like-2* and *pr4.3*) and six loci had fewer polymorphisms than expected (*axr, gatabp1, caf1, erd3, paeoemt* and *pp2c)*. One locus (*erd3*) had simultaneously low diversity and an excess of rare alleles. When the significance threshold was raised to α = 0.01 only *axr, c3h-f4r6, erd3, myb3* and *pp2c* were significant. Finally, only *pp2c* (less polymorphism than expected) and *myb3* (excess of mid-frequency alleles) were significant when a (conservative) Bonferroni correction for multiple testing was applied (see Supplementary Material Table S8).

These observations may be consistent with balancing or diversifying selection in the case of excess mid-frequency alleles, and selective constraint or a recovery of diversity after a recent selective sweep for loci with an excess of rare alleles (see Supplementary Material Table S1). Low and high diversity loci may simply indicate a lower or higher mutation rate at these loci than the average mutation rate considered over all loci (see Methods). However, low diversity is also expected if there has been little time for recovery of neutral polymorphism after a recent selective sweep.

### McDonald-Krietman Poisson Random Field analysis (MKPRF)

In our study, the posterior means of the selection coefficient distribution for each individual locus are of primary interest in order to evaluate the different selection hypotheses tested. Additionally, we asked whether disease candidate loci show a trend for selection that is distinct from other groups of candidate loci previously published [14], [15], since a central assumption for this work is that disease response loci are under stronger selection than most other loci in the genome.

MKPRF analysis was based on 32 of the disease response candidate loci in this study, 12 wood quality candidate loci [15] and 12 drought tolerance candidate loci [14], using publicly available (GenBank) spruce ESTs as an outgroup (*Picea glauca* and *Picea sitchensis*). It was immediately observed that the disease dataset contained four loci (*pr4.3, pr4.1, ldox-c* and *set-like-c*) that were strongly conserved (i.e. they had a highly significant deficit of amino acid replacements) and were poorly modeled as a single group together with the other disease candidates. We separated these loci into their own class and obtained the posterior distribution summaries shown in Figure 2A. Apart from the conserved *pr4.3, pr4.1, ldox-c* and *set-like-c*, results showed several other disease candidate loci with a significantly positive selection coefficient at α = 0.05. However, only *gatabp1* was significant at α = 0.01 (Figure 2B). None of the non-disease candidate loci showed a significant positive or negative selection coefficient distribution.

When the different groups of candidate loci were compared, the distribution of mean selection coefficients was clearly different. The wood quality and drought tolerance candidate loci were not in general under positive or negative selection, but the non-conserved disease candidate loci showed a clear trend of positive selection overall. The conserved disease candidate loci (*pr4.3, pr4.1, ldox-c* and *set-like-c*) showed strong negative selection and were clearly a distinct group. Together, these results show that the disease candidate loci studied here as a whole are evolving in a distinct manner from either the wood quality or drought tolerance candidate loci, with some disease candidate loci individually showing significant evidence for adaptive evolution.

### Species Isolation Model (IM)

For 24 of the disease candidate loci in this study and three of the drought tolerance candidates from previous work, we were able to amplify in single copy one or two gametes from the related pine species *Pinus sylvestris* (Scots pine). We noted that some loci harbored polymorphisms that were present in both species. This observation is unexpected since loblolly and Scots pine lineages diverged about 25 MYA [28], loblolly pine is found only in the southeastern United States while Scots pine's natural range is northern Europe and Asia, and there have been no reports of secondary contact between the species. One possibility is that close paralogs are being amplified in some individuals of each species. If this is the case, these polymorphisms identified would be paramorphisms rather than true SNPs. Paramorphisms do not necessarily exhibit Mendelian segregation; therefore, they won't be easily converted to informative genetic markers [29]. Acting on this expectation, we tested for Mendelian segregation of these loci within the two available mapping populations for loblolly pine with 90 progeny each, as well as families of the FBRC-family based association population of loblolly pine (∼16 progeny per family on average). Of the three genes that were identified to contain shared polymorphisms (*lp5, pr4.1, wrky-like-1*) *lp-5* was successfully mapped to linkage group 3 using mapping populations and did not show a significant deviation from expected Mendelian patterns in the FBRC-families it was segregating in (lowest P = 0.087,χ^2^ = 2.92(df = 1)). *pr4.1* was not segregating in the mapping populations, however, when checked for their Mendelian segregation patterns between the parents and progeny of the FBRC-association population families, it also did not show a significant deviation from expected Mendelian patterns (lowest P = 0.10, χ^2^ = 2.2 (df = 1)). *wrky-like-1* displayed usually low values for the segregation ratios, displaying a marked decrease in heterozygote individuals detected. Regardless, all the p-values for each of 5 families that were segregating displayed P-values over the 0.05 threshold (lowest P = 0.072, χ^2^ = 3.25 (df = 1)) (See Supplementary Material- Results S1).

Assuming, these polymorphisms are in fact true SNPs and not paramorphisms, it is of particular interest whether or not these shared polymorphisms have been preserved in both taxa as a result of the long-term action of balancing selection. With no reason to suspect gene flow between the two taxa, the expectation of shared polymorphisms can be estimated from the simple isolation model (IM) of speciation, as implemented in the WH program. Provided the isolation model of speciation is a good fit to the data overall, deviations from model expectations at individual loci would suggest population genetic forces other than mutation and drift are required to explain the observations.

First, the WH program was run to estimate overall population sizes and time of divergence. The WH test statistic computed by the program indicated a good overall fit of the isolation model with 61% of simulated datasets generating a higher than expected value of the test statistic. The time of divergence was estimated at 0.86*N_1_* generations with an ancestral population size 6.72 times the current size of loblolly pine. The Scots pine population size was estimated at 1.03 times the current size of loblolly pine. Second, simulated datasets were generated using these parameters to estimate an expected number of species specific polymorphisms, shared polymorphisms, and number of fixed differences for each locus.

Using a standard goodness-of-fit G-test, six loci (*axr, gatabp1, lp5, pcna, sams2* and *set-like-c*) showed a poor fit to the isolation model at the 0.05 level (Bonferroni corrected) and five at the 0.01 level (Table 2). *axr* and *gatabp1* have too few polymorphisms in loblolly pine and too many fixed differences. This is in agreement with the low nucleotide diversity found in the SFS analysis for these two loci. The excess of fixed differences indicates a rapid rate of substitution and is consistent with directional selection. *pcna* has too many polymorphisms in loblolly pine, and *sams2* and *set-like-c* have too many polymorphisms in Scots pine; all three loci also appear to have too few fixed differences between the species. Too few fixed differences could be explained by balancing selection acting in only one of the species, reducing the rate of fixation between species while increasing or maintaining polymorphism within that species. Lack of balancing selection in the other species would allow the ancestral alleles to be lost by drift and shared polymorphisms would not be maintained. Alternatively, purifying selection against new variants could explain the reduced level of fixed differences, but this is not consistent with elevated polymorphism levels in either species or the fact that the SFS analysis did not reveal any excess of low frequency polymorphisms for these three loci. Conversely, the MKPRF analysis found that *set-like-c* was highly conserved, which is consistent with purifying selection.

**Table 2:** Isolation model (IM) for Scots and loblolly pines.

*Locus*	*S_Xtaeda_* a		*S_Xsylv._* b		*S_shared_* c		*S_fixed_* d		∑*G* e	*df*	*P* f	Sig.
	*Obs*	*Sim*	*Obs*	*Sim*	*Obs*	*Sim*	*Obs*	*Sim*				
*4cl*	11	7.18	2	1.18	0	0.32	2	6.31	6.90	4	0.23	
***axr***	**1**	**7.71**	**2**	**2.25**	**0**	**0.25**	**13**	**5.65**	**17.08**	**4**	**1.84×10^-3^**	*
*bhlh62-like*	7	4.91	0	1.41	0	0.16	3	3.53	3.99	4	1.23	
*ccoaoemt*	14	12.5	0	0	0	0	10	11.49	0.40	4	14.30	
*cesA3*	4	3.83	0	0.63	0	0.17	4	3.36	1.75	4	5.03	
*cyp450-like*	10	5.5	0	0	0	0	0	4.79	13.03	4	-	
*enth1-like*	11	8.83	0	2.53	0	0.28	7	6.36	6.18	4	0.35	
*erd3*	8	15.63	0	0	0	0	22	14.37	8.02	4	0.12	
*erebp-like*	22	23.07	1	6.61	0	0.73	24	16.59	11.85	4	0.07	
*erf1-like*	6	10.41	0	0	0	0	14	9.58	4.01	4	1.22	
***gatabp1***	**0**	**8.34**	**1**	**2.39**	**0**	**0.27**	**29**	**6**	**89.62**	**4**	**1.6×10^−18^**	*
*gatabp2*	11	12.76	3	3.65	0	0.41	12	9.18	1.98	4	10.03	
*ldox-a*	14	11.78	3	3.37	0	0.38	7	8.47	1.46	4	13.01	
*ldox-c*	18	16.29	0	0	0	0	12	13.71	0.39	4	22.16	
***lp5***	**20**	**13.25**	**2**	**3.8**	**4**	**0.42**	**1**	**9.53**	**27.39**	**4**	**1.7×10^−5^**	*
*mybs3-like*	5	5.21	0	0	0	0	5	4.79	0.01	4	24.71	
*myb3-psd*	11	14.19	1	4.09	0	0.45	17	10.27	8.71	4	0.35	
*nac1*	19	15.65	1	4.51	0	0.5	12	11.34	5.71	4	1.55	
*pal1*	6	8.34	5	2.39	0	0.27	6	6	3.43	4	1.73	
***pcna***	**16**	**8.83**	**0**	**2.53**	**0**	**0.28**	**2**	**6.36**	**14.38**	**4**	**6.2×10^−3^**	*
*pr4.1*	18	18.59	4	5.36	1	0.59	15	13.46	0.80	4	22.96	
*rd21a*	25	21.88	0	0	0	0	17	20.12	0.94	4	9.00	
***sams2***	**6**	**6.87**	**8**	**1.97**	**0**	**0.22**	**0**	**4.94**	**20.81**	**4**	**3.5×10^−4^**	*
*set-like-b*	6	12.27	8	3.51	0	0.39	11	8.83	9.43	4	0.24	
***set-like-c***	**19**	**18.65**	**18**	**5.34**	**0**	**0.59**	**1**	**13.42**	**39.26**	**4**	**6.2×10^−8^**	*
*tps-like*	12	12.76	3	3.65	0	0.41	11	9.18	1.32	4	13.94	
*wrky-like-1*	7	5.4	1	1.55	2	0.17	1	3.88	9.87	4	0.53	

a Number of polymorphisms detected in loblolly pine sequences (N = 27–32).

b Number of polymorphisms detected in Scots pine sequences (N = 2).

c Number of shared polymorphisms between loblolly pine and Scots pine.

d Number of fixed differences between loblolly pine and Scots pine.

e Sum of the chi-squared values for each class of variation per locus.

f Significance of the chi-squared values.

*Indicates *P*<0.05 for the significance of the results, after Bonferroni correction for multiple testing. Bonferroni corrected threshold for α = 0.05 is 1.85**×**10^−3^.

The results from comparing observed values to 10,000 simulations under the fit model (see text) are also presented. *P*-values reported in the table below can be multiplied by the number of loci analyzed (27) to provide a Bonferroni correction for multiple tests. Loci that deviated from the fit model are shown in bold.

Of the three loci with shared polymorphisms, only *lp5* showed a significantly poor fit to the isolation model, with an excess of shared polymorphisms, excess of polymorphisms in loblolly pine, and a lack of fixed differences. This seems consistent with balancing selection maintaining ancestral alleles in both species as well as increasing diversity within loblolly pine. However, an excess of mid-frequency alleles, which might be expected under some balancing selection scenarios, was not observed in the SFS analysis.

The neighbor-joining tree plots of the polymorphism and divergence data from the loci are available as Supplementary Material in file Results S2.

## Discussion

In this study, we have investigated a set of candidate genes for disease response and plant-pathogen interaction in loblolly pine, noting that two prominent theories describing the evolution of genes involved in these interactions imply strong selective forces acting at these loci. In principle, a collection of genes chosen as candidates based on functional or predicted annotations of homologs in other plant systems may be further screened for evidence of selection that would support their role in the evolution of disease responses. In the absence of genomic resources and tools for extensive genetic and biochemical investigations, analyzing nucleotide data from population samples for evidence of selection provides a valuable alternative. Here, we present several population genetic analyses on nucleotide polymorphism and substitution data for 41 disease response candidate gene loci as well as 28 additional loci from previous studies in loblolly pine. While each analysis alone provides only suggestive evidence with potentially multiple explanations, taken together the analyses are complimentary as the conclusions of one analysis may be supported or confirmed by another while alternative explanations are excluded.

Polymorphism data within loblolly pine provided evidence regarding the recent evolution of the species (demography) as well as identified recent selective events at individual loci. In contrast, substitutions at replacement and silent sites provided evidence for the rate of evolution at individual loci at a broader temporal scale, with a large rate of amino acid replacements indicating rapid evolution driven by selective fixations as opposed to a small rate which would indicate selective constraint. Finally, if two species have been reproductively isolated for a long period of time, shared polymorphisms are not expected. Since we observed shared polymorphisms between loblolly pine and Scots pine (two allopatric species that diverged at least 25 MYA [28]) at some loci, we investigated the species isolation model to assess whether these shared polymorphisms indicate the maintenance of ancestral alleles in the extant populations of each species, as we might expect under balancing selection.

Analysis of the site frequency spectra across the 69 loci (41 disease candidates in this study as well as 28 from previous studies, see Table S2 from Supplementary Material) found four loci (*c4h1b, cad, erf1-like* and *ccoaoemt*) with an excess of mid-frequency alleles and five loci (*cyp450-like, c3h-f4r6, pr4.1, myb3-psd* and *erd3*) with an excess of rare alleles. As summarized in Supplementary Table S1, the former is expected for balancing and diversifying selection and is supportive of the trench-warfare model, while the latter may indicate a recent selective sweep as may be expected under the arms-race model. Alternatively, an excess of rare alleles may simply indicate a strong background selection preventing mutations from reaching any appreciable frequency in the population. *erd3* had also a low level of nucleotide diversity overall in addition to rare allele excess. This would be expected for a locus which either recently completed a selective sweep but has not yet accumulated new mutations or is currently being swept of variation by a selective sweep but fixation of the selected allele has not yet occurred. *erd3* is a member of *early-response-to-drought* family of proteins initially identified in *Arabidopsis.* To further assess the possibility of a selective sweep at this locus, sequencing a larger region and distinguishing ancestral and derived alleles would allow the spatial distribution of allele frequencies to be assessed for the pattern expected under a selective sweep model [35] using more advanced methods such as that of Kim and Stephan [36].

The McDonald-Krietman Poisson Random Field model was used to investigate evidence for directional selection or selective constraint, over a longer period of time than can be studied with within-species polymorphism data alone. The most interesting result of this analysis is the difference between the disease candidate loci considered as a group vs. the wood quality and drought tolerance loci from previous studies. Both wood quality and drought tolerance groups produced a posterior distribution of the mean selection coefficient across loci well centered around zero, indicating that both groups, as a whole, were neither evolving rapidly under directional selection nor under strong selective constraint. In contrast, the disease candidate loci clearly showed two distinct groups, one under constraint and one evolving rapidly (i.e. with an excess of amino acid replacements). When these subgroups were analyzed separately, all four loci (*pr4.3, pr4.1, ldox-c* and *set-like-c*) in the constrained group were found to have a highly significant negative selection coefficient (more than 99% of posterior samples lower than zero), while several loci in the rapidly evolving group showed a significantly positive selection coefficient (more than 95% of posterior samples higher than zero). One locus, *gatabp1*, was highly significant with more than 99% of the posterior samples higher than zero. A central premise of this study is that disease resistance loci are likely to be under some form of strong selection due to the inherently competitive interaction between host and pathogen. The MKPRF analysis presented here clearly demonstrates a difference between these disease candidate loci and other groups of candidate loci, supporting this assumption and further motivating a population genetic approach to disease candidate gene selection and identification.

The application of the isolation model (IM) to loblolly pine sequence data revealed some loci (in particular *lp5*, a drought-response gene) with an excess of shared polymorphism with Scots pine. Although there is a possibility that these shared polymorphisms were generated due to amplification from paramorphic loci, it is unlikely since the segregation patterns for the locus did not show any deviation from Mendelian expectations. If there was amplification from paramorphisms, the segregation patterns were not expected to be robust [37].

According to the estimates from diffusion calculations [38], the expected time for shared polymorphisms loss between two species is 2.77 *N_e_* generations under neutrality and gets even shorter (around 1.7 *N_e_* generations) when either of the species experiences a bottleneck. With the effective population size and generation time estimates available for Scots pine in the literature [39], the expectation for the time to fixation of shared polymorphisms is less than 10 million years. Furthermore, the MKPRF estimate of *τ* –the number of generations since divergence divided by 2*N_e_* (estimated from all sites) – is 2.74 (credible intervals: 2.07-3.51) indicating at least twice the necessary time has passed since divergence between these species. An alternative explanation for shared polymorphisms is a possible introgression of alleles from loblolly pine into Scots pine or vice-versa. However, if introgression had occurred at the *lp5* locus, we would expect to see a deficit of species-specific polymorphisms yet here we see an excess of these polymorphisms in addition to shared polymorphisms. Thus, balancing selection acting on this locus seems a more plausible hypothesis.

While each analysis was chosen to investigate selective hypotheses utilizing different aspects of the data, none of these analyses were perfect in application. The SFS analyses incorporated the most data as this analysis did not require outgroups and data from earlier studies could be readily used to fit the demographic model. However, the three-epoch model considered may be too simple to describe the demographic history of loblolly pine, and deviations from the expectation under this model must still be interpreted with caution. The MKPRF analysis is perhaps the most robust method employed in this study as the model does not rely on assumptions such as a constant size population. However, the method by itself can only distinguish background and directional selection from neutrality, and selective forces must have persisted over longer periods of evolutionary time for the method to have high power. For the isolation model, available implementations unfortunately do not take into account population size changes within the extent species history. Thus, the model inherently conflicts with the results demonstrated from the site frequency analysis that the effective size of loblolly pine has clearly fluctuated since the most recent common ancestor of the species. The lack of shared polymorphisms with Scots pine at nearly all loci but a few examined here indicates that the most recent common ancestor of loblolly alleles occurs after the split (in forward time) of loblolly and Scots pine from the ancestral species. Additionally, our data are quite limited for this analysis since only two samples were available to discover polymorphisms in the outgroup (although this is standard practice in the application of this method). Nevertheless, the isolation model clearly demonstrated that at least the *lp5* locus appears to have ancestral alleles preserved in both loblolly and Scots pine, providing strong support for balancing selection. There is no other evidence that the other two loci (*pr4.1* and *wrky-like-1*) that also carry shared polymorphisms are influenced by a similar evolutionary trend. The violations of the strict isolation model in our application would make it less likely to observe this result by chance. Although *lp5* was *not* a prospective disease resistance candidate, the implications of balancing selection within this locus prompts further investigations on the function of this gene. Furthermore, the sequencing of a large number of disease candidate genes in this study have provided evidence for both *arms-race* and *trench-warfare* models of plant-pathogen interaction and identified genes for further studies aiming at distinguishing modes of disease response evolution in forest trees.

Finally, we note that the most serious limitation of this work is the short length of the sequence obtained from each locus. Longer sequence at each locus would provide more polymorphisms and substitutions and increase power and accuracy of all analyses. Additional outgroup samples for the isolation model analysis would provide more opportunity to observe shared polymorphisms to detect balancing selection as well as provide more data to produce more accurate model estimates. However, a strength of our study is the large number of loci analyzed and this compensates well for the limitation of short sequence at each locus. A large number of independent loci are necessary to obtain accurate estimates of neutral model parameters which are critical to detect meaningful deviations from neutrality.

In conclusion, we note that while both our data and analyses methods have limitations, this study demonstrates a novel approach for identifying and refining candidate genes for disease response or plant-pathogen interactions, by exploiting the implication of natural selection inherent in these biological processes. Furthermore with the analytical framework provided here, these data and results can be improved and tested further as more data is collected, even at non-candidate loci. More data at non-candidate loci can provide better estimates of species parameters such as neutral mutation rates, divergence times, and population sizes which would allow for better definition of expectations under neutrality. Thus, especially for loblolly pine and other forest trees which are poorly suited for more direct studies of plant-pathogen interactions, but ideal in many respects for population studies, this approach holds promise to further our understanding of specific disease resistance mechanisms as well as opportunity to study plant-pathogen interactions in natural populations.

## Supporting Information

Results S1 Mendelian Segregation Analysis of the loci that displayed shared polymorphisms between species, within FBRC population families that they segregate in. The family sizes are variable between 15 and 18. A Gtest with Williams correction was conducted, and the P-value was calculated according to chi-squared distribution with 1 degree of freedom (threshold for 0.05 significance is 3.84).
(0.04 MB XLS)

Results S2 NJ-tree plots for each loci.
(1.70 MB ZIP)

References S1 References associating candidate genes with disease resistance phenotypes.
(0.04 MB DOC)

Table S1 Theoretical expectations under the tests performed and the conclusions on selection patterns according to the test results presented in this manuscript.
(0.03 MB DOC)

Table S2 Selections and their origins Geographical origin of trees and relatedness in second generation crosses. Isolate IDs are provided as descriptors at GenBank accession numbers. For second generation trees we give between parentheses the ID of their parental trees, when parents were included in the sample as first selections. NA: data not available.
(0.05 MB DOC)

Table S3 The list of motifs identified in the putative promoter sequences investigated. Regulatory elements, identified by PLACE database in the consensus sequences of (a) AGP-like promoter, (b) PCBER promoter, (c) AEOMT promoter, (d) CCoAOMT promoter, (e) Chitinase promoter, (f) PR10 promoter.
(0.11 MB DOC)

Table S4 Primer sequences for the amplification products used for the diversity screen from the target disease resistance loci, listed according to their locus acronyms and NCBI-GI numbers of the templates used for primer design. Chitinase promoter and PR10 promoter template sequences were kindly provided by Dr. John Davis, UFL-Gainesville.
(0.06 MB DOC)

Table S5 Locus statistics for sequences from each loci.
(0.31 MB DOC)

Table S6 MKPRF input table
(0.06 MB DOC)

Table S7 WH input table
(0.05 MB DOC)

Table S8 Using the best fit demographic model, common summary statistics of the observed site frequency spectrum are compared with neutral expectations under the model. The quantiles column shows the proportion of simulated values generated under the model which were less than the observed values. Quantiles less than 0.025 or greater than 0.975 indicate significantly low or high values of the respective summary statistics.
(0.13 MB DOC)
