# Supplementary figures and images for: Evolution of Disease Response Genes in Loblolly Pine: Insights from Candidate Genes
Source: PLoS One. 2010 Dec 6;5(12):e14234. doi: 10.1371/journal.pone.0014234 (PMC2997792; doi:10.1371/journal.pone.0014234)

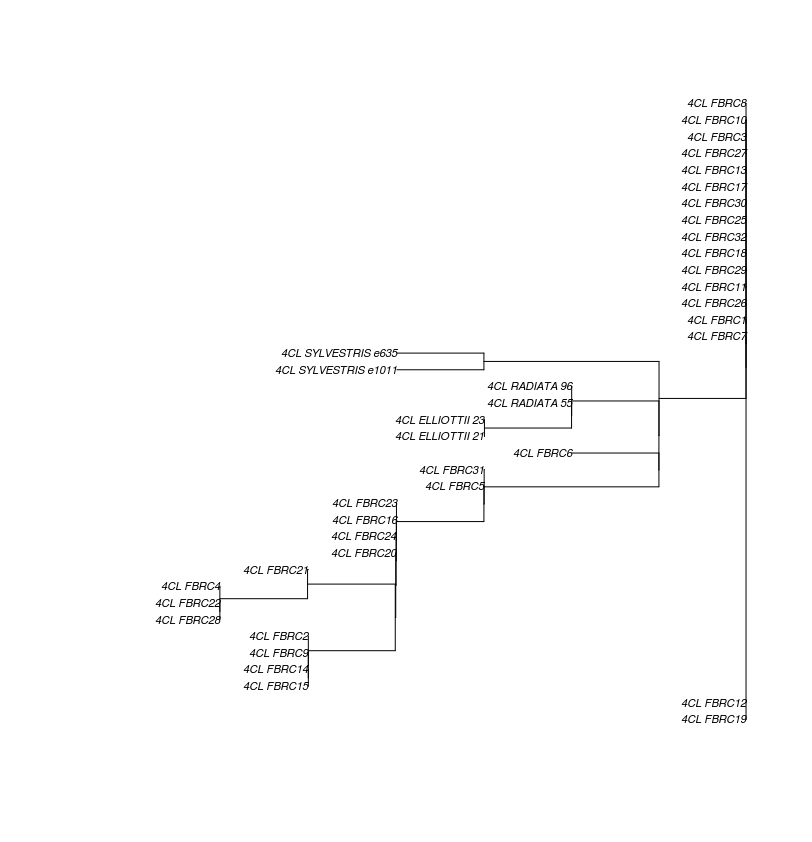

Supplement: Results S2 — NJ-tree plots for each loci. (1.70 MB ZIP) [file pone.0014234.s002.zip › plots/4cl.png]

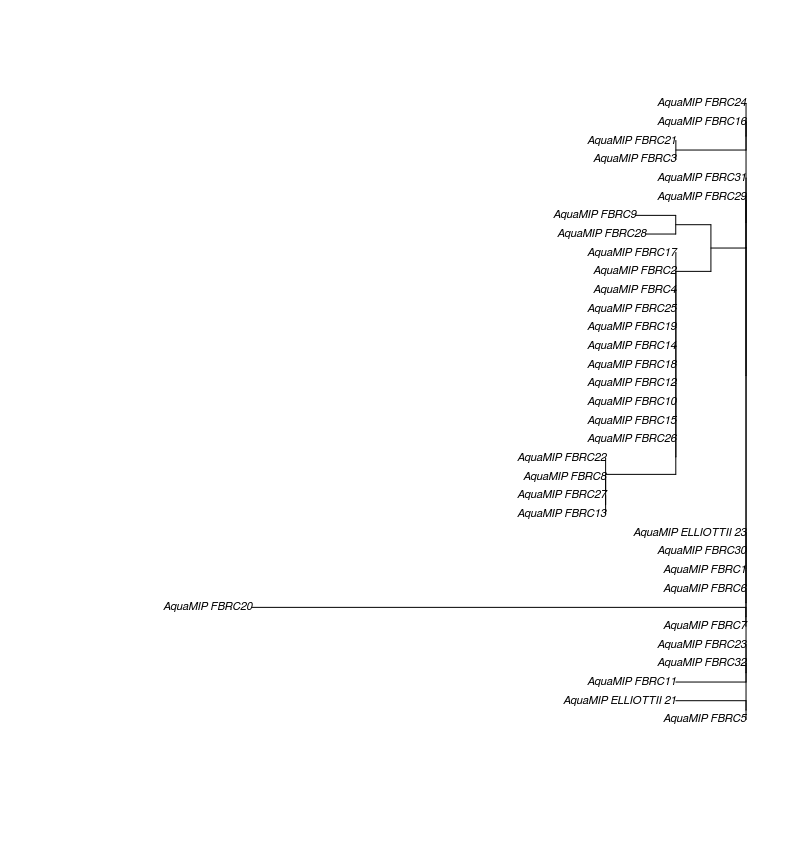

Supplement: Results S2 — NJ-tree plots for each loci. (1.70 MB ZIP) [file pone.0014234.s002.zip › plots/aquamip.png]

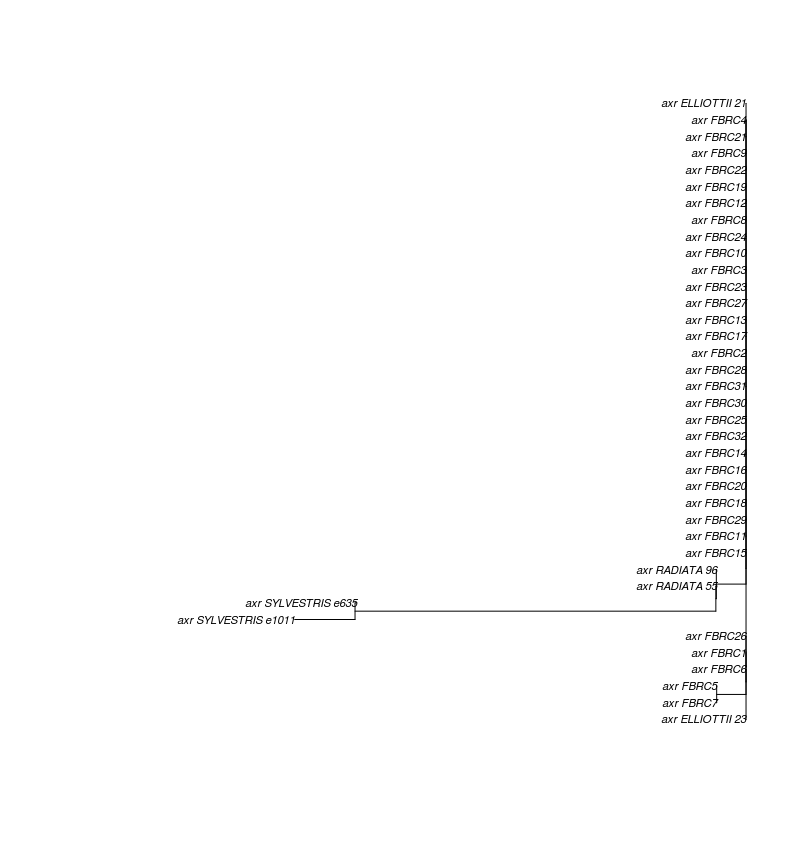

Supplement: Results S2 — NJ-tree plots for each loci. (1.70 MB ZIP) [file pone.0014234.s002.zip › plots/axr.png]

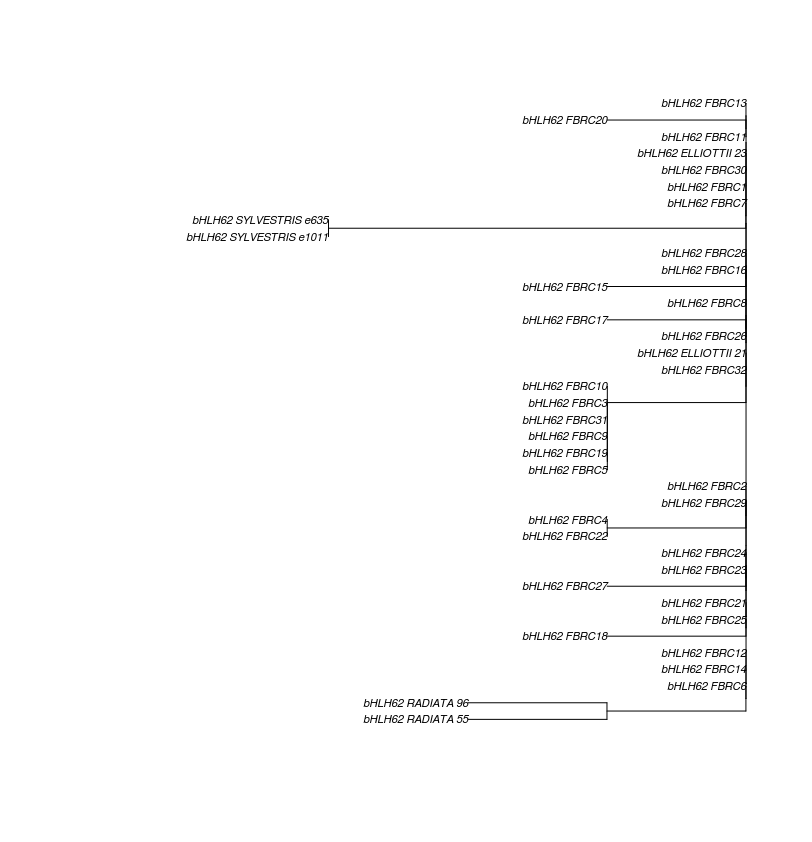

Supplement: Results S2 — NJ-tree plots for each loci. (1.70 MB ZIP) [file pone.0014234.s002.zip › plots/bhlh62-like.png]

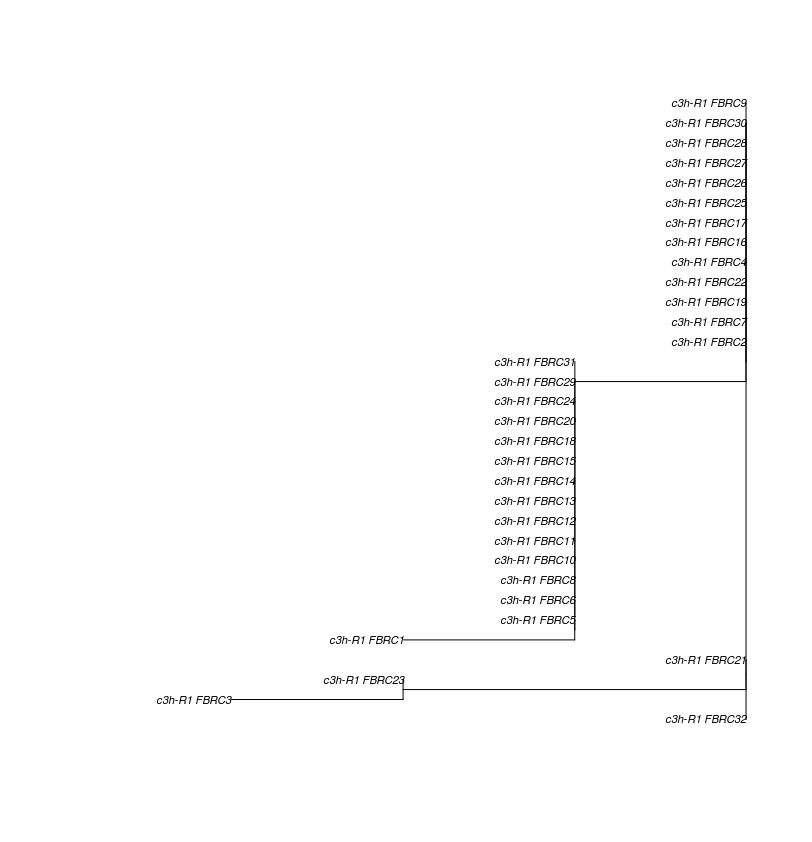

Supplement: Results S2 — NJ-tree plots for each loci. (1.70 MB ZIP) [file pone.0014234.s002.zip › plots/c3h.png]

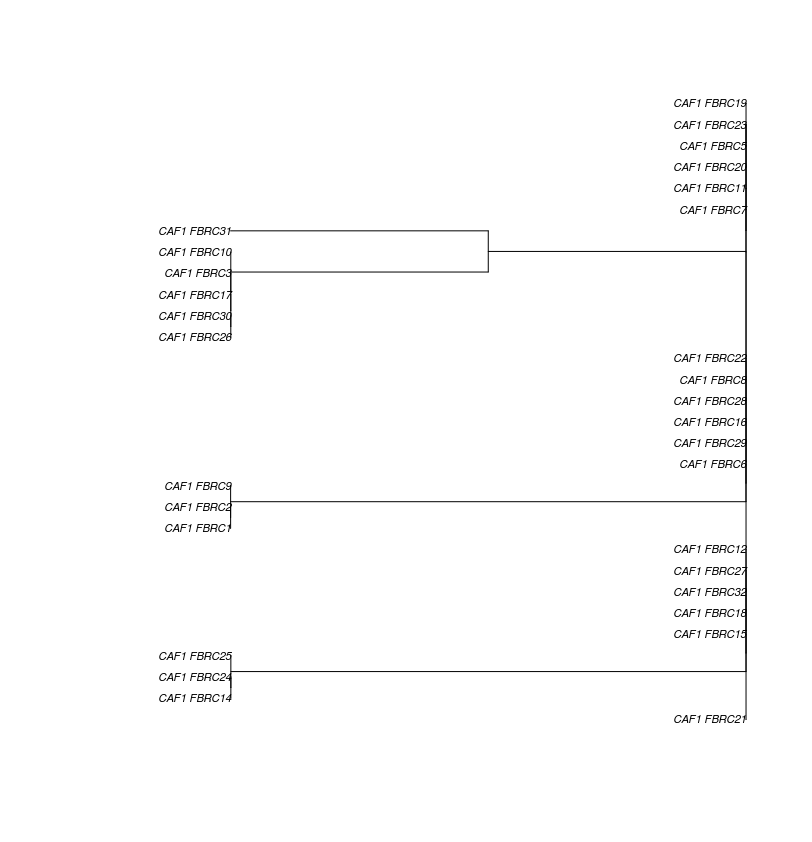

Supplement: Results S2 — NJ-tree plots for each loci. (1.70 MB ZIP) [file pone.0014234.s002.zip › plots/CAF1.png]

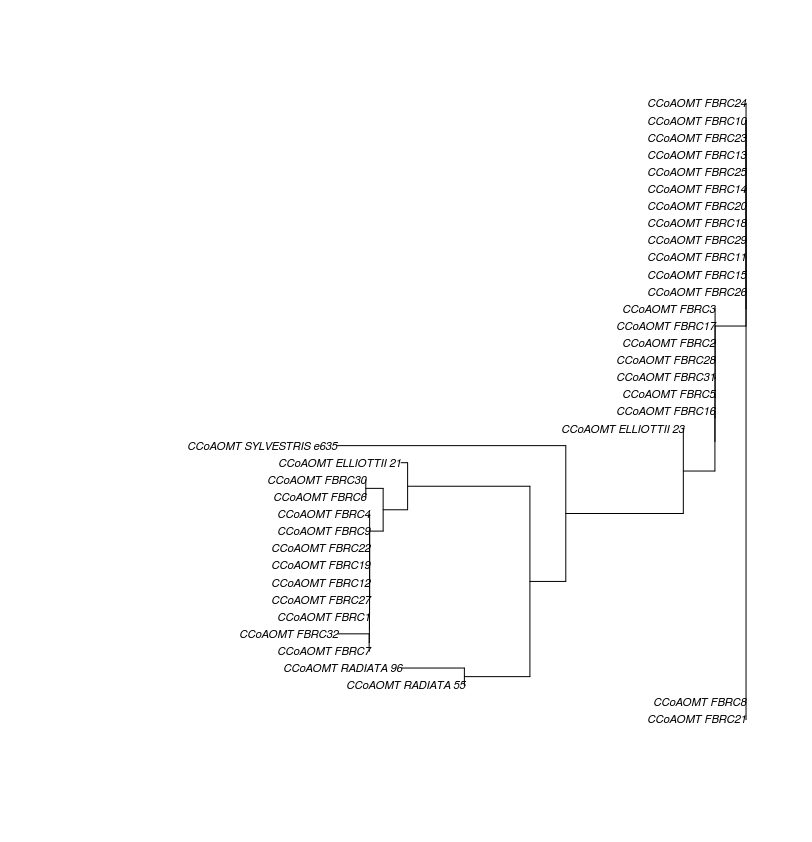

Supplement: Results S2 — NJ-tree plots for each loci. (1.70 MB ZIP) [file pone.0014234.s002.zip › plots/ccoaoemt.png]

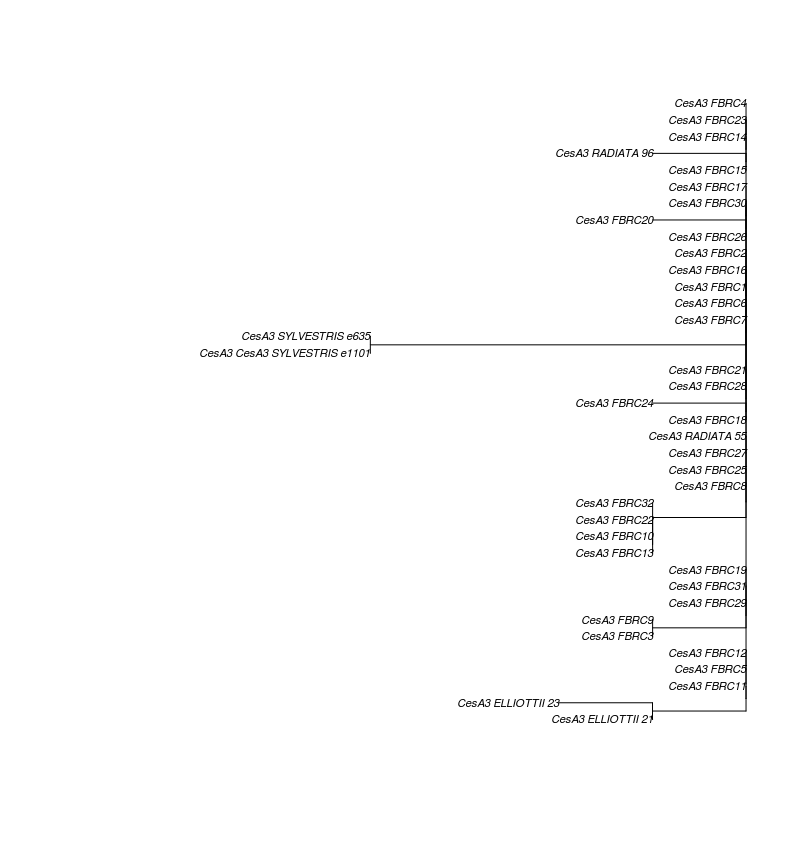

Supplement: Results S2 — NJ-tree plots for each loci. (1.70 MB ZIP) [file pone.0014234.s002.zip › plots/cesa3.png]

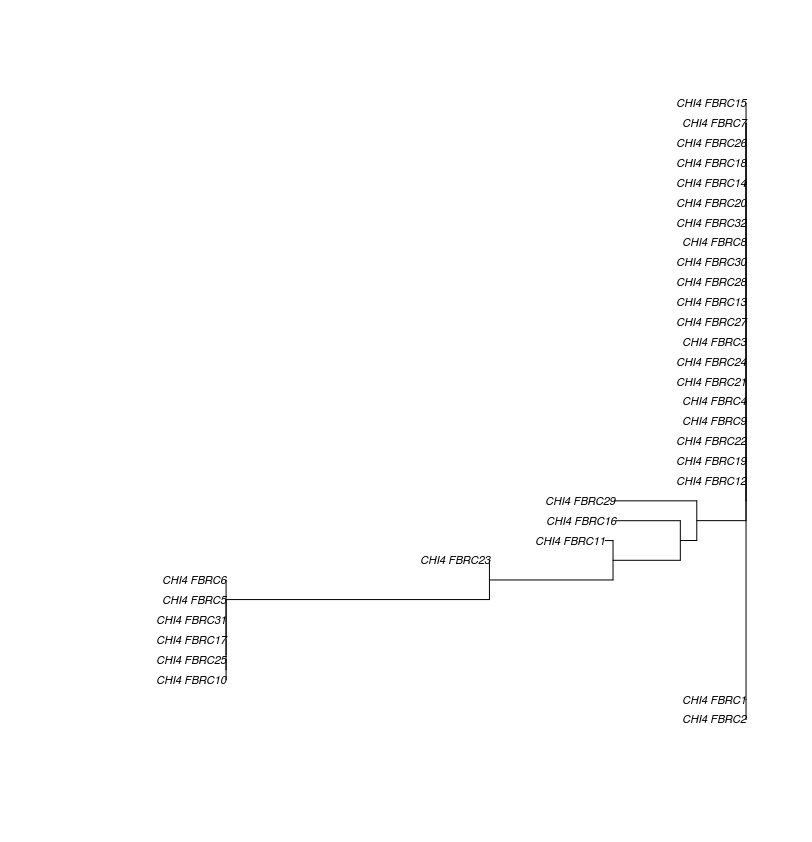

Supplement: Results S2 — NJ-tree plots for each loci. (1.70 MB ZIP) [file pone.0014234.s002.zip › plots/chi4-like.png]

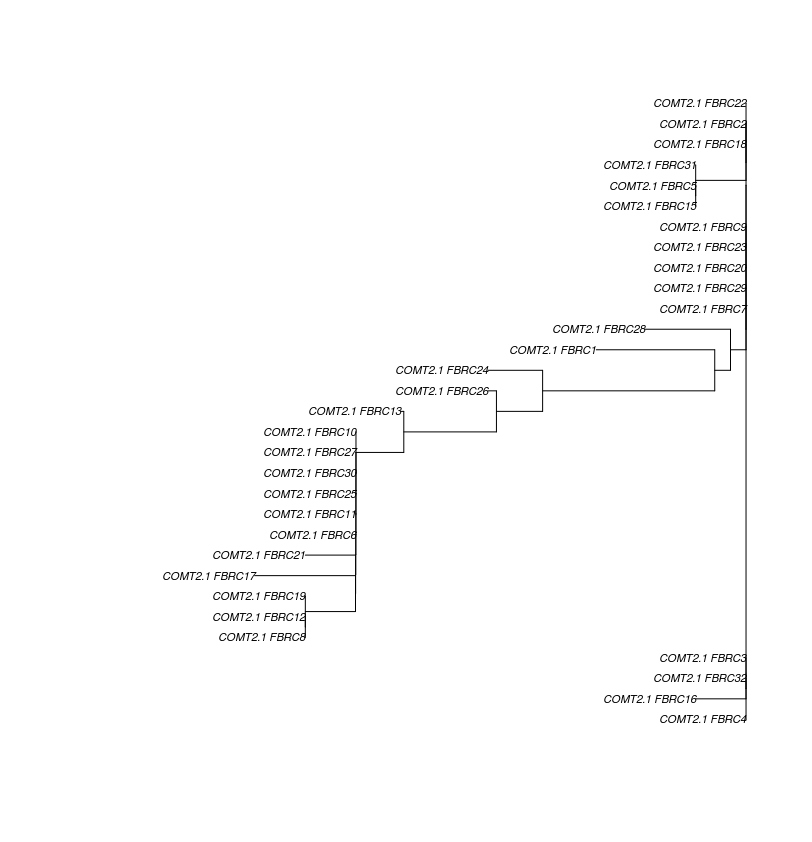

Supplement: Results S2 — NJ-tree plots for each loci. (1.70 MB ZIP) [file pone.0014234.s002.zip › plots/comt2.png]

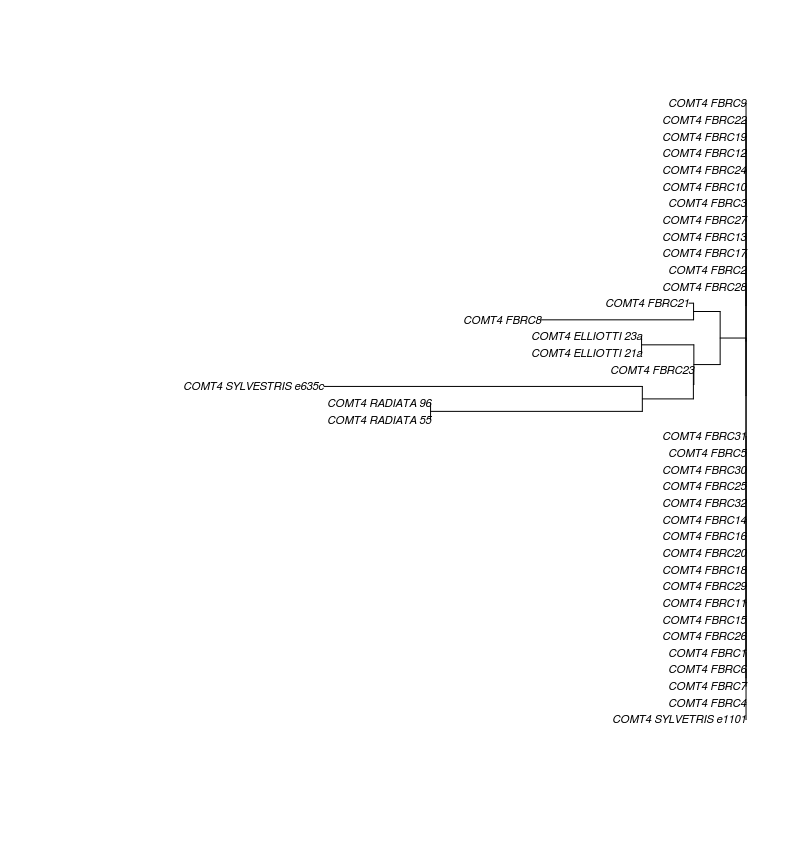

Supplement: Results S2 — NJ-tree plots for each loci. (1.70 MB ZIP) [file pone.0014234.s002.zip › plots/comt4.png]

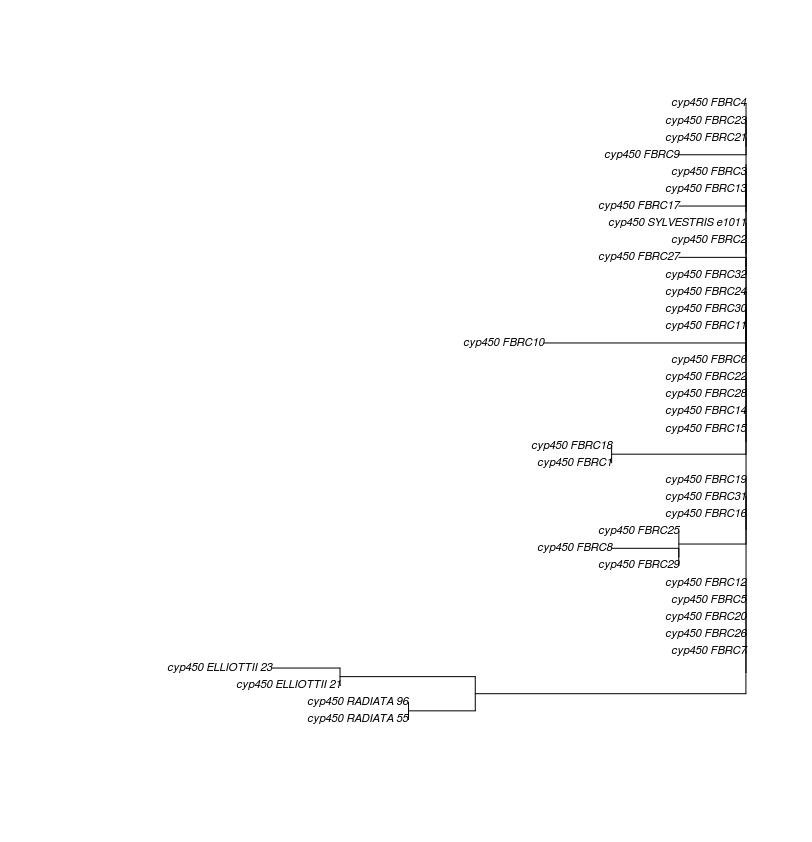

Supplement: Results S2 — NJ-tree plots for each loci. (1.70 MB ZIP) [file pone.0014234.s002.zip › plots/cP50-like.png]

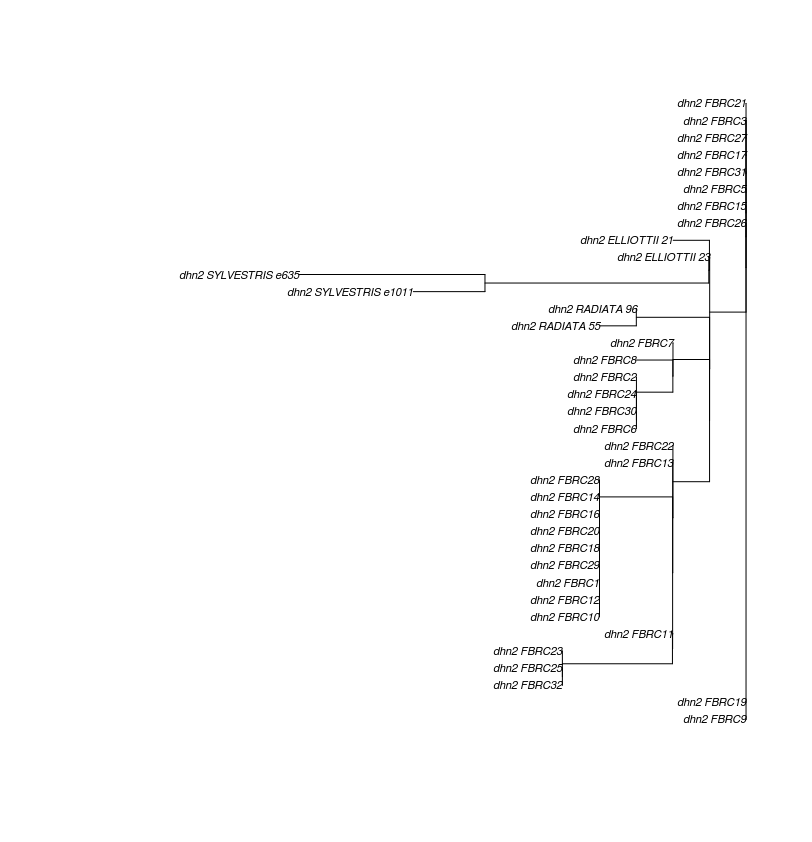

Supplement: Results S2 — NJ-tree plots for each loci. (1.70 MB ZIP) [file pone.0014234.s002.zip › plots/dhn2.png]

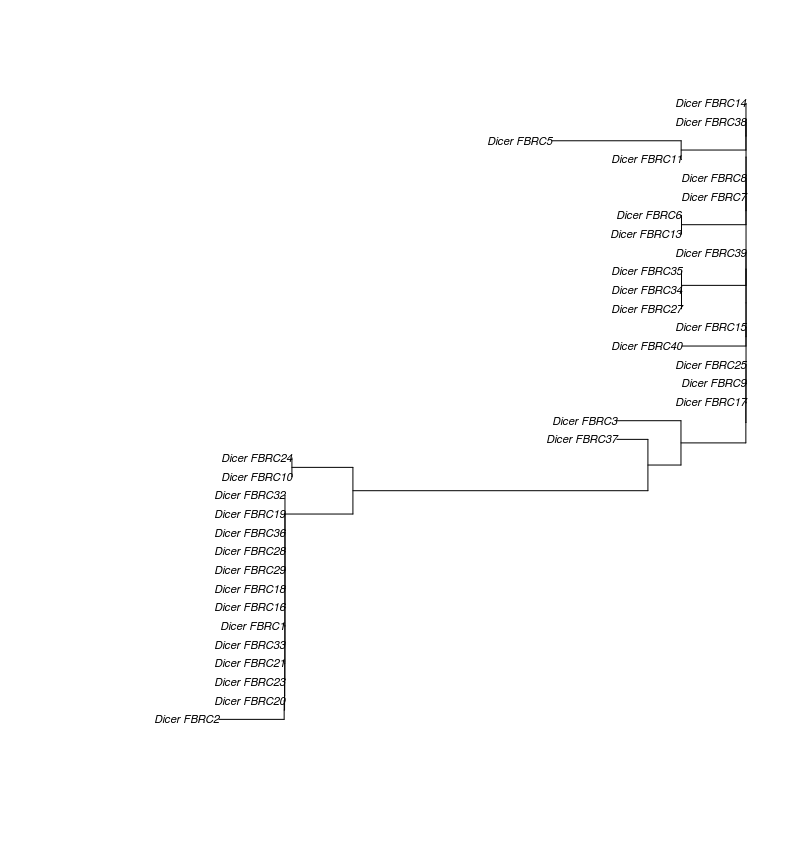

Supplement: Results S2 — NJ-tree plots for each loci. (1.70 MB ZIP) [file pone.0014234.s002.zip › plots/dicer-like.png]

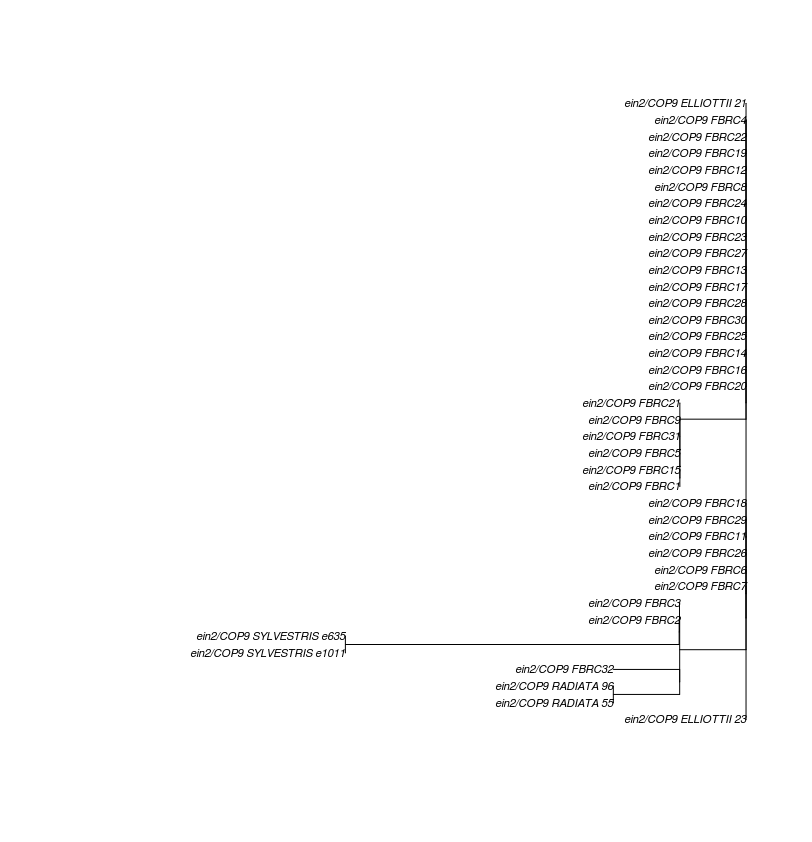

Supplement: Results S2 — NJ-tree plots for each loci. (1.70 MB ZIP) [file pone.0014234.s002.zip › plots/ein2.png]

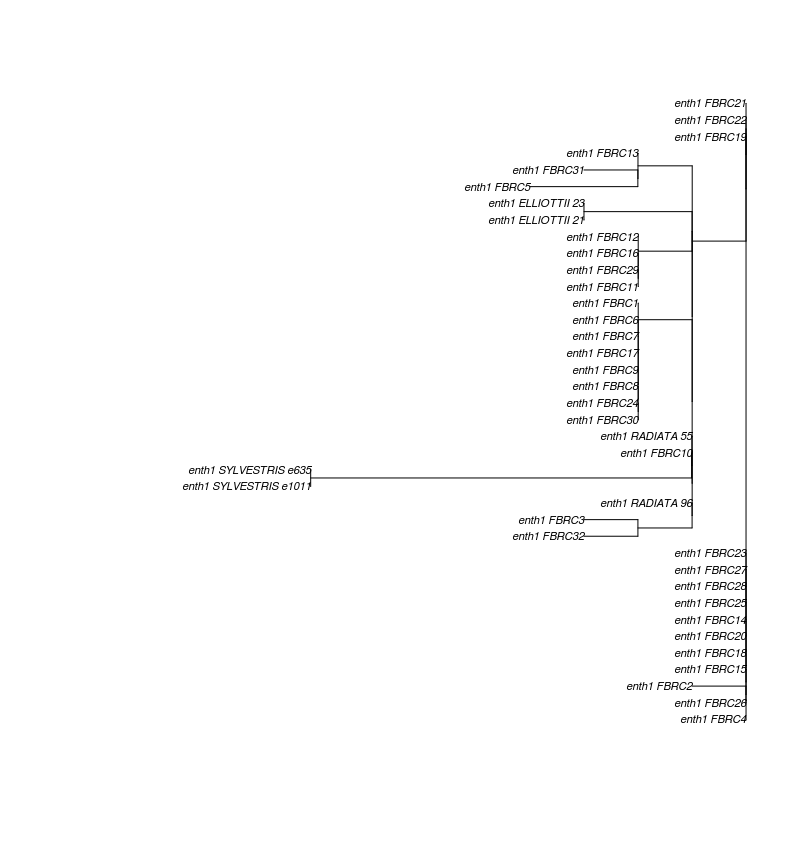

Supplement: Results S2 — NJ-tree plots for each loci. (1.70 MB ZIP) [file pone.0014234.s002.zip › plots/enth1.png]

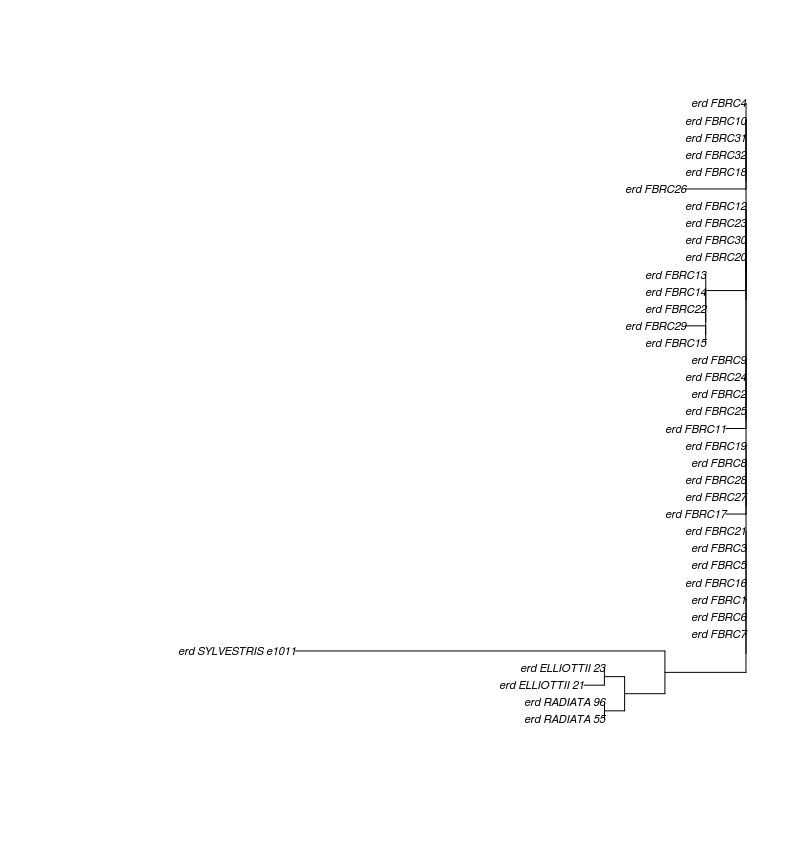

Supplement: Results S2 — NJ-tree plots for each loci. (1.70 MB ZIP) [file pone.0014234.s002.zip › plots/erd3.png]

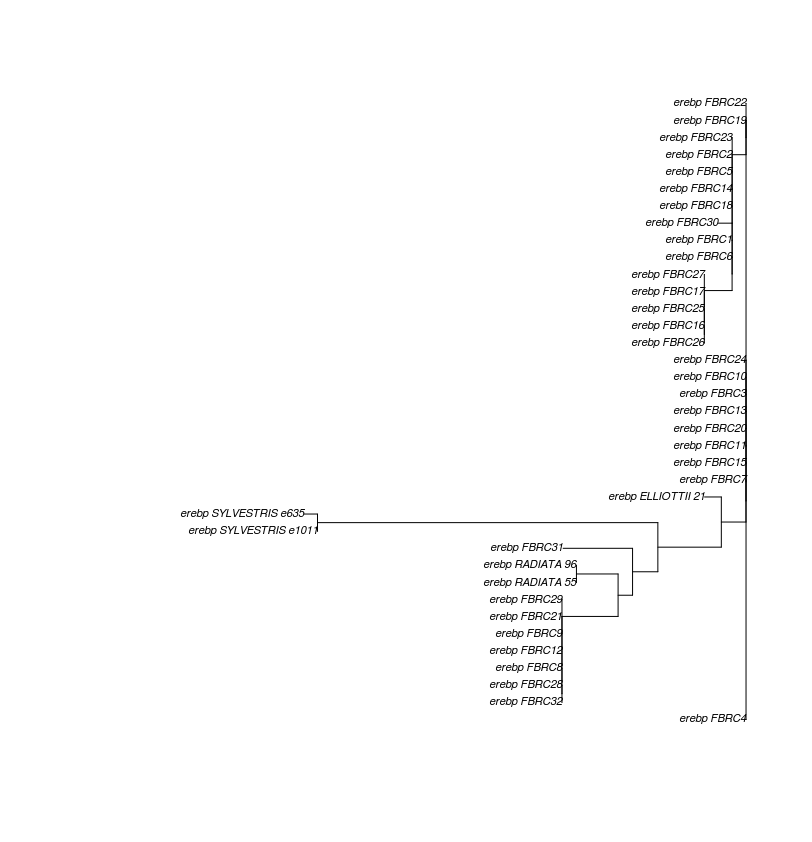

Supplement: Results S2 — NJ-tree plots for each loci. (1.70 MB ZIP) [file pone.0014234.s002.zip › plots/erebp.png]

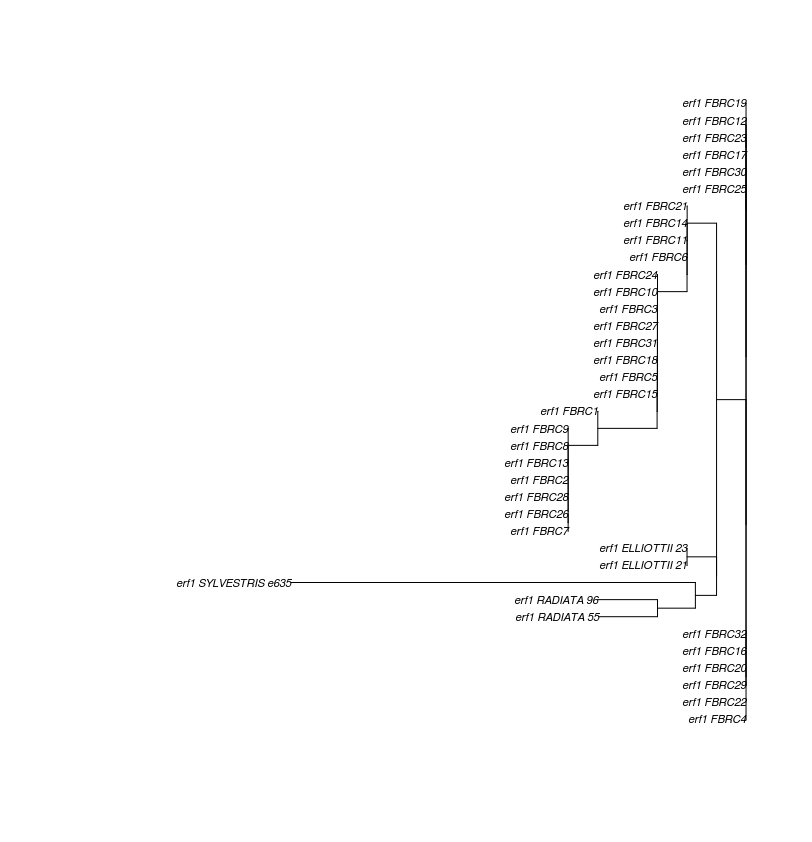

Supplement: Results S2 — NJ-tree plots for each loci. (1.70 MB ZIP) [file pone.0014234.s002.zip › plots/erf1.png]

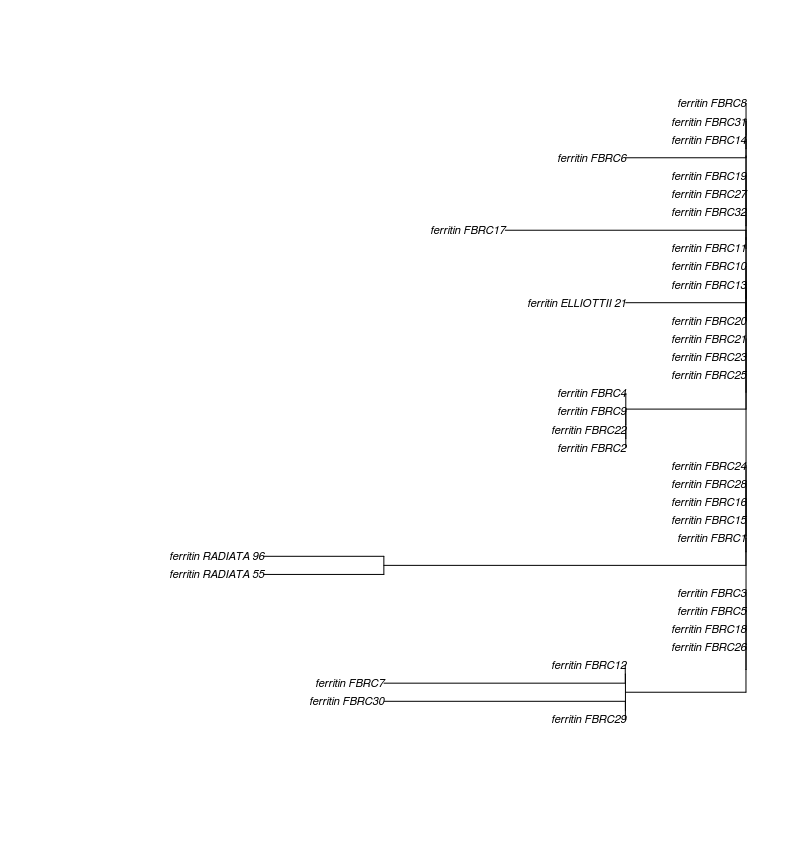

Supplement: Results S2 — NJ-tree plots for each loci. (1.70 MB ZIP) [file pone.0014234.s002.zip › plots/ferritin.png]

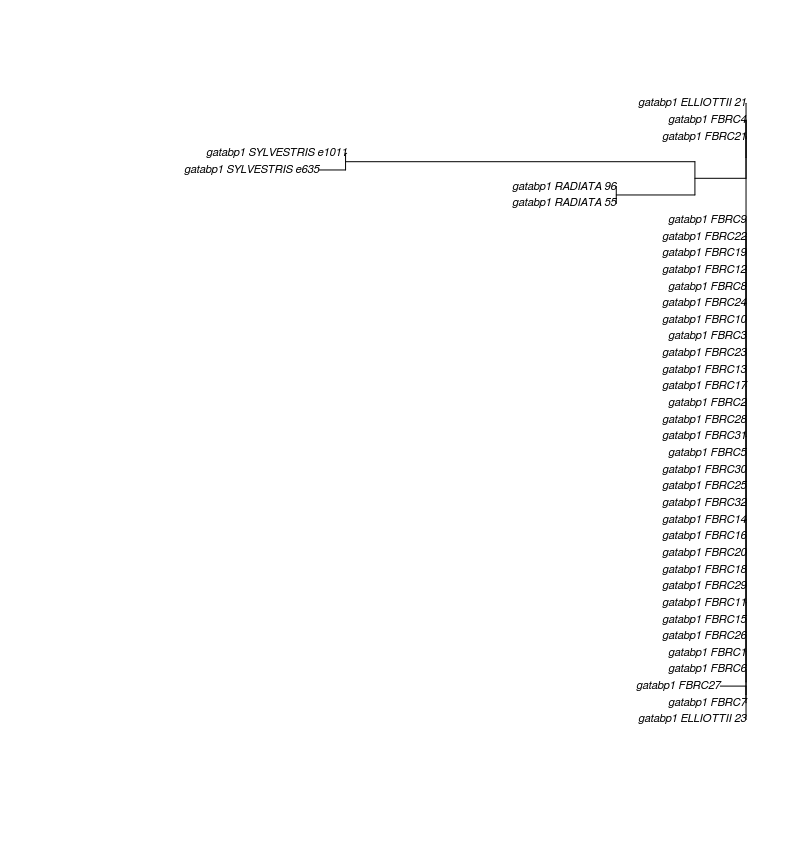

Supplement: Results S2 — NJ-tree plots for each loci. (1.70 MB ZIP) [file pone.0014234.s002.zip › plots/gatabp1.png]

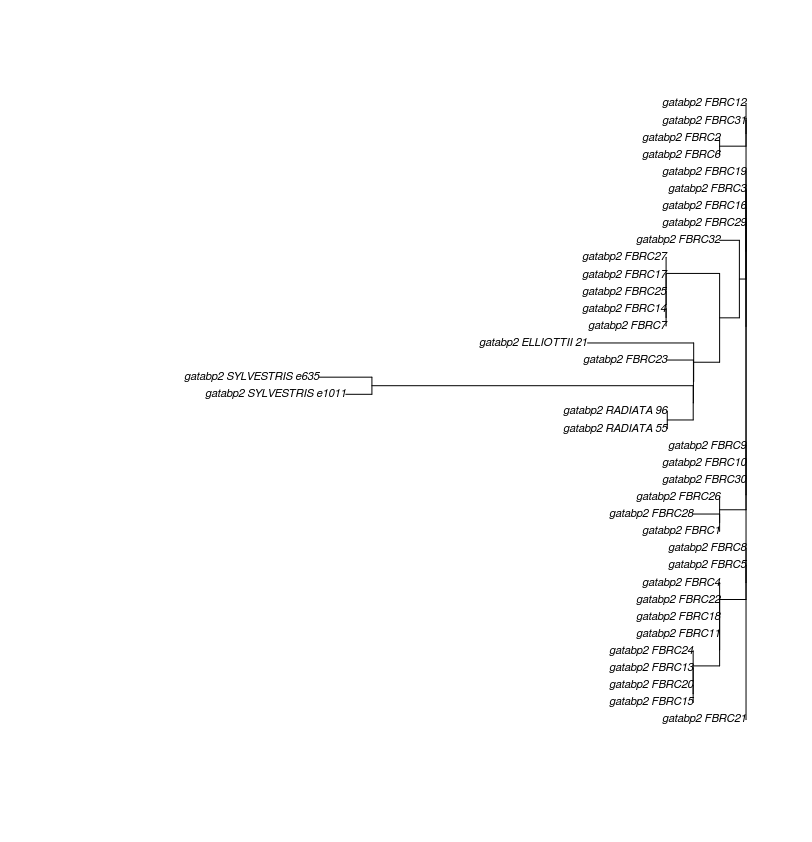

Supplement: Results S2 — NJ-tree plots for each loci. (1.70 MB ZIP) [file pone.0014234.s002.zip › plots/gatabp2.png]

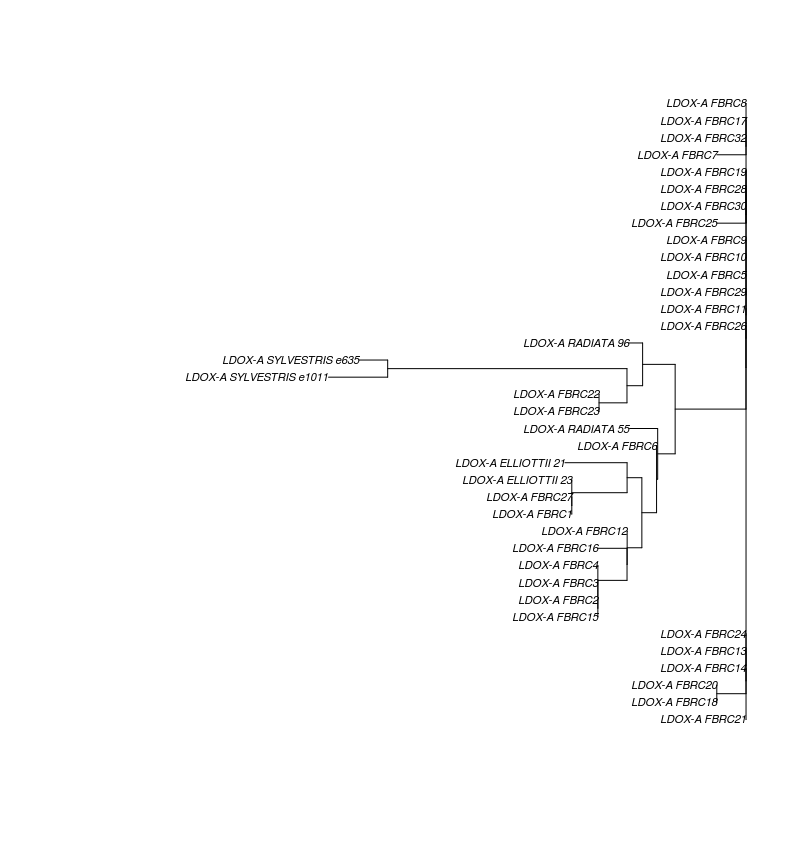

Supplement: Results S2 — NJ-tree plots for each loci. (1.70 MB ZIP) [file pone.0014234.s002.zip › plots/ldox-a.png]

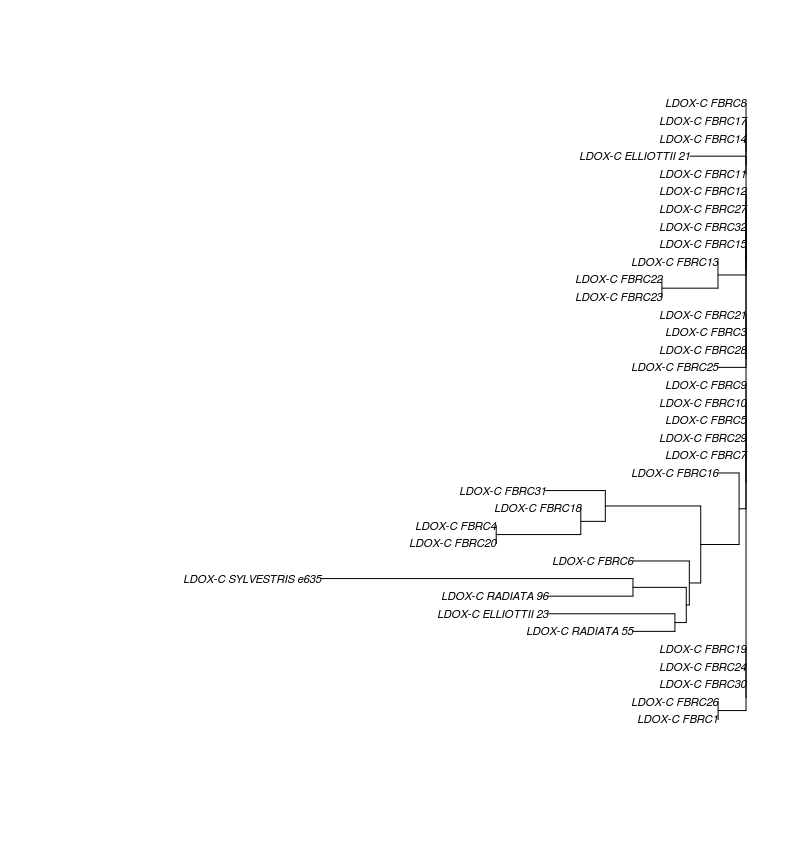

Supplement: Results S2 — NJ-tree plots for each loci. (1.70 MB ZIP) [file pone.0014234.s002.zip › plots/ldox-c.png]

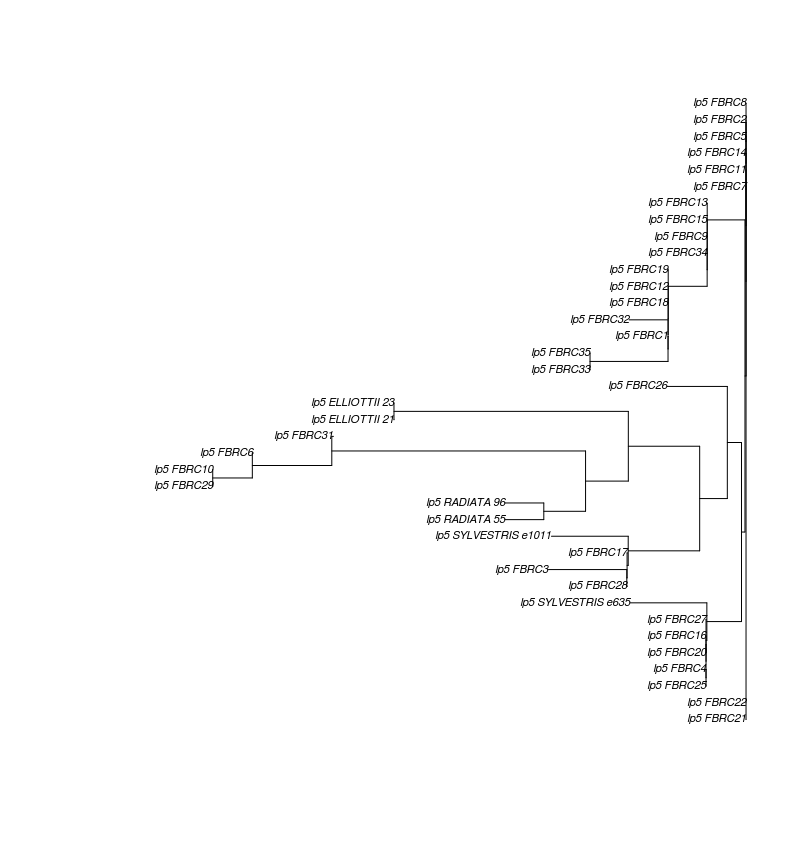

Supplement: Results S2 — NJ-tree plots for each loci. (1.70 MB ZIP) [file pone.0014234.s002.zip › plots/lp5.png]

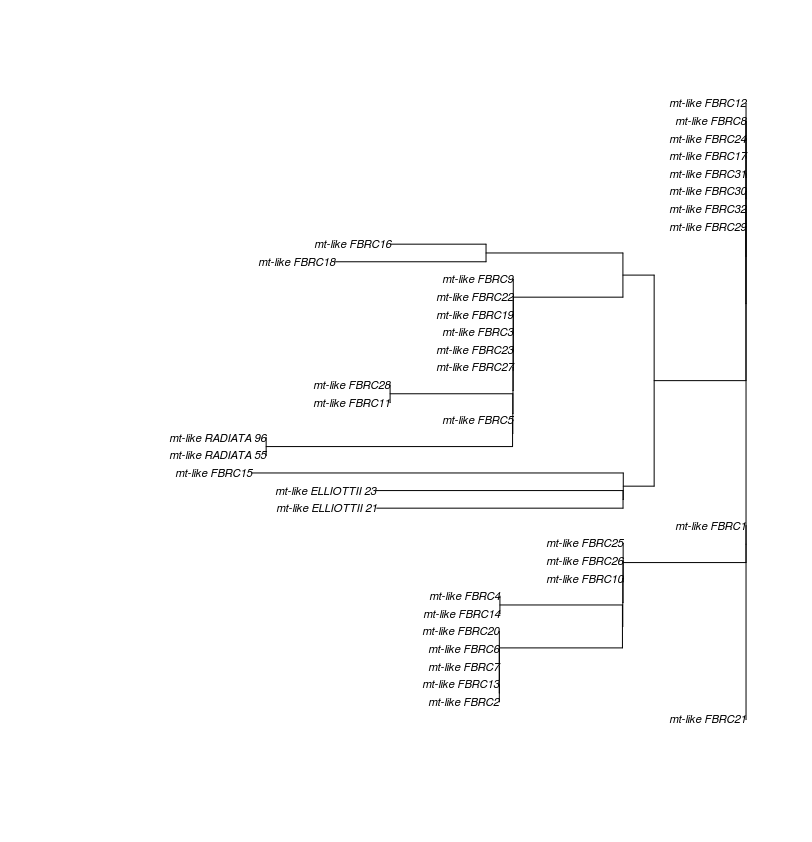

Supplement: Results S2 — NJ-tree plots for each loci. (1.70 MB ZIP) [file pone.0014234.s002.zip › plots/mtl-like.png]

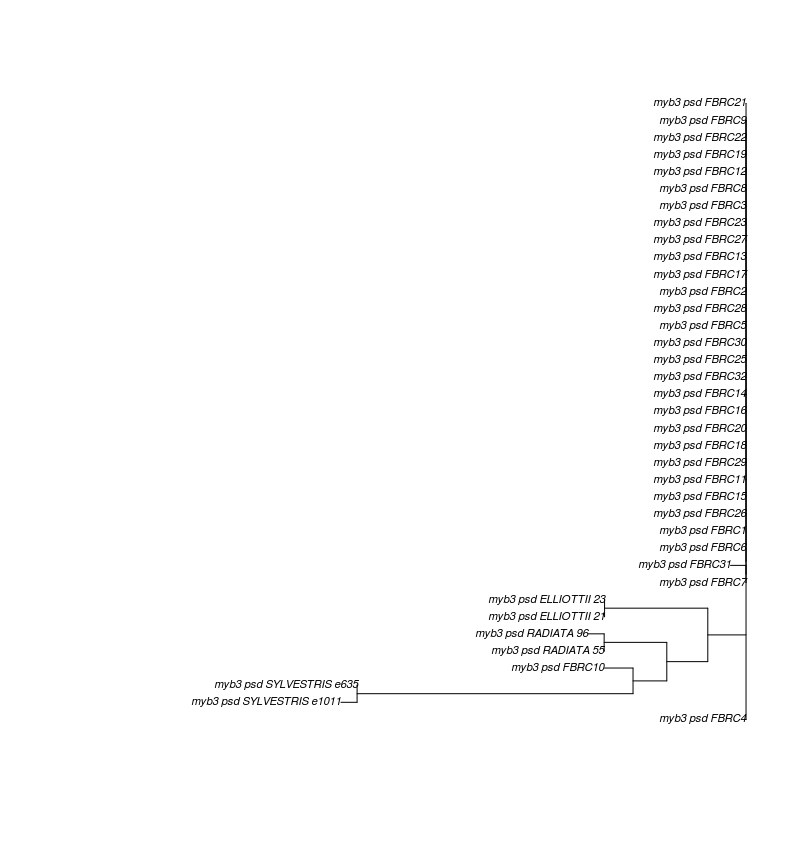

Supplement: Results S2 — NJ-tree plots for each loci. (1.70 MB ZIP) [file pone.0014234.s002.zip › plots/myb3.png]

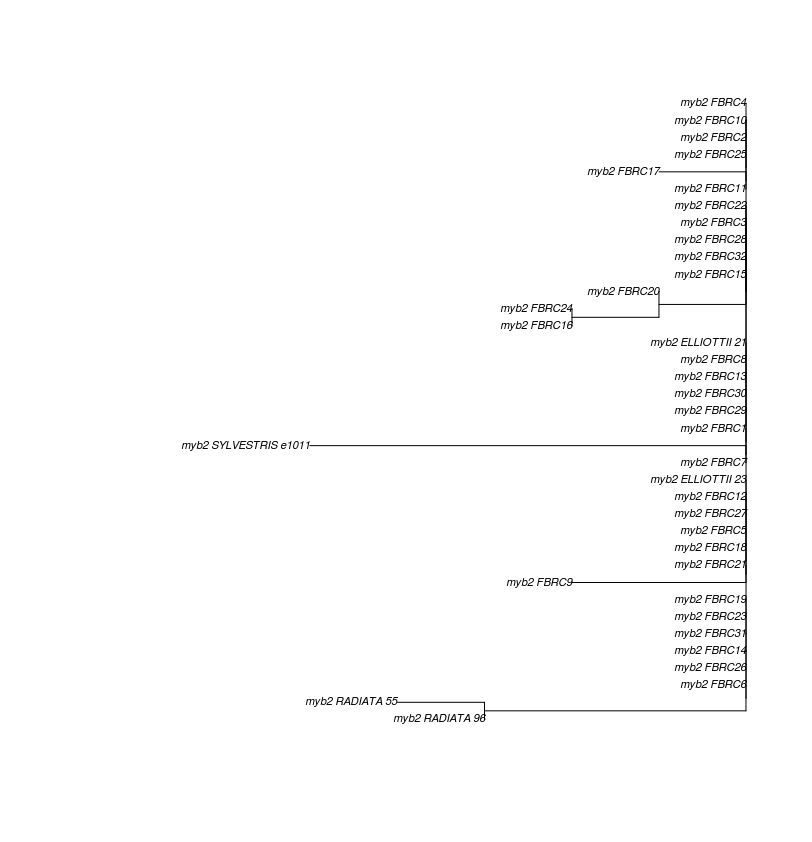

Supplement: Results S2 — NJ-tree plots for each loci. (1.70 MB ZIP) [file pone.0014234.s002.zip › plots/mybs3-like.png]

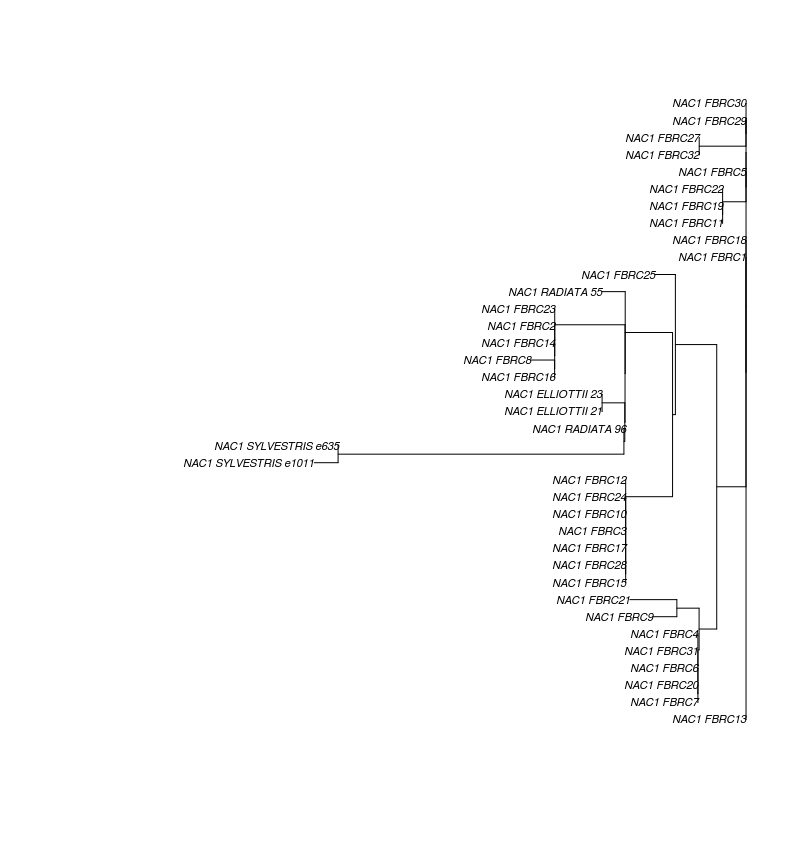

Supplement: Results S2 — NJ-tree plots for each loci. (1.70 MB ZIP) [file pone.0014234.s002.zip › plots/NAC1.png]

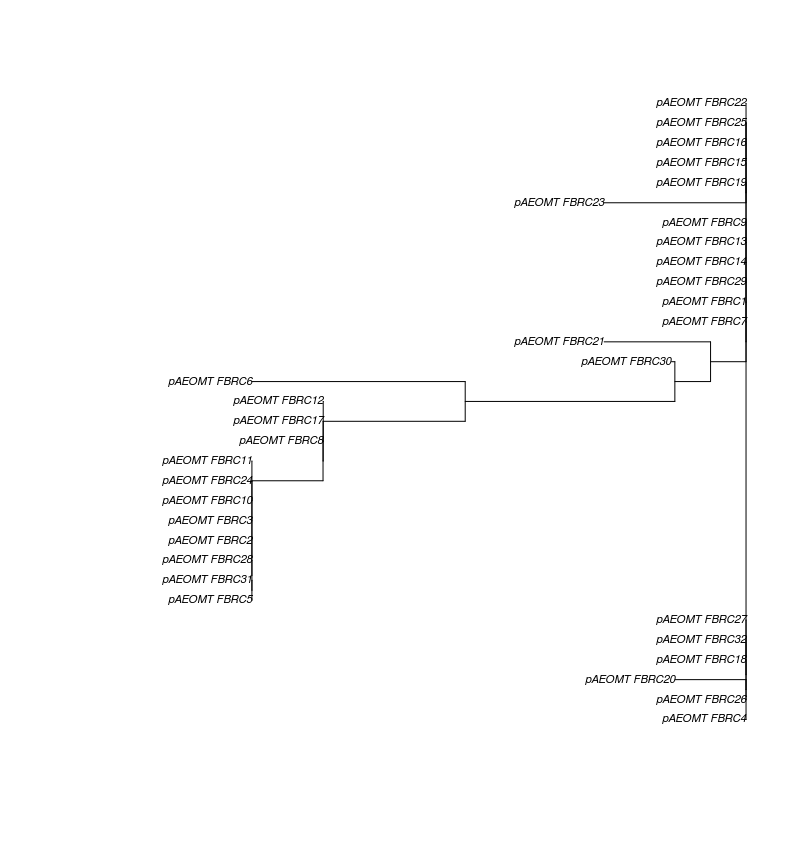

Supplement: Results S2 — NJ-tree plots for each loci. (1.70 MB ZIP) [file pone.0014234.s002.zip › plots/paeomt.png]

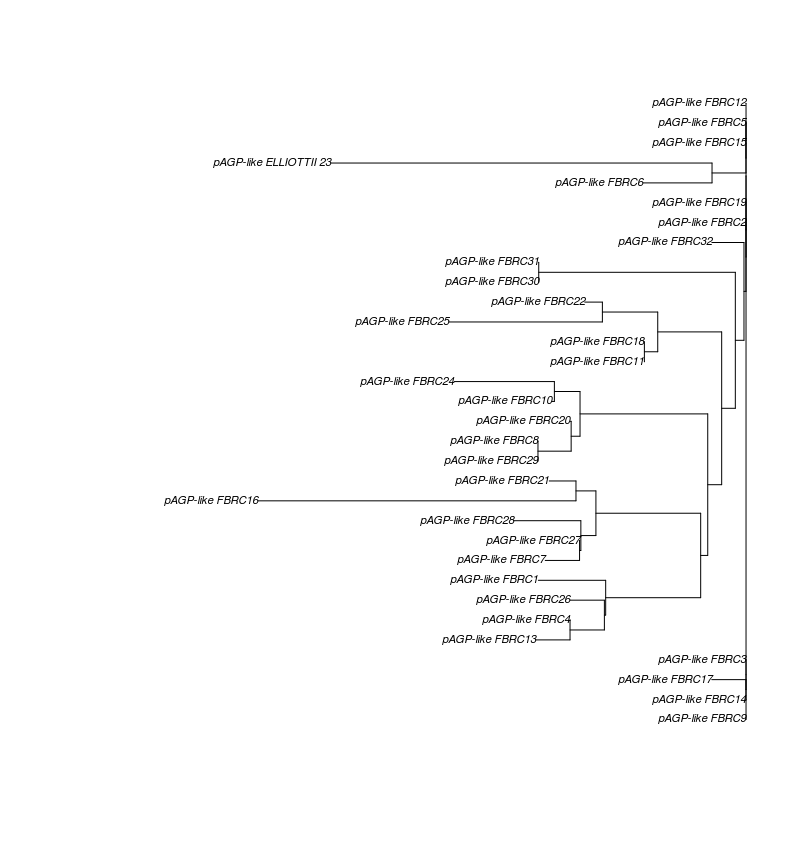

Supplement: Results S2 — NJ-tree plots for each loci. (1.70 MB ZIP) [file pone.0014234.s002.zip › plots/pAGP-like.png]

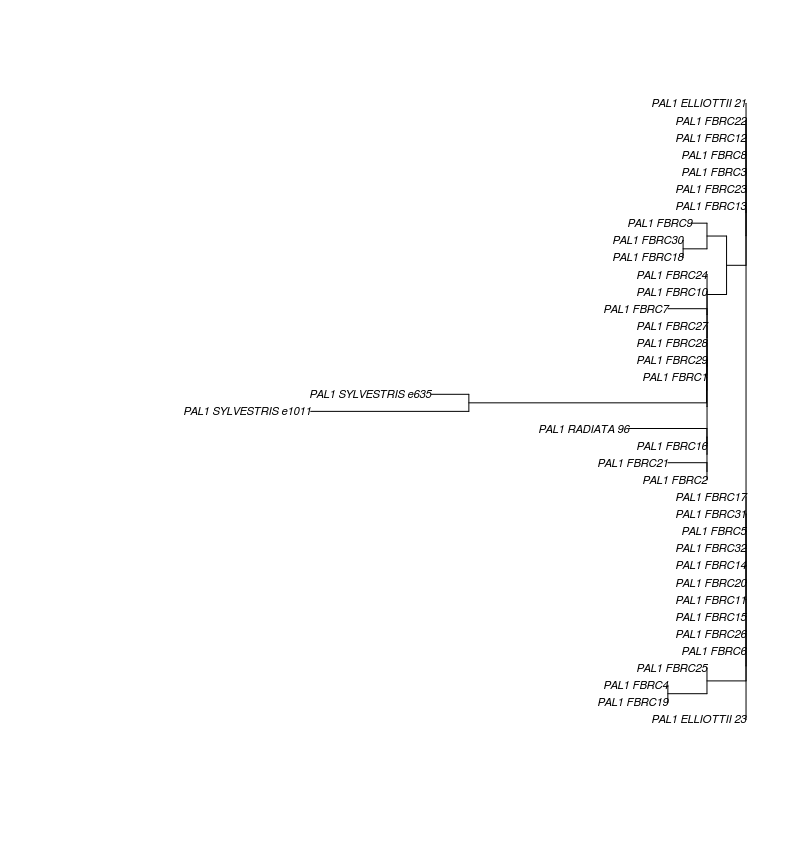

Supplement: Results S2 — NJ-tree plots for each loci. (1.70 MB ZIP) [file pone.0014234.s002.zip › plots/pal1.png]

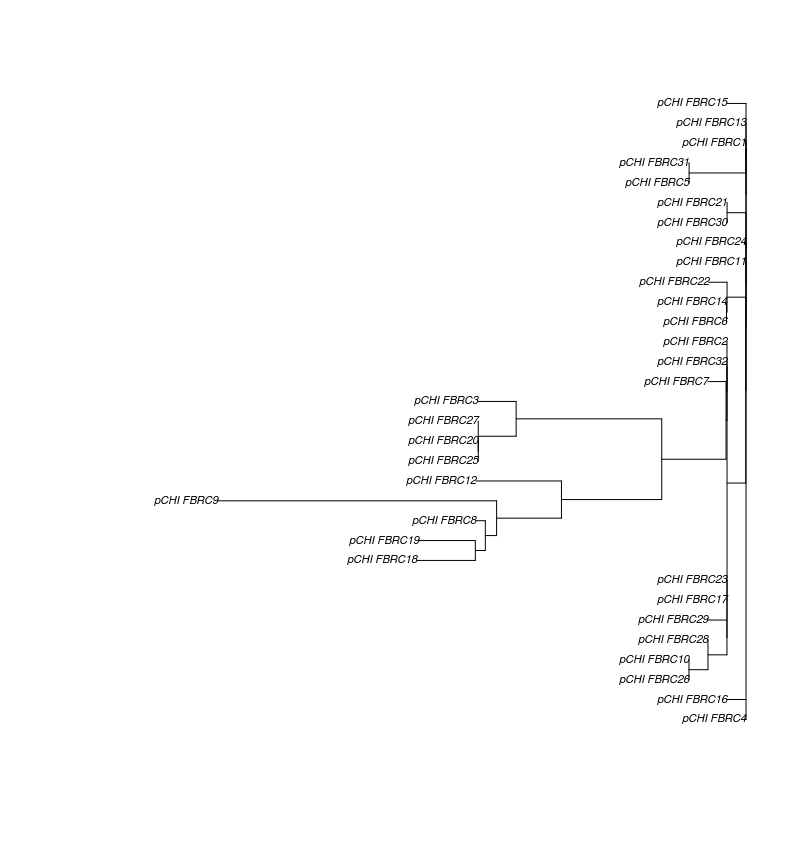

Supplement: Results S2 — NJ-tree plots for each loci. (1.70 MB ZIP) [file pone.0014234.s002.zip › plots/pCHI.png]

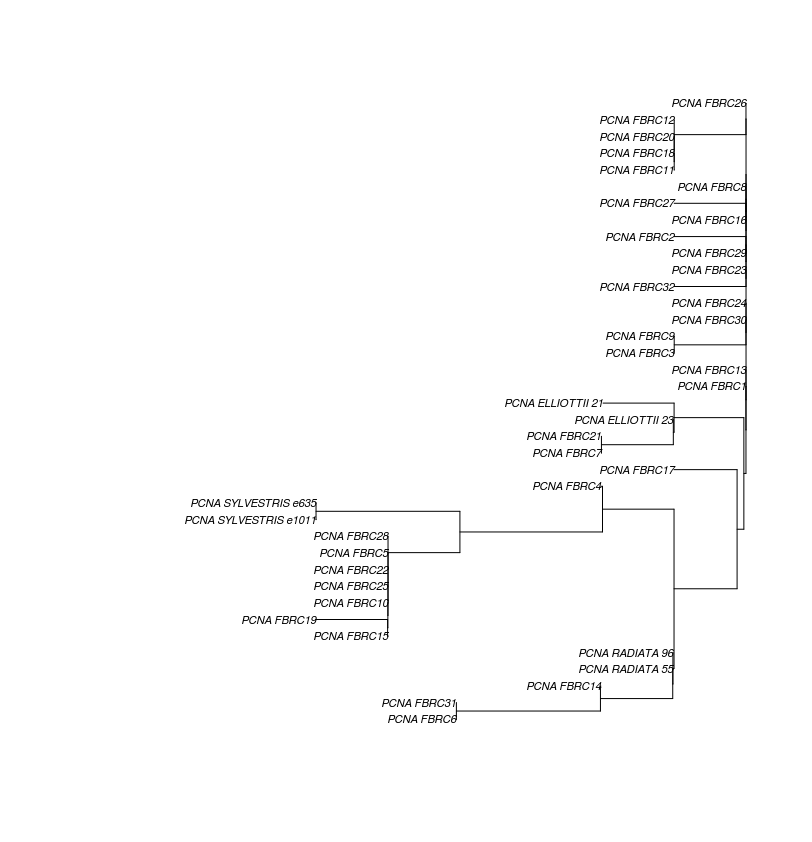

Supplement: Results S2 — NJ-tree plots for each loci. (1.70 MB ZIP) [file pone.0014234.s002.zip › plots/pcna.png]

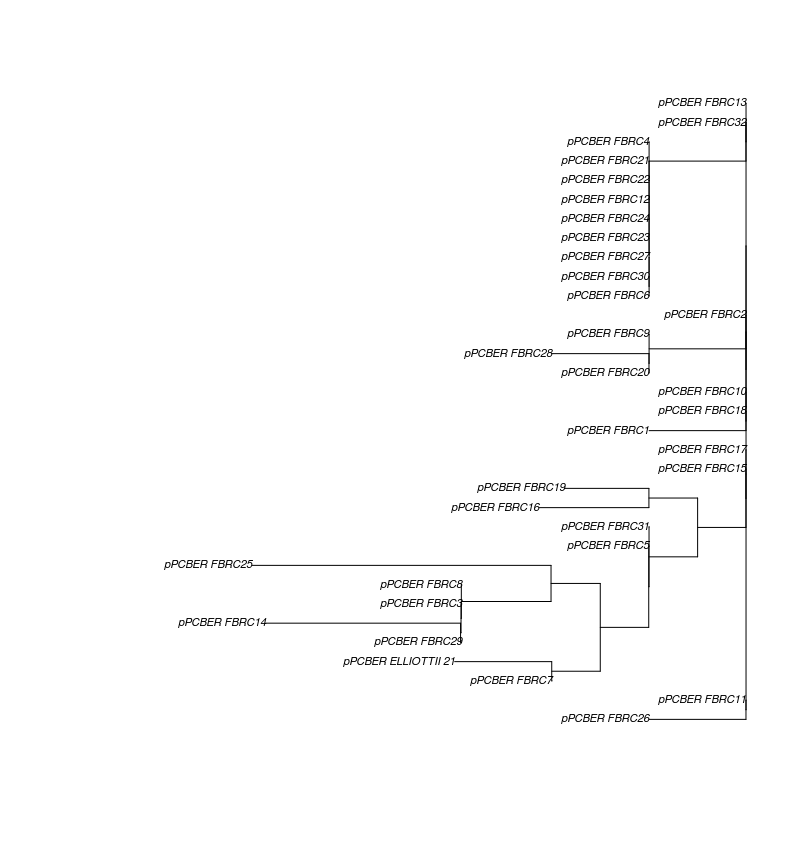

Supplement: Results S2 — NJ-tree plots for each loci. (1.70 MB ZIP) [file pone.0014234.s002.zip › plots/ppcber.png]

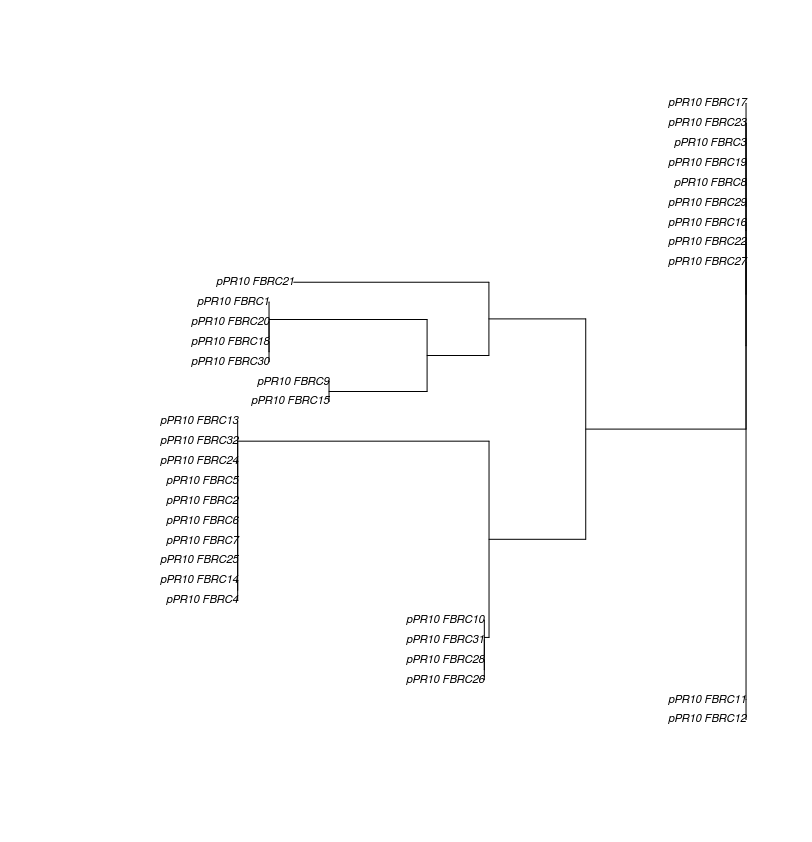

Supplement: Results S2 — NJ-tree plots for each loci. (1.70 MB ZIP) [file pone.0014234.s002.zip › plots/pr10.png]

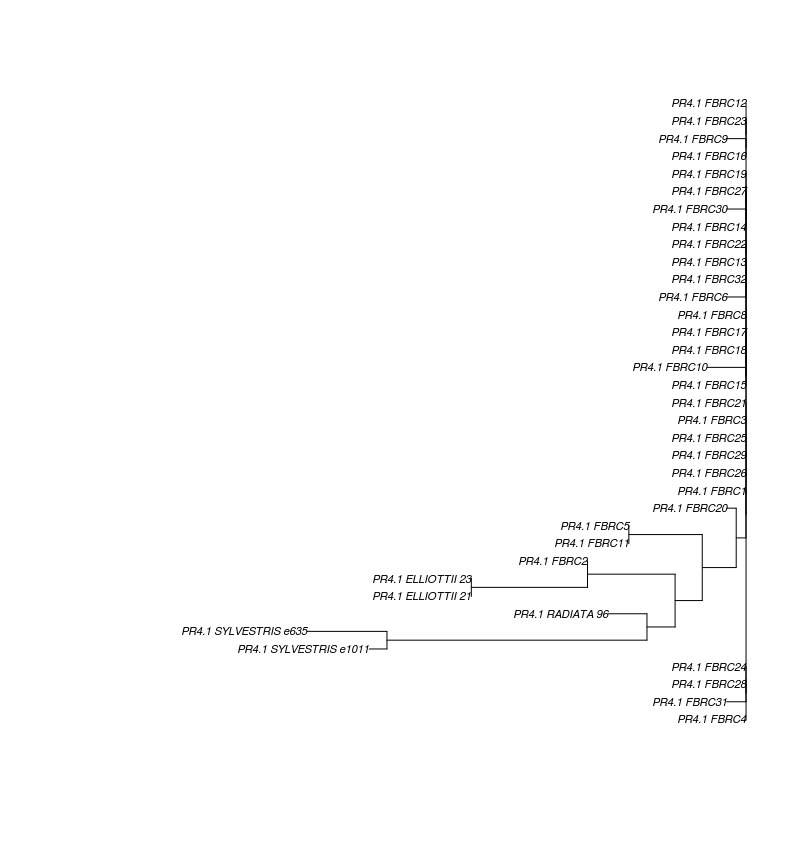

Supplement: Results S2 — NJ-tree plots for each loci. (1.70 MB ZIP) [file pone.0014234.s002.zip › plots/pr41.png]

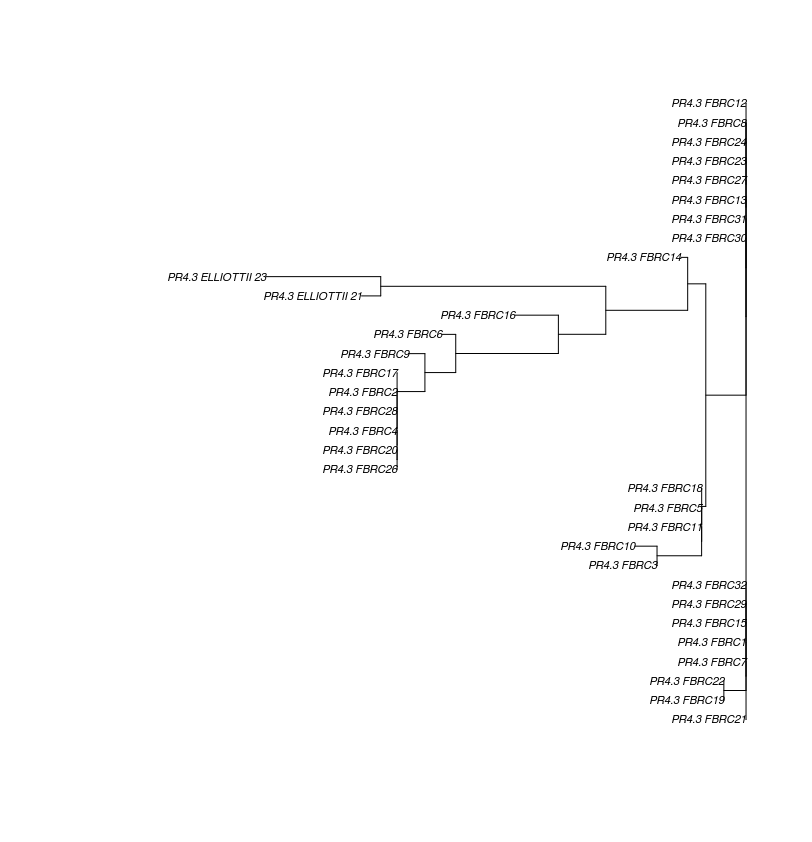

Supplement: Results S2 — NJ-tree plots for each loci. (1.70 MB ZIP) [file pone.0014234.s002.zip › plots/pr43.png]

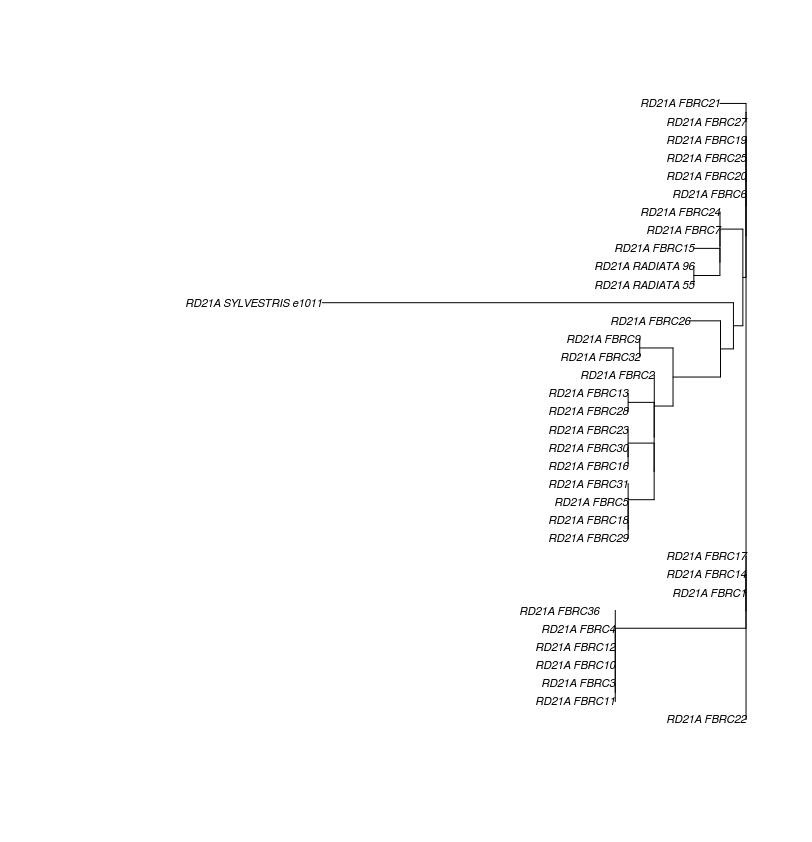

Supplement: Results S2 — NJ-tree plots for each loci. (1.70 MB ZIP) [file pone.0014234.s002.zip › plots/rd21a.png]

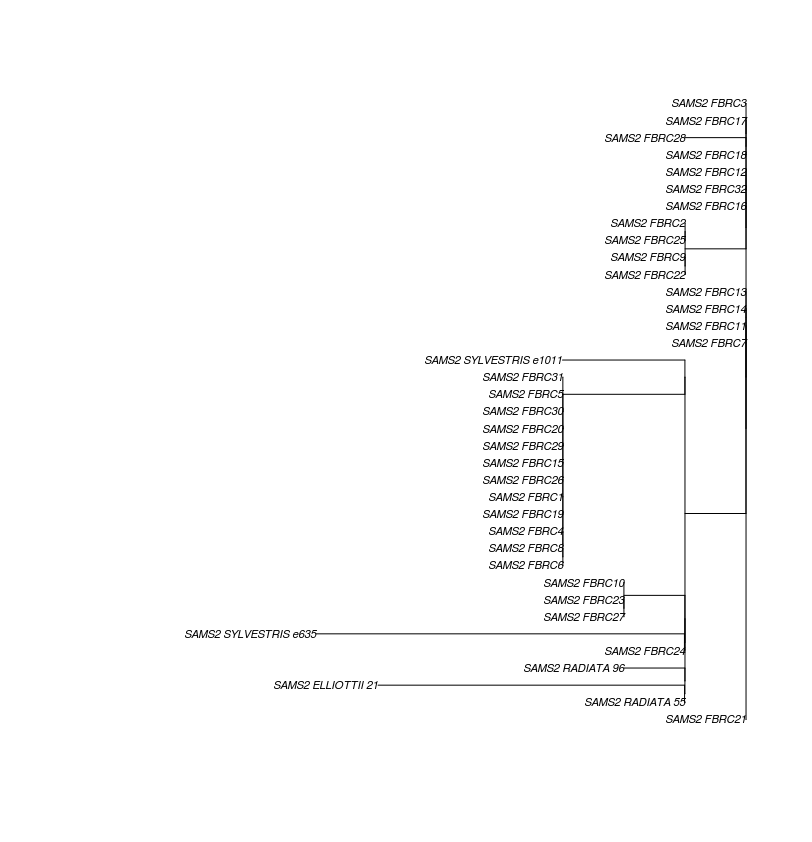

Supplement: Results S2 — NJ-tree plots for each loci. (1.70 MB ZIP) [file pone.0014234.s002.zip › plots/sams2.png]

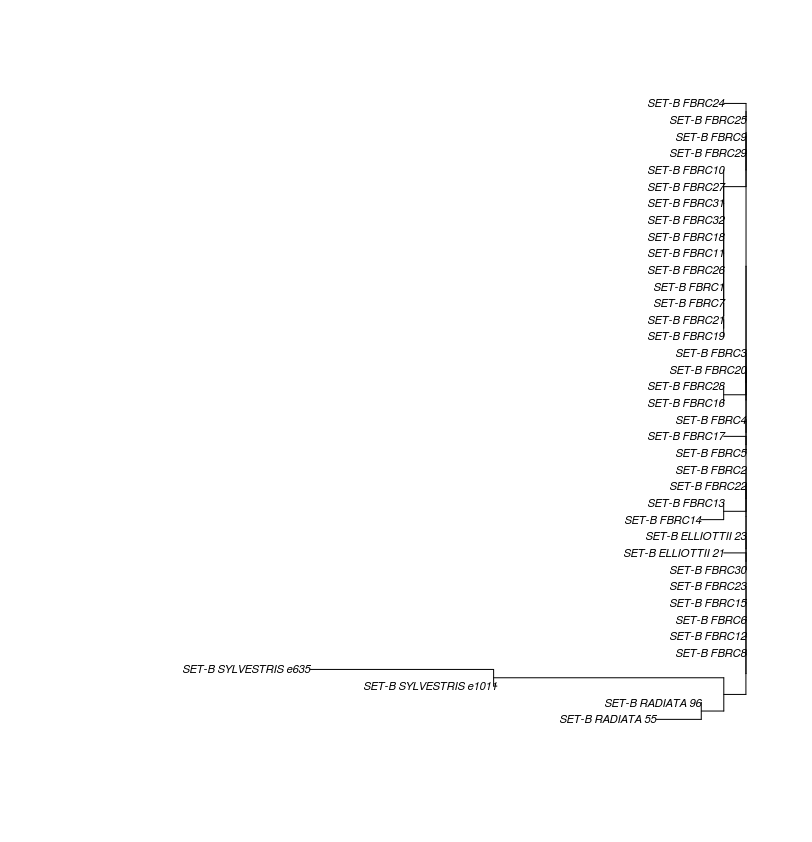

Supplement: Results S2 — NJ-tree plots for each loci. (1.70 MB ZIP) [file pone.0014234.s002.zip › plots/setb-like.png]

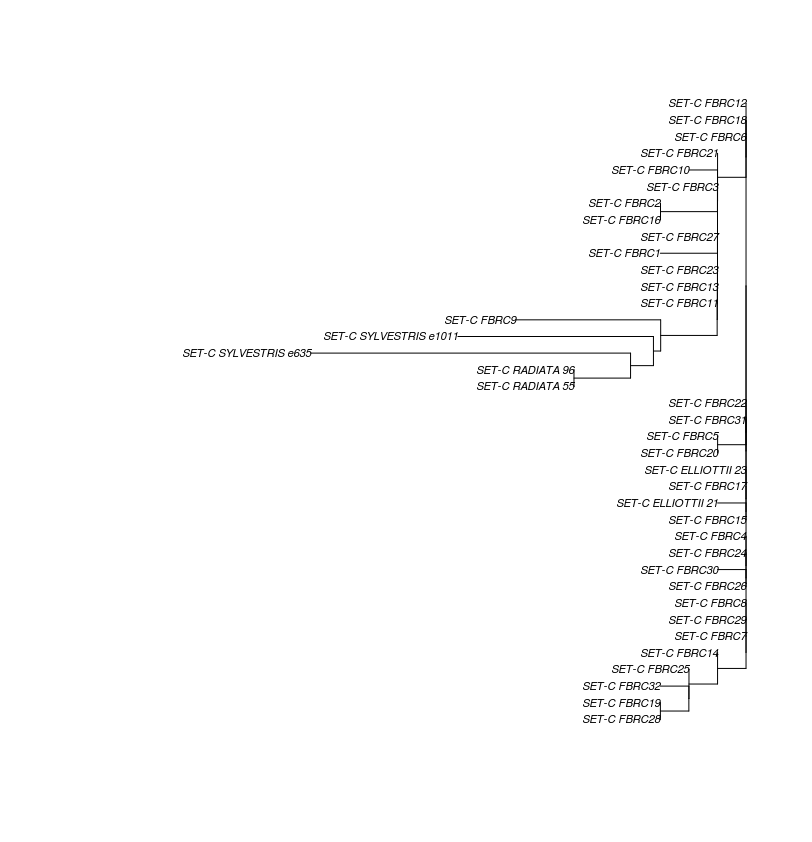

Supplement: Results S2 — NJ-tree plots for each loci. (1.70 MB ZIP) [file pone.0014234.s002.zip › plots/setc-like.png]

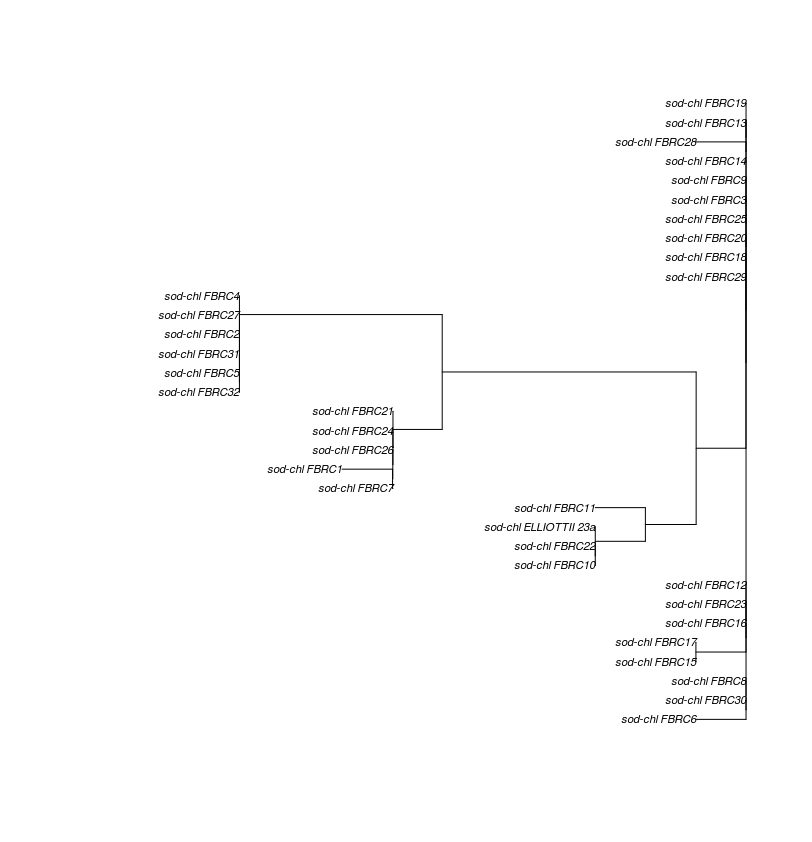

Supplement: Results S2 — NJ-tree plots for each loci. (1.70 MB ZIP) [file pone.0014234.s002.zip › plots/sod-chl.png]

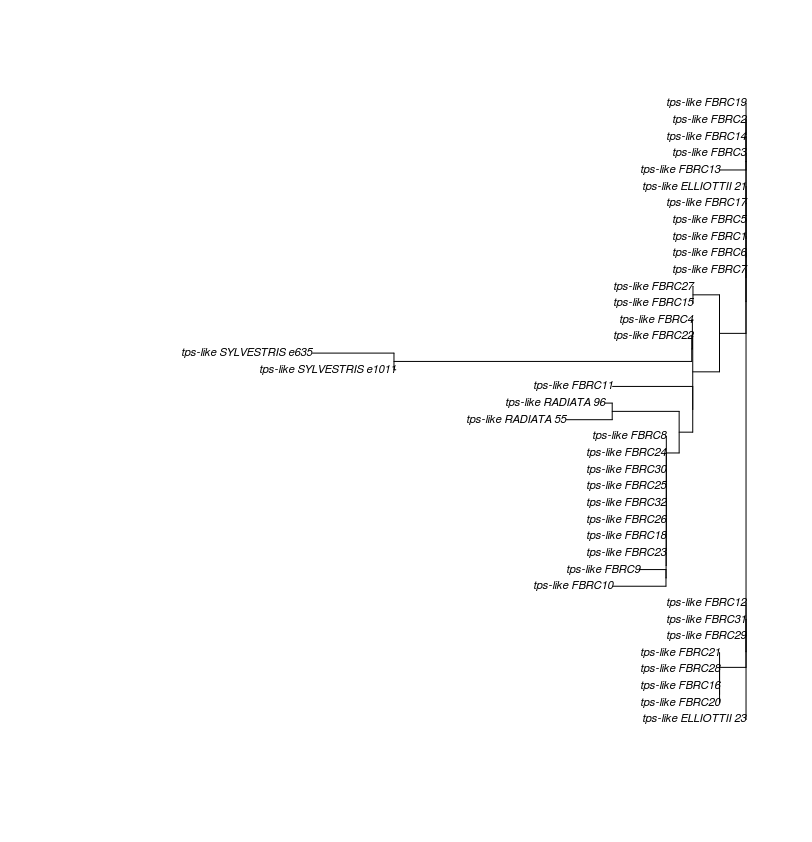

Supplement: Results S2 — NJ-tree plots for each loci. (1.70 MB ZIP) [file pone.0014234.s002.zip › plots/tps-like.png]

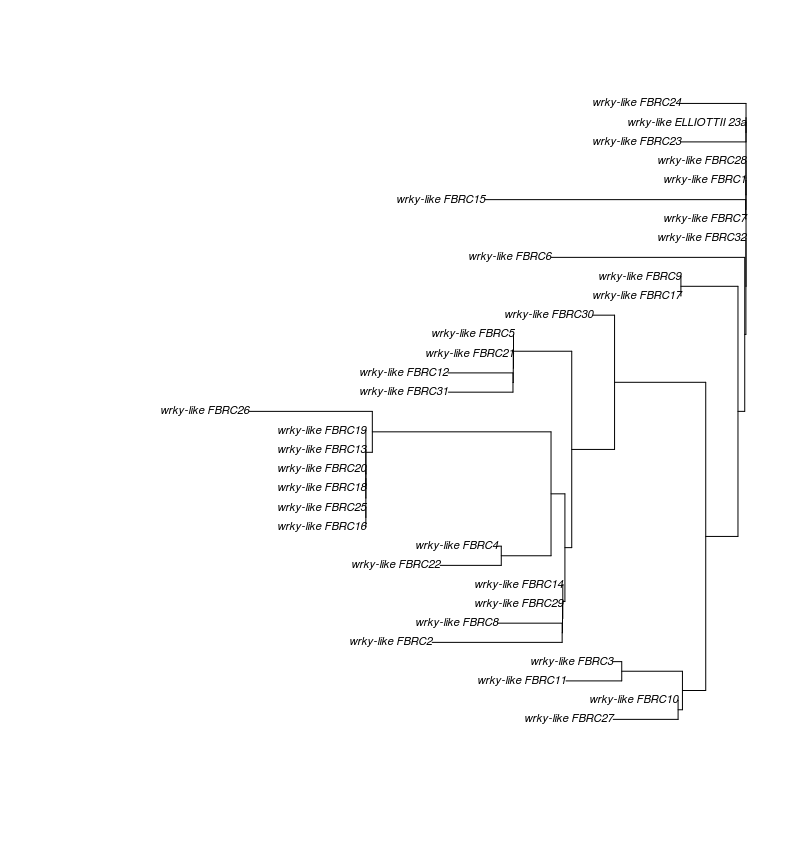

Supplement: Results S2 — NJ-tree plots for each loci. (1.70 MB ZIP) [file pone.0014234.s002.zip › plots/wrky-like2.png]

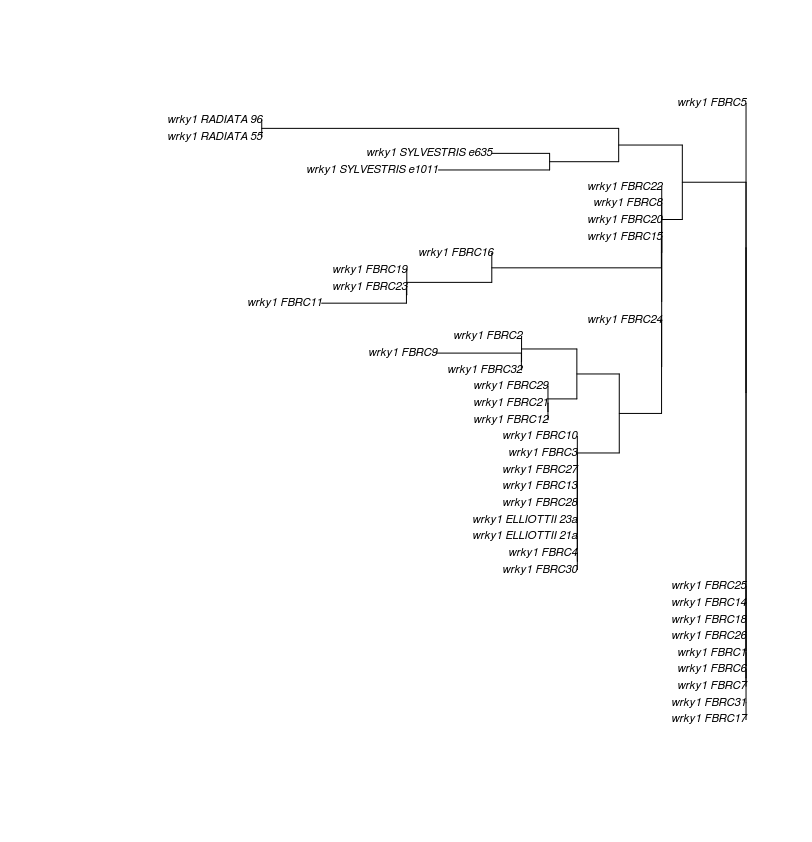

Supplement: Results S2 — NJ-tree plots for each loci. (1.70 MB ZIP) [file pone.0014234.s002.zip › plots/wrky1-like.png]
