# Supplementary material for: Evolution of Disease Response Genes in Loblolly Pine: Insights from Candidate Genes
Source: PLoS One. 2010 Dec 6;5(12):e14234. doi: 10.1371/journal.pone.0014234 (PMC2997792; doi:10.1371/journal.pone.0014234)
Supplement: References S1 — References associating candidate genes with disease resistance phenotypes. (0.04 MB DOC) [file pone.0014234.s003.doc]

**References S1.** References associating candidates with disease resistance

1. Ton J, De Vos M, Robben C, Buchala A, Metraux JP, et al. (2002) Characterization of Arabidopsis enhanced disease susceptibility mutants that are affected in systemically induced resistance. Plant J 29: 11-21.

2. Zhang Y, Brown G, Whetten R, Loopstra CA, Neale D, et al. (2003) An arabinogalactan protein associated with secondary cell wall formation in differentiating xylem of loblolly pine. Plant Mol Biol 52: 91-102.

3. Singh J, Roberts MR (2004) Fusicoccin activates pathogen-responsive gene expression independently of common resistance signalling pathways, but increases disease symptoms in Pseudomonas syringae-infected tomato plants. Planta 219: 261-269.

4. Kaya H, Shibahara KI, Taoka KI, Iwabuchi M, Stillman B, et al. (2001) FASCIATA genes for chromatin assembly factor-1 in arabidopsis maintain the cellular organization of apical meristems. Cell 104: 131-142.

5. Castillo AG, Collinet D, Deret S, Kashoggi A, Bejarano ER (2003) Dual interaction of plant PCNA with geminivirus replication accessory protein (Ren) and viral replication protein (Rep). Virology 312: 381-394.

6. Geraats BP, Bakker PA, van Loon LC (2002) Ethylene insensitivity impairs resistance to soilborne pathogens in tobacco and Arabidopsis thaliana. Mol Plant Microbe Interact 15: 1078-1085.

7. Pritsch C, Muehlbauer GJ, Bushnell WR, Somers DA, Vance CP (2000) Fungal development and induction of defense response genes during early infection of wheat spikes by Fusarium graminearum. Mol Plant Microbe Interact 13: 159-169.

8. Pikaard CS (1999) Nucleolar dominance and silencing of transcription. Trends Plant Sci 4: 478-483.

9. Niggeweg R, Thurow C, Kegler C, Gatz C (2000) Tobacco transcription factor TGA2.2 is the main component of as-1-binding factor ASF-1 and is involved in salicylic acid- and auxin-inducible expression of as-1-containing target promoters. J Biol Chem 275: 19897-19905.

10. Schulze-Lefert P, Dangl JL, Becker-Andre M, Hahlbrock K, Schulz W (1989) Inducible in vivo DNA footprints define sequences necessary for UV light activation of the parsley chalcone synthase gene. Embo J 8: 651-656.

11. Burlat V, Kwon M, Davin LB, Lewis NG (2001) Dirigent proteins and dirigent sites in lignifying tissues. Phytochemistry 57: 883-897.

12. Wang YH, Choi W, Thomas CE, Dean RA (2002) Cloning of disease-resistance homologues in end sequences of BAC clones linked to Fom-2, a gene conferring resistance to Fusarium wilt in melon (Cucumis melo L.). Genome 45: 473-480.

13. Ellard-Ivey M, Douglas CJ (1996) Role of Jasmonates in the Elicitor- and Wound-Inducible Expression of Defense Genes in Parsley and Transgenic Tobacco. Plant Physiol 112: 183-192.

14. Franke R, Hemm MR, Denault JW, Ruegger MO, Humphreys JM, et al. (2002) Changes in secondary metabolism and deposition of an unusual lignin in the ref8 mutant of Arabidopsis. Plant J 30: 47-59.

15. Narusaka Y, Narusaka M, Seki M, Umezawa T, Ishida J, et al. (2004) Crosstalk in the responses to abiotic and biotic stresses in Arabidopsis: analysis of gene expression in cytochrome P450 gene superfamily by cDNA microarray. Plant Mol Biol 55: 327-342.

16. Gu YQ, Wildermuth MC, Chakravarthy S, Loh YT, Yang C, et al. (2002) Tomato transcription factors pti4, pti5, and pti6 activate defense responses when expressed in Arabidopsis. Plant Cell 14: 817-831.

17. Guo HS, Xie, Q., Fei, J.F. Chua, N.H. (2005) MicroRNA directs mRNA cleavage of the transcription factor NAC1 to downregulate auxin signals for arabidopsis lateral root development. Plant Cell 17(5): 1376-1386.

18. Mengiste T, Chen X, Salmeron J, Dietrich R (2003) The BOTRYTIS SUSCEPTIBLE1 gene encodes an R2R3MYB transcription factor protein that is required for biotic and abiotic stress responses in Arabidopsis. Plant Cell 15: 2551-2565.

19. Yu D, Chen C, Chen Z (2001) Evidence for an important role of WRKY DNA binding proteins in the regulation of NPR1 gene expression. Plant Cell 13: 1527-1540.

20. Hartmann U, Sagasser M, Mehrtens F, Stracke R, Weisshaar B (2005) Differential combinatorial interactions of cis-acting elements recognized by R2R3-MYB, BZIP, and BHLH factors control light-responsive and tissue-specific activation of phenylpropanoid biosynthesis genes. Plant Mol Biol 57: 155-171.

21. Avonce N, Leyman B, Mascorro-Gallardo JO, Van Dijck P, Thevelein JM, et al. (2004) The Arabidopsis trehalose-6-P synthase AtTPS1 gene is a regulator of glucose, abscisic acid, and stress signaling. Plant Physiol 136: 3649-3659.

22. Kim H, Ralph J, Lu F, Ralph SA, Boudet AM, et al. (2003) NMR analysis of lignins in CAD-deficient plants. Part 1. Incorporation of hydroxycinnamaldehydes and hydroxybenzaldehydes into lignins. Org Biomol Chem 1: 268-281.

23. Martin D, Tholl D, Gershenzon J, Bohlmann J (2002) Methyl jasmonate induces traumatic resin ducts, terpenoid resin biosynthesis, and terpenoid accumulation in developing xylem of Norway spruce stems. Plant Physiol 129: 1003-1018.

24. Thundathil J, de Lamirande E, Gagnon C (2002) Different signal transduction pathways are involved during human sperm capacitation induced by biological and pharmacological agents. Mol Hum Reprod 8: 811-816.

25. Chang S, Puryear J, Funkhouser EA, Newton RJ, Cairney J (1996) Cloning of a cDNA for a chitinase homologue which lacks chitin-binding sites and is down-regulated by water stress and wounding. Plant Mol Biol 31: 693-699.

26. Mason ME, Davis JM (1997) Defense response in slash pine: chitosan treatment alters the abundance of specific mRNAs. Mol Plant Microbe Interact 10: 135-137.

27. Pirttila AM, Laukkanen H, Hohtola A (2002) Chitinase production in pine callus (Pinus sylvestris L.): a defense reaction against endophytes? Planta 214: 848-852.

28. Rojas MG, Morales-Ramos JA (2001) Bait matrix for delivery of chitin synthesis inhibitors to the formosan subterranean termite (Isoptera: Rhinotermitidae). J Econ Entomol 94: 506-510.

29. Wu H, Echt CS, Popp MP, Davis JM (1997) Molecular cloning, structure and expression of an elicitor-inducible chitinase gene from pine trees. Plant Mol Biol 33: 979-987.

30. Davidson J, Ekramoddoullah AK (1997) Analysis of bark proteins in blister rust-resistant and susceptible western white pine (Pinus monticola). Tree Physiol 17: 663-669.

31. Dubos C, Plomion C (2001) Drought differentially affects expression of a PR-10 protein, in needles of maritime pine (Pinus pinaster Ait.) seedlings. J Exp Bot 52: 1143-1144.

32. Li GH, Shen YM, Zhang KQ (2005) Nematicidal activity and chemical component of Poria cocos. J Microbiol 43: 17-20.

33. Liu JJ, Ekramoddoullah AK (2004) Characterization, expression and evolution of two novel subfamilies of Pinus monticola cDNAs encoding pathogenesis-related (PR)-10 proteins. Tree Physiol 24: 1377-1385.

34. Liu X, Wei J, Tan F, Zhou S, Wurthwein G, et al. (2004) Pycnogenol, French maritime pine bark extract, improves endothelial function of hypertensive patients. Life Sci 74: 855-862.

35. Rensing KH, Samuels AL, Savidge RA (2002) Ultrastructure of vascular cambial cell cytokinesis in pine seedlings preserved by cryofixation and substitution. Protoplasma 220: 39-49.

36. Ro DK, Arimura G, Lau SY, Piers E, Bohlmann J (2005) Loblolly pine abietadienol/abietadienal oxidase PtAO (CYP720B1) is a multifunctional, multisubstrate cytochrome P450 monooxygenase. Proc Natl Acad Sci U S A 102: 8060-8065.

37. Syed Z, Guerin PM, Baltensweiler W (2003) Antennal responses of the two host races of the larch bud moth, Zeiraphera diniana, to larch and cembran pine volatiles. J Chem Ecol 29: 1691-1708.

38. Whetten R, Sun YH, Zhang Y, Sederoff R (2001) Functional genomics and cell wall biosynthesis in loblolly pine. Plant Mol Biol 47: 275-291.

39. Vance CP, Kirk TK, Sherwood RT. Lignification as a mechanism of disease resistance. Ann Review Phytopath. 1980;18:259–288

40. Nicholson RL, Hammerschmidt R. Phenolic compounds and their role in disease resistance. Ann Rev Phytopath. 1992;30:369–389

41. Hückelhoven R. Cell wall-associated mechanisms of disease resistance and susceptibility. Annu Rev Phytopathol. 2007;45:1–27
