# Supplementary material for: Evolution of Disease Response Genes in Loblolly Pine: Insights from Candidate Genes
Source: PLoS One. 2010 Dec 6;5(12):e14234. doi: 10.1371/journal.pone.0014234 (PMC2997792; doi:10.1371/journal.pone.0014234)
Supplement: Table S1 — Theoretical expectations under the tests performed and the conclusions on selection patterns according to the test results presented in this manuscript. (0.03 MB DOC) [file pone.0014234.s004.doc]

**Table S1*.***  *Theoretical expectations under the tests performed and the conclusions on selection patterns according to the test results presented in this manuscript.*

| **Type of selection** | **Description** | **Expectations** | **SFS** | **MKPRF** | **IM** | **Disease Response Hypothesis** |
| --- | --- | --- | --- | --- | --- | --- |
| **Negative or purifying selection** | mutations generate deleterious alleles which are selected against | reduced amino acid replacements, excess of rare alleles | no rejection, or reject neutrality with excess of rare alleles | γ< 0 | no rejection or reject with excess polymorphisms fixed between species(S_F_) | not involved in host-pathogen interaction |
| **Selective sweep** | selection strongly favoring a recent new allele | reduced overall diversity, with relative excess of low frequency or high frequency derived alleles; amino acid replacement rate unaffected | reject neutrality; excess rare alleles, low diversity | γ ~ 0 | no rejection or reject with excess polymorphisms specific to species 1(S_x1_) | arms race |
| **Directional selection** | repeated selective fixations of new alleles | increased rate of amino acid replacements | no rejection, or reject as in selective sweep | γ > 0 | no rejection or reject with excess polymorphism fixed between species(S_F_) | arms race |
| **Balancing selection** | selection favoring maintenance of existing alleles, preventing fixation | reduced levels of divergence, shared polymorphisms with out-groups, excess of mid frequency alleles | reject neutrality; excess of mid frequency alleles | γ < 0, γ ~ 0 | reject with excess shared polymorphisms between species(S_S_) | trench warfare |
| **Diversifying selection** | selection favoring the maintenance of several alleles at a locus, but not necessarily preventing fixation of any one mutation or selecting against new mutations | excess of mid frequency alleles, increased diversity | reject neutrality; excess of mid frequency alleles | γ ~ 0, γ > 0 | no rejection or reject with excess polymorphisms specific to species 1(S_x1_) | trench warfare |
