# Supplementary material for: Evolution of Disease Response Genes in Loblolly Pine: Insights from Candidate Genes
Source: PLoS One. 2010 Dec 6;5(12):e14234. doi: 10.1371/journal.pone.0014234 (PMC2997792; doi:10.1371/journal.pone.0014234)
Supplement: Table S2 — Selections and their origins Geographical origin of trees and relatedness in second generation crosses. Isolate IDs are provided as descriptors at GenBank accession numbers. For second generation trees we give between parentheses the ID of their parental trees, when parents were included in the sample as first selections. NA: data not available. (0.05 MB DOC) [file pone.0014234.s005.doc]

**Table S2-** Selections and their origins Geographical origin of trees and relatedness in second generation crosses. Isolate IDs are provided as descriptors at GenBank accession numbers. For second generation trees we give between parentheses the ID of their parental trees, when parents were included in the sample as first selections. NA: data not available.

| **Isolate ID** *^a^* | **Geographic origins of trees** | | | | | **Provenance** |
| --- | --- | --- | --- | --- | --- | --- |
|  | **County** | **State** |  | **County** | **State** |  |
| FBRC1 | Effingham | GA | X | NA | NA | Atlantic Coastal Plain |
| FBRC2 | Marion | FL | First generation selection | | | Marion County Florida |
| FBRC3 | Nassau | FL | First generation selection | | | Northern Florida |
| FBRC4 | Williamsburg | SC | First generation selection | | | Atlantic Coastal Plain |
| FBRC5 | Marion | FL | First generation selection | | | Marion County Florida |
| FBRC6 | Effingham | GA | First generation selection | | | Atlantic Coastal Plain |
| FBRC7 | Jasper | SC | First generation selection | | | Atlantic Coastal Plain |
| FBRC8 | Georgetown | SC | First generation selection | | | Atlantic Coastal Plain |
| FBRC9 | Georgetown | SC | X | Brunswick | NC | Atlantic Coastal Plain |
| FBRC10 | Choctaw | AL | First generation selection | | | Gulf Coast |
| FBRC11 | Georgetown | SC | First generation selection | | | Atlantic Coastal Plain |
| FBRC12 | Georgetown | SC | First generation selection | | | Atlantic Coastal Plain |
| FBRC13 | Marion | FL | First generation selection | | | Marion County Florida |
| FBRC14 | Levy | FL | First generation selection | | | Central Florida |
| FBRC15 * | Jasper | SC | X | Chatham | GA | Atlantic Coastal Plain |
| FBRC16 | Levy | FL | First generation selection | | | Central Florida |
| FBRC17 | Marion | FL | First generation selection | | | Marion County Florida |
| FBRC18 † | Georgetown | SC | X | Georgetown | SC | Atlantic Coastal Plain |
| FBRC19 ‡ | Horry | SC | X | Georgetown (FBRC8) | SC | Atlantic Coastal Plain |
| FBRC20 § | Williamsburg (FBRC4) | SC | X | Georgetown (FBRC11) | SC | Atlantic Coastal Plain |
| FBRC21 | Georgetown | SC | First generation selection | | | Atlantic Coastal Plain |
| FBRC22 ‡§ | Williamsburg (FBRC4) | SC | X | Horry | SC | Atlantic Coastal Plain |
| FBRC23 | Nassau | FL | First generation selection | | | Northern Florida |
| FBRC24 | Franklin | MS | First generation selection | | | Gulf Coast |
| FBRC25 | Levy | FL | First generation selection | | | Central Florida |
| FBRC26 * | Liberty | GA | X | Chatham | GA | Atlantic Coastal Plain |
| FBRC27 | Wilkinson | MS | First generation selection | | | Gulf Coast |
| FBRC28 | Marion | FL | First selection | | | Marion County Florida |
| FBRC29 † | Georgetown | SC | X | Georgetown | SC | Atlantic Coastal Plain |
| FBRC30 | Marion | FL | First generation selection | | | Marion County Florida |
| FBRC31 | Marion | FL | First generation selection | | | Marion County Florida |
| FBRC32 | Levy | FL | First generation selection | | | Central Florida |

*^a^* Trees with the same symbol are half-sibs
