# Supplementary material for: Evolution of Disease Response Genes in Loblolly Pine: Insights from Candidate Genes
Source: PLoS One. 2010 Dec 6;5(12):e14234. doi: 10.1371/journal.pone.0014234 (PMC2997792; doi:10.1371/journal.pone.0014234)
Supplement: Table S3 — The list of motifs identified in the putative promoter sequences investigated. Regulatory elements, identified by PLACE database in the consensus sequences of (a) AGP-like promoter, (b) PCBER promoter, (c) AEOMT promoter, (d) CCoAOMT promoter, (e) Chitinase promoter, (f) PR10 promoter. (0.11 MB DOC) [file pone.0014234.s006.doc]

**Table S3.** The list of motifs identified in the putative promoter sequences investigated. Regulatory elements, identified by PLACE database in the consensus sequences of (a) AGP-like promoter, (b) PCBER promoter, (c) AEOMT promoter, (d) CCoAOMT promoter, (e) Chitinase promoter, (f) PR10 promoter.

| **PLACE Signal Name** | **Freq.** | **Motif** | **Promoter(s)** | **Putative regulator** |
| --- | --- | --- | --- | --- |
| CACTFTPPCA1 | 52 | YACT | a,b,c,d,e,f | Mem1, light, sugar |
| ARR1AT | 51 | NGATT | a,b,c,d,e,f | Myb-like, ARR1 |
| CAATBOX1 | 47 | CAAT | a,b,c,d,e,f | TBP |
| DOFCOREZM | 40 | AAAG | a,b,c,d,e,f | DOF |
| GTGANTG10 | 34 | GTGA | a,b,c,d,e,f | bZIP |
| EBOXBNNAPA | 32 | CANNTG | a,b,c,d,e,f | CPRF |
| MYCCONSENSUSAT | 32 | CANNTG | a,b,c,d,e,f | MYC/MYB |
| WRKY71OS | 32 | TGAC | a,c,d,e,f | WRKY,MYB, Gibberralins |
| GATABOX | 27 | GATA | a,b,c,d,e,f | GATABP |
| GT1CONSENSUS | 27 | GRWAAW | a,b,c,d,e,f | Light,GT1,SA |
| ROOTMOTIFTAPOX1 | 23 | ATATT | a,b,c,d,e,f | Root specific |
| WBOXNTERF3 | 15 | TGACY | c,d,e,f | WRKY, ethylene |
| RYREPEATBNNAPA | 14 | CATGCA | a,b,c | ABI-B2 |
| IBOXCORE | 13 | GATAA | b,c,d,e,f | Light, CAB |
| ACGTATERD1 | 12 | ACGT | a,b,c,d | erd |
| WBOXATNPR1 | 12 | TTGAC | a,c,d,e,f | WRKY,SA |
| CCAATBOX1 | 11 | CCAAT | a,b,c,d | TBP |
| POLLEN1LELAT52 | 11 | AGAAA | a,b,c,d,e,f | late pollen specific |
| INRNTPSADB | 10 | YTCANTYY | a,b,c,e,f | TFIIB |
| TATABOX5 | 10 | TTATTT | a,c,d,e | TBP |
| POLASIG1 | 9 | AATAAA | a,c,d,e | Poly adenylation signal |
| TAAAGSTKST1 | 9 | TAAAG | b,c,d | DOF, K+ influx |
| RAV1AAT | 8 | CAACA | c,d,e,f | RAV1, AP2, B3,VP1 |
| ASF1MOTIFCAMV | 7 | TGACG | c,f | ASF1, auxin,SA |
| MYBPLANT | 7 | MACCWAMC | b,c,d,e,f | phenylpropanoids, MYB |
| MYBPZM | 7 | CCWACC | c,d | MYB |
| POLASIG3 | 7 | AATAAT | e,f |  |
| TBOXATGAPB | 6 | ACTTTG | a,c,d,e | Light |
| -300ELEMENT | 5 | TGHAAARK | b,c | TBP |
| CGACGOSAMY3 | 5 | CGACG | b,c,e,a | sugar |
| ELRECOREPCRP1 | 5 | TTGACC | d,e,f | EREs, WRKYs,SA, ethylene |
| GT1GMSCAM4 | 5 | GAAAAA | a,c,d,f | GT1, light, SA |
| MARTBOX | 5 | TTWTWTTWTT | c,e |  |
| MYBCORE | 5 | CNGTTR | c,d,f | MYB |
| MYBST1 | 5 | GGATA | b,d,e | MYB |
| REALPHALGLHCB21 | 5 | AACCAA | c,d | ethylene, ABA, light |
| SEF4MOTIFGM7S | 5 | RTTTTTR | a,c,e | SEF |
| ABRELATERD1 | 4 | ACGTG | c,d | erd-ethylene |
| GAREAT | 4 | TAACAAR | b,e,f | Gibberalins |
| LTRECOREATCOR15 | 4 | CCGAC | a,b,d | AP2/DREB |
| MYB1AT | 4 | WAACCA | c,d,e | MYB, bHLH,ABA, stress |
| NODCON2GM | 4 | CTCTT | c,d | Nitrogen |
| OSE2ROOTNODULE | 4 | CTCTT | c,d | Nitrogen, infection |
| -300CORE | 3 | TGTAAAG | b,c | TBP |
| CAREOSREP1 | 3 | CAACTC | a,d | GARE |
| IBOX | 3 | GATAAG | c,d,e | Light |
| MYCATERD1 | 3 | CATGTG | a,d,e | MYC/MYB |
| SEBFCONSSTPR10A | 3 | YTGTCWC | a,c,f | SEBF |
| SEF3MOTIFGM | 3 | AACCCA | c | SEFs |
| TATCCAOSAMY | 3 | TATCCA | d,e | Gibberalins, sugar |
| WBOXHVISO1 | 3 | TGACT | c,e | WRKY, sugar |
| -10PEHVPSBD | 2 | TATTCT | c,f | light |
| ACGTCBOX | 2 | GACGTC | a | bZIP |
| BOXCPSAS1 | 2 | CTCCCAC | c,e | Light, RE1 |
| CACGTGMOTIF | 2 | CACGTG | c | ERF, bZIP,myb,GBF,light |
| CARGATCONSENSUS | 2 | CCWWWWWWGG | c | FLC,cold, CONSTANS |
| CARGCW8GAT | 2 | CWWWWWWWWG | e | AGAMOUS |
| CATATGGMSAUR | 2 | CATATG | a | SAUR-NDE |
| CIACADIANLELHC | 2 | CAANNNNATC | a,f | CCA |
| DPBFCOREDCDC3 | 2 | ACACNNG | d,e | DPBFs, ABI,bZIP,GAI1,ABA |
| DRECRTCOREAT | 2 | RCCGAC | b,d | DRE/CRT, cold, dehydration |
| EMHVCHORD | 2 | TGTAAAGT | b | Endosperm motif |
| INTRONLOWER | 2 | TGCAGG | a,b | TFIIB |
| MYB2CONSENSUSAT | 2 | YAACKG | c | MYB |
| MYCATRD22 | 2 | CATGTG | d,e | MYC, ABA, dehydration |
| NTBBF1ARROLB | 2 | ACTTTA | b | DOF, Auxin |
| PREATPRODH | 2 | ACTCAT | b,e | Proline/ATB2 |
| PYRIMIDINEBOXOSRAMY1A | 2 | CCTTTT | d,f | BPBF,DOF, gibberalin |
| QELEMENTZMZM13 | 2 | AGGTCA | e,f | enhancer |
| S1FBOXSORPS1L21 | 2 | ATGGTA | b,c | S1F |
| T/GBOXATPIN2 | 2 | AACGTG | c,d | Jasmonate, Wounding,LAP, MYC |
| TGTCACACMCUCUMISIN | 2 | TGTCACA | c,f | Serine |
| WUSATAg | 2 | TTAATGG | c | WUS |
| 2SSEEDPROTBANAPA | 1 | CAAACAC | a | bZIP |
| AGCBOXNPGLB | 1 | AGCCGCC | e | ERF,erebp, ethylene, osmolarity,SA, sugar |
| AMYBOX1 | 1 | TAACARA | f | sugar |
| AT1BOX | 1 | AATATTTTTATT | c | CAB |
| BOXIINTPATPB | 1 | ATAGAA | c | Light |
| BS1EGCCR | 1 | AGCGGG | d | BS1, Cinnamoyl-CoA |
| CANBNNAPA | 1 | CNAACAC | a | Zinc finger |
| CEREGLUBOX2PSLEGA | 1 | TGAAAACT | c | dark |
| ERELEE4 | 1 | AWTTCAAA | a,e,f | ERE, ethylene |
| GCCCORE | 1 | GCCGCC | e | ERE,ERF,jasmonate,MYB |
| GT1CORE | 1 | GGTTAA | e | GT1 |
| IBOXCORENT | 1 | GATAAGR | c | Light |
| L1BOXATPDF1 | 1 | TAAATGYA | d | ATML, MYB |
| LEAFYATAG | 1 | CCAATGT | c | Leafy |
| LTRE1HVBLT49 | 1 | CCGAAA | a | AP2/DREB |
| MYB1LEPR | 1 | GTTAGTT | b | ERF/MYB |
| MYB26PS | 1 | GTTAGGTT | e | MYB, phenylpropanoids |
| MYB2AT | 1 | TAACTG | a | ABA, stress |
| MYBGAHV | 1 | TAACAAA | f | MYB, gibberalin, sugar |
| PALBOXAPC | 1 | CCGTCC | d | Phenylalanine |
| POLASIG2 | 1 | AATTAAA | e |  |
| PROLAMINBOXOSGLUB1 | 1 | TGCAAAG | e | sugar |
| RBCSCONSENSUS | 1 | AATCCAA | d | light |
| SP8BFIBSP8BIB | 1 | TACTATT | c | SPFs |
| SV40COREENHAN | 1 | GTGGWWHG | e | enhancer |
| TATABOX2 | 1 | TATAAAT | e | TBP |
| TATABOX3 | 1 | TATTAAT | c | TBP |
