# Supplementary material for: Evolution of Disease Response Genes in Loblolly Pine: Insights from Candidate Genes
Source: PLoS One. 2010 Dec 6;5(12):e14234. doi: 10.1371/journal.pone.0014234 (PMC2997792; doi:10.1371/journal.pone.0014234)
Supplement: Table S4 — Primer sequences for the amplification products used for the diversity screen from the target disease resistance loci, listed according to their locus acronyms and NCBI-GI numbers of the templates used for primer design. Chitinase promoter and PR10 promoter template sequences were kindly provided by Dr. John Davis, UFL-Gainesville. (0.06 MB DOC) [file pone.0014234.s007.doc]

**Table S4-** Primer sequences for the amplification products used for the diversity screen from the target disease resistance loci, listed according to their locus acronyms and NCBI-GI numbers of the templates used for primer design. Chitinase promoter and PR10 promoter template sequences were kindly provided by Dr. John Davis, UFL-Gainesville.

| **Locus Acronym** | **Template GI NCBI** | **PCR-Forward Sequence** | **PCR-Reverse Sequence** |
| --- | --- | --- | --- |
| *4cl* | 54634049 | TCTGGCTCCTGCGGAACAGT | AGGAACGACTGCTGCGTCAG |
| *axr* | 32396294 | tagcagacggtattcacacagtcc | ccacaaccaccttgcatcattattt |
| *bhlh62-like* | 8179111 | accctgtatcgatgggtatggagat | tttcatgtggtttgttggtacagaacctgcaatca |
| *c3h* | 37569495 | GTTCTGCAGGGGAATGTCTGTTC | ACCAGTGGGAAGCCAATGGA |
| *c4h2-f1r1* | 60665573 | TTTTCGCTGTAATTGTCGGTGC | CCCGTGGTAGACGCCTCG |
| *c4h2-f4r4* | 60662305 | ATGGGGACTCGCCGAAATC | TGAGCTTCAGGCTATTGGTTTGG |
| *caf1* | 57883552 | aactctgccaaagtcacaagaaaaaca | ggacaatagagacttaaatggaatccaaca |
| *dicer-like* | 18110555 | GGTACCCGGGAATTCGGCCATTATG | CCAACAGAGTAAAGATAAAGATTTGAGAATAAGTTGAGTC |
| *ccoaomt* | 58029096 | GCAGCAGAAGTGAAGGCTCAGA | TCTTTCCATCATCGGGCAATG |
| *cesa3* | 13126988 | GACGAAAGAGCATTGTTGATGAGC | AGTGAGCAAACTGACGGCTGG |
| *cesa7* | 67194164 | CCATACAGAGCTTGCCTGTTGAAAACGC | AGGTTCGAGTCTCTGCAGTTCTAACAAATGCC |
| *chi4-like* | 48933590 | TGAATCGCTTTTATTGAGTGCAACA | GGAGGAGATAGTCAAGAGCGCCTAC |
| *comt2.1* | 34505679 | CGAAATGGGATCGGCTTCC | GCAATCACTGCTTTAGGGTATTCCA |
| *comt2.2* | 57887938 | GATTCATCATGTTTTGGCTCCTGTAAC | CTCTTCTTGTTCAGCGAAAATTCCATA |
| *comt4* | 10682039 | ctaggttgcgcttggaccgt | tttgtgtggcgatttggcaa |
| *ein2/cop9-like* | 69435726 | gggatcttgttgagttgcactgttt | cgctgctcaagatataaagggcatt |
| *erd3* |  | CCCATGCGGTCTTGTATAGT | ATCCTGGGAAAATGAAACG |
| *erebp-like* | 57888159 | tgcaagctgtaaggtaaaaccctcat | caacatcaaaactgaaaccaccagtc |
| *erf1-like* | 34489945 | atgatacggagaccaatggcatag | tgagatgagaatgggaaggaatcag |
| *gatabp1* | 66742607 | CGACCTTTGTGGAGGATTCTTTTATG | CTTGGGCTTTCTTGCTATGGGTTTT |
| *gatabp2* | 37568709 | ctggagactgatgcaaacaataatgg | tttctttgcagggaagtaggattctg |
| *ldox-a* | 48943503 | aaattactatccmcgctgtccacaa | tacggttcaagtagactgttcgag |
| *ldox-c* | 48945314 | GTCCGATTAAGGCGTTTTTCGGGGGA | ATGCCAGTGGTCAGCTTGAATGGGAGGA |
| *mybs3-like* | 60361156 | ctgttgccaaacctctagattctgct | gtcttcagcagctccagattcagatt |
| *myb3-psd* | 13536808 | AATCGATAGATTTGCAACAAGGGATCT | TTCATAACATGCATCACCAAAAATGG |
| *nac1* | 48107215 | cttcggctgtggatgtattgga | atcgtttcaactgccttggctt |
| *cyp450-like* | 34489089 | aggttggaattgagcagatgtcttg | attatgcagtctgcttgttgctgtg |
| *paeomt* | 18476475 | aacaaaaggcgaggctcaaattaaa | gccaggccgttcatgttcgaatccatc |
| *pagp-like* | 607775 | TACAAGTTTGATAGGTAACCCCTGC | GGAATGTGGGAGTGGGACTG |
| *pal1* | 57882094 | TTCCTGTGTTTGAAGCCGAGC | TGATCTGCCACCCTTACATATTTCTG |
| *pccoaomt* | 34489245 | TCACCTAAAATCACAACCTCGCTG | CCACTTCTTGATGGCGGACAA |
| *pchi* |  | TCGTCAACTTGCTGCCACTAAAACT | CATACTCATCCTCAGCACCAGATGG |
| *pcna* | 34360002 | CTCTCAGAGTTTGTTTTCACTGTAACAATG | TCATTTCGAAGTCGGAAATCTTGTCCTGGT |
| *ppcber* | 49446002 | aattcaaacaaacgccagaaattga | atcctgttgcgccaattaggagtat |
| *ppr10* |  | AAGATGGTGTCAGGGACTGCAATAAC | TGCTTTCTTCAAGCTGAGCACCA |
| *pr4.1* | 34490105 | tagcgagagagtgcttacgcctaga | attatgcagtctgcttgttgctgtg |
| *pr4.3* | 48946209 | TAGCGAGAGAGTGCTTACGCCTAGA | CGCCTATAATTACGCCCTGGATGGCCTT |
| *enth1-like* | 49444078 | ggaatgaatccagcaatgcctatg | agtttgaaatggaagggacaaacaat |
| *set-like-b* | 12126662 | ttatttacatccaacagcgccattt | gaaagtatggattgccaacttgcac |
| *set-like-c* | 37561780 | ggaacagacatttgcttcaaaatcg | acgcaagaacccaattcttctcttc |
| *tps-like* | 18111379 | cgggggtctatacattactatgct | gccttgccaaagattgttatttta |
| *wrky-like-1* | 48936517 | gattgtaacatatgagggcgaacaca | tcacagaaaatgtacagccatgatgtagt |
| *wrky-like-2* | 18613377 | ctatatggagagtagacaccagtagcagtataa | aatgaaacaagatgtcatgagcatggttctt |
