# Supplementary material for: Evolution of Disease Response Genes in Loblolly Pine: Insights from Candidate Genes
Source: PLoS One. 2010 Dec 6;5(12):e14234. doi: 10.1371/journal.pone.0014234 (PMC2997792; doi:10.1371/journal.pone.0014234)
Supplement: Table S5 — Locus statistics for sequences from each loci. (0.31 MB DOC) [file pone.0014234.s008.doc]

**Table S5-** Locus statistics for sequences from each loci.

| **Locus**  **Name** | **Number of alleles**  **(N)** | **template**  **GI-NCBI** | **Functional**  **Class** | **Region** | **Sites** | **length aligned** | **θπ total** | **θπ Silent** | **θw Total** | **θw silent** | **K(JC)**  **Total** | **K(JC)**  **Silent** | **Sil**  **Sites** | **NS**  **Sites** | **Mut**  **(Tot)** | **Sil**  **Mut** | **NS**  **Mut** |
| --- | --- | --- | --- | --- | --- | --- | --- | --- | --- | --- | --- | --- | --- | --- | --- | --- | --- |
| *4cl* | 32 | 67561330 | enzyme | exon | 1-105 | 105 | 0.00484 | 0.02193 | 0.00236 | 0.01072 | 0.01382 | 0.06477 | 23.17 | 80.83 | 1 | 1 | 0 |
|  |  |  |  | Intron | 106-193 | 88 | 0.01711 | 0.01711 | 0.00847 | 0.00847 | 0.01543 | 0.01543 | 88 |  | 3 | 3 |  |
|  |  |  |  | exon | 194-340 | 147 | 0.00646 | 0.03015 | 0.00507 | 0.02365 | 0.00576 | 0.02728 | 31.5 | 115.5 | 3 | 3 | 0 |
|  |  |  |  | Intron | 341-433 | 90 | 0.00802 | 0.00802 | 0.00828 | 0.00828 | 0.00838 | 0.00838 | 90 |  | 3 | 3 |  |
|  |  |  |  | exon | 434-480 | 47 | 0.00669 | 0.02301 | 0.00528 | 0.01817 | 0.004 | 0.01385 | 13.67 | 32.33 | 1 | 1 | 0 |
|  |  |  |  | Total Coding | 1-480 | 299 | 0.00593 | 0.02593 | 0.00415 | 0.01817 | 0.0083 | 0.03703 | 68.33 | 228.67 | 5 | 5 | 0 |
|  |  |  |  | TOTAL | 1-480 | 477 | 0.00839 | 0.01624 | 0.00573 | 0.01109 | 0.00963 | 0.01875 | 246.33 | 228.67 | 11 | 11 | 0 |
| *axr* | 32 | 32396294 | regulatory | 5' Flanking | 1-130 | 130 | 0 | 0 | 0 | 0 | 0 | 0 | 130 |  | 0 | 0 |  |
|  |  |  | element | exon | 131-413 | 283 | 0.00043 | 0 | 0.00088 | 0 | 0.00376 | 0.0158 | 63.97 | 218.03 | 1 | 0 | 1 |
|  |  |  |  | TOTAL | 1-413 | 413 | 0.00029 | 0 | 0.0006 | 0 | 0.00258 | 0.00517 | 193.97 | 218.03 | 1 | 0 | 1 |
| *bhlh62-like* | 32 | 8179111 | regulatory | exon | 1-15 | 14 | 0.00446 | 0 | 0.01774 | 0 | 0.00224 | 0 | 3 | 11 | 1 | 0 | 1 |
|  |  |  | element | Intron | 15-193 | 179 | 0.00348 | 0.00348 | 0.00694 | 0.00694 | 0.00754 | 0.00754 | 179 |  | 5 | 5 |  |
|  |  |  |  | exon | 194-287 | 94 | 0 | 0 | 0 | 0 | 0 | 0 | 20.17 | 73.83 | 0 | 0 | 0 |
|  |  |  |  | Intron | 288-388 | 101 | 0.00062 | 0.00062 | 0.00246 | 0.00246 | 0.01028 | 0.01028 | 101 |  | 1 | 1 |  |
|  |  |  |  | exon | 389-398 | 10 | 0 | 0 | 0 | 0 | 0 | 0 | 3 | 6 | 0 | 0 | 0 |
|  |  |  |  | Total Coding | 1-398 | 118 | 0.00053 | 0 | 0.0021 | 0 | 0.00026 | 0 | 26.17 | 90.83 | 1 | 0 | 1 |
|  |  |  |  | TOTAL | 1-398 | 398 | 0.00188 | 0.00224 | 0.00437 | 0.00487 | 0.00607 | 0.0078 | 306.17 | 90.83 | 7 | 6 | 1 |
| *c3h* | 32 | 37569495 | enzyme | exon | 1-470 | 470 | 0.00052 | 0.00057 | 0.00158 | 0.00226 | NA | NA | 109.64 | 359.36 | 3 | 1 | 2 |
|  |  |  |  | Intron | 471-519 | 49 | 0.01296 | 0.01296 | 0.01014 | 0.01014 | NA | NA | 49 |  | 2 | 2 |  |
|  |  |  |  | exon | 520-814 | 295 | 0.00021 | 0.00096 | 0.00084 | 0.00381 | NA | NA | 65.17 | 228.83 | 1 | 1 | 0 |
|  |  |  |  | Intron | 815-1214 | 398 | 0.00316 | 0.00316 | 0.00873 | 0.00873 | NA | NA | 398 |  | 14 | 14 |  |
|  |  |  |  | exon | 1215-1282 | 68 | 0 | 0 | 0 | 0 | NA | NA | 15.67 | 52.33 | 0 | 0 | 0 |
|  |  |  |  | Total Coding | 1-1282 | 833 | 0.00037 | 0.00066 | 0.00119 | 0.00261 | NA | NA | 190.47 | 640.53 | 4 | 2 | 2 |
|  |  |  |  | TOTAL | 1-1282 | 1280 | 0.00172 | 0.00316 | 0.00388 | 0.00701 | NA | NA | 637.47 | 640.53 | 20 | 18 | 2 |
| *c4h2-*f1r1 | 29 | 60665573 | enzyme | exon | 1-456 | 456 | 0.00134 | 0 | 0.00223 | 0 | 0.00134 | NA | 107.68 | 342.32 | 4 | 2 | 2 |
| *c4h2-*f4r4 | 30 | 60662305 |  | exon | 1-542 | 540 | 0.004 | 0.0126 | 0.00321 | 0.01134 | 0.00401 | NA | 133.6 | 412.4 | 6 | 6 | 0 |
|  |  |  |  | TOTAL | 1-998 | 996 | 0.00264 | 0.0063 | ` | 0.00567 | 0.002675 | NA | 238.16 | 751.84 | 10 | 8 | 2 |
| *caf1* | 30 | 57883552 | regulatory | exon | 1-579 | 579 | 0.00123 | 0.00174 | 0.00174 | 0.00174 | NA | NA | 131.17 | 447.83 | 3 | 3 | 0 |
| *dicer-like* | 32 | 18110555 | regulatory | exon | 1-176 | 174 | 0.02249 | 0.00335 | 0.01416 | 0.00704 | NA | NA | 35.02 | 132.98 | 10 | 1 | 9 |
|  |  |  | element | 3'UTR | 177-250 | 69 | 0.00176 | 0.00176 | 0.00714 | 0.00714 | NA | NA | 69 | 0 | 2 | 2 | 0 |
|  |  |  |  | TOTAL | 1-250 | 243 | 0.0166 | 0.00229 | 0.01217 | 0.00711 | NA | NA | 104.02 | 132.98 | 12 | 3 | 9 |
| *ccoaomt* | 32 | 58029096 | enzyme | exon | 1-59 | 59 | 0.00789 | 0 | 0.00421 | 0 | 0.01121 | 0 | 15.75 | 42.25 | 1 | 0 | 1 |
|  |  |  |  | Intron | 60-173 | 114 | 0.02074 | 0.02074 | 0.01307 | 0.01307 | 0.0231 | 0.0231 | 114 |  | 6 | 6 |  |
|  |  |  |  | exon | 174-254 | 81 | 0.0115 | 0.05272 | 0.00613 | 0.02811 | 0.03555 | 0.07823 | 17.67 | 62.33 | 2 | 2 | 0 |
|  |  |  |  | Intron | 255-403 | 113 | 0.01686 | 0.01686 | 0.00879 | 0.00879 | 0.03713 | 0.03713 | 113 |  | 4 | 4 |  |
|  |  |  |  | exon | 404-503 | 100 | 0.00063 | 0.00254 | 0.00248 | 0.01007 | 0.00031 | 0.00127 | 24.65 | 74.35 | 1 | 1 | 0 |
|  |  |  |  | Total Coding | 1-503 | 240 | 0.00608 | 0.01712 | 0.00414 | 0.01283 | 0.01473 | 0.02351 | 58.06 | 178.94 | 4 | 3 | 1 |
|  |  |  |  | TOTAL | 1-503 | 467 | 0.01227 | 0.01847 | 0.00744 | 0.01132 | 0.02214 | 0.02872 | 285.06 | 178.94 | 14 | 13 | 1 |
| *cesa3* | 32 | 13126988 | enzyme | 5' Flanking | 1-320 | 275 | 0.00172 | 0.00172 | 0.00361 | 0.00361 | 0.00273 | 0.00273 | 275 |  | 4 | 4 |  |
|  |  |  |  | exon | 321-630 | 310 | 0 | 0 | 0 | 0 | 0 | 0 | 77.5 | 231.5 | 0 | 0 | 0 |
|  |  |  |  | TOTAL | 1-630 | 585 | 0.00081 | 0.00134 | 0.0017 | 0.00282 | 0.00128 | 0.00213 | 352.5 | 231.5 | 4 | 4 | 0 |
| *cesa7* | 30 | 8179660 | enzyme | exon | 1-200 | 199 | 0.00033 | 0.00153 | 0.00126 | 0.00578 | NA | NA | 43.67 | 154.33 | 1 | 1 | 0 |
|  |  | 67194164 |  | intron1 | 201-457 | 252 | 0.01125 | 0.01125 | 0.01603 | 0.01603 | NA | NA | 252 |  | 16 | 16 | 0 |
|  |  |  |  | exon | 458-541 | 84 | 0.00159 | 0.0062 | 0.00601 | 0.02348 | NA | NA | 21.5 | 62.5 | 2 | 2 | 0 |
|  |  |  |  | Total Coding | 1-541 | 284 | 0.0007 | 0.00307 | 0.00267 | 0.01162 | NA | NA | 65.17 | 216.83 | 3 | 3 | 0 |
|  |  |  |  | TOTAL | 1-541 | 536 | 0.00566 | 0.00957 | 0.00895 | 0.01512 | NA | NA | 317.17 | 216.83 | 19 | 19 | 0 |
| *chi4-like* | 32 | 48933590 | enzyme | exon | 1-91 | 91 | 0 | 0 | 0 | 0 | NA | NA | 22.67 | 67.33 | 0 | 0 | 0 |
|  |  |  |  | intron | 92-187 | 96 | 0 | 0 | 0 | 0 | NA | NA | 96 | 0 | 0 | 0 | 0 |
|  |  |  |  | exon | 188-484 | 297 | 0.00358 | 0.01042 | 0.00334 | 0.01037 | NA | NA | 71.81 | 222.19 | 4 | 3 | 1 |
|  |  |  |  | 3'UTR | 485-541 | 48 | 0 | 0 | 0 | 0 | NA | NA | 48 | 0 | 0 | 0 | 0 |
|  |  |  |  | Total Coding | 1-541 | 388 | 0.00274 | 0.00792 | 0.00256 | 0.00788 | NA | NA | 94.48 | 289.52 | 4 | 3 | 1 |
|  |  |  |  | TOTAL | 1-541 | 532 | 0.003757 | 0.019991 | 0.00351 | 0.01989 | NA | NA | 238.48 | 289.52 | 4 | 3 | 1 |
| *comt2.1* | 31 | 34505679 | enzyme | exon | 1-599 | 597 | 0.00423 | 0.00731 | 0.00378 | 0.01066 | NA | NA | 140.94 | 453.06 | 9 | 6 | 3 |
|  |  | 34505556 |  | 3' Flanking | 600-1201 | 601 | 0.00394 | 0.00394 | 0.00343 | 0.00343 | NA | NA | 601 | 0 | 8 | 8 |  |
|  |  |  |  | TOTAL | 1-1201 | 1198 | 0.0041 | 0.0046 | 0.00361 | 0.00484 | NA | NA | 723.94 | 453.06 | 17 | 14 | 3 |
| *comt2.2* | 28 | 57887938 | enzyme | 5' Flanking | 1-77 | 77 | 0 | 0 | 0 | 0 | 0 | 0 | 77 |  | 0 | 0 |  |
|  |  |  |  | exon | 78-383 | 271 | 0 | 0 | 0 | 0 | 0.19245 | 0.39816 | 69.59 | 197.41 | 0 | 0 | 0 |
|  |  |  |  | TOTAL | 1-383 | 383 | 0 | 0 | 0 | 0 | 0.19245 | 0.39816 | 146.59 | 197.41 | 0 | 0 | 0 |
| *comt4* | 32 | 10682039 | enzyme | 5' Flanking | 1-87 | 87 | 0.00139 | 0.00139 | 0.00285 | 0.00285 | 0.02409 | 0.02409 | 87 |  | 1 | 1 |  |
|  |  |  |  | exon | 88-444 | 357 | 0.00069 | 0.00293 | 0.00209 | 0.00887 | 0.01129 | 0.0492 | 84 | 273 | 3 | 3 | 0 |
|  |  |  |  | TOTAL | 1-444 | 444 | 0.00083 | 0.00215 | 0.00224 | 0.00581 | 0.01378 | 0.03632 | 171 | 273 | 4 | 4 | 0 |
| *ein2.1-like* | 31 | 17244421 | regulatory | 3'UTR | 1-505 | 505 | 0.0011 | 0.0011 | 0.00148 | 0.00148 | 0.00568 | 0.00568 | 504 | 0 | 3 | 0 | 0 |
| *(cop9)* |  | 69435726 | element | TOTAL | 1-505 | 505 | 0.0011 | 0.0011 | 0.00148 | 0.00148 | 0.00568 | 0.00568 | 504 | 0 | 3 | 3 | 0 |
| *erd3* | 32 | 51496786 | regulatory | exon | 1-322 | 322 | 0.00019 | 0 | 0.00077 | 0 | 0.01263 | 0.05175 | 79.99 | 241.01 | 1 | 0 | 1 |
|  |  |  | element | Intron | 323-411 | 89 | 0.0007 | 0.0007 | 0.00279 | 0.00279 | 0.01168 | 0.01168 | 89 |  | 1 | 1 |  |
|  |  |  |  | exon | 412-486 | 75 | 0 | 0 | 0 | 0 | 0.01345 | 0.05772 | 18 | 57 | 0 | 0 | 0 |
|  |  |  |  | Intron | 487-601 | 111 | 0.00113 | 0.00113 | 0.00447 | 0.00447 | 0.00963 | 0.00963 | 111 |  | 2 | 2 |  |
|  |  |  |  | exon | 602-826 | 225 | 0.00028 | 0 | 0.0011 | 0 | 0.00014 | 0 | 46 | 179 | 1 | 0 | 1 |
|  |  |  |  | 3' Flanking | 827-882 | 56 | 0.00112 | 0.00112 | 0.00443 | 0.00443 | 0.00056 | 0.00056 | 56 |  | 1 | 1 |  |
|  |  |  |  | Total Coding | 1-882 | 622 | 0.0002 | 0 | 0.0008 | 0 | 0.00818 | 0.03555 | 143.99 | 477.01 | 2 | 0 | 2 |
|  |  |  |  | TOTAL | 1-882 | 878 | 0.00043 | 0.00063 | 0.0017 | 0.00248 | 0.00823 | 0.01803 | 399.99 | 477.01 | 6 | 4 | 2 |
| *erebp-like* | 32 | 57888159 | regulatory | 5' Flanking | 1-85 | 84 | 0.00593 | 0.00593 | 0.00296 | 0.00296 | 0.00485 | 0.00485 | 84 |  | 1 | 1 |  |
|  |  |  | element | exon | 85-739 | 628 | 0.0092 | 0.01285 | 0.0083 | n.a. | 0.01798 | 0.03074 | 139.04 | 484.96 | 21 | 4 | 14 |
|  |  |  |  | 3' Flanking | 740-743 | 4 | 0 | 0 | 0 | 0 | 0 | 0 | 4 |  | 0 | 0 |  |
|  |  |  |  | TOTAL | 1-743 | 716 | 0.00877 | 0.01006 | 0.00763 | n.a. | 0.01633 | 0.02051 | 227.04 | 484.96 | 22 | 5 | 14 |
| *erf1-like* | 32 | 34489945 | regulatory | exon | 1-418 | 418 | 0.00625 | 0.01718 | 0.00356 | 0.01038 | NA | NA | 95.67 | 322.33 | 6 | 4 | 2 |
|  |  |  | element | exon | 419-480 | 59 | 0.0068 | 0.03167 | 0.00842 | 0.03921 | NA | NA | 12.67 | 46.33 | 2 | 2 | 0 |
|  |  |  |  | UTR | 481-628 | 141 | 0.00732 | 0.00732 | 0.00881 | 0.00881 | NA | NA | 141 | 0 | 5 | 5 | 0 |
|  |  |  |  | Total Coding | 1-628 | 477 | 0.00631 | 0.01887 | 0.00416 | 0.01375 | NA | NA | 108.33 | 368.77 | 8 | 6 | 2 |
|  |  |  |  | TOTAL | 1-628 | 628 | 0.00654 | 0.01234 | 0.00522 | 0.01095 | NA | NA | 249.33 | 368.77 | 13 | 11 | 2 |
| *gatabp1* | 32 | 13069880 | regulatory | 5' Flanking | 1-9 | 0 | 0 | 0 | 0 | 0 | 0 | 0 | 0 |  | 0 | 0 |  |
|  |  |  | element | exon | 9-323 | 287 | 0 | 0 | 0 | 0 | 0.08475 | 0.1342 | 67.14 | 214.86 | 0 | 0 | 0 |
|  |  |  |  | TOTAL | 1-323 | 287 | 0 | 0 | 0 | 0 | 0.08475 | 0.1342 | 67.14 | 214.86 | 0 | 0 | 0 |
| *gatabp2* | 32 | 37568709 | regulatory | intron | 1-499 | 484 | 0.00315 | 0.00315 | 0.00462 | 0.00462 | 0.00369 | 0.00369 | 484 |  | 8 | 8 |  |
|  |  |  | element | exon | 500-824 | 325 | 0.00151 | 0.00414 | 0.00153 | 0.00327 | 0.00589 | 0.02423 | 76.03 | 244.97 | 2 | 1 | 1 |
|  |  |  |  | TOTAL | 1-824 | 809 | 0.00249 | 0.00329 | 0.00338 | 0.00443 | 0.00457 | 0.00644 | 560.03 | 244.97 | 10 | 9 | 1 |
| *ldox-a* | 32 | 48943503 | enzyme | exon | 1-169 | 169 | 0.00598 | 0.02318 | 0.00592 | 0.01839 | 0.00767 | 0.03147 | 40.82 | 127.18 | 4 | 3 | 1 |
|  |  |  |  | Intron | 170-250 | 80 | 0.00984 | 0.00984 | 0.01252 | 0.01252 | 0.02127 | 0.02127 | 80 |  | 4 | 4 |  |
|  |  |  |  | exon | 251-464 | 214 | 0.00535 | 0.02552 | 0.00702 | 0.0335 | 0.00469 | 0.02264 | 44.83 | 168.17 | 6 | 6 | 0 |
|  |  |  |  | Total Coding | 1-464 | 383 | 0.00563 | 0.0244 | 0.00654 | 0.0263 | 0.006 | 0.02684 | 85.66 | 295.34 | 10 | 9 | 1 |
|  |  |  |  | TOTAL | 1-464 | 463 | 0.00635 | 0.01737 | 0.00757 | 0.01964 | 0.00862 | 0.02414 | 165.66 | 295.34 | 14 | 13 | 1 |
| *ldox-c* | 32 | 48945314 | enzyme | exon | 1-85 | 85 | 0.00481 | 0.01783 | 0.00876 | 0.03072 | 0.00258 | 0.00973 | 16.17 | 66.83 | 3 | 2 | 1 |
|  |  |  |  | Intron | 86-209 | 114 | 0.00451 | 0.00451 | 0.00653 | 0.00653 | 0.00247 | 0.00247 | 114 |  | 3 | 3 |  |
|  |  |  |  | exon | 210-537 | 328 | 0.0041 | 0.0122 | 0.00757 | 0.02159 | 0.01046 | 0.04109 | 80.5 | 247.5 | 10 | 7 | 3 |
|  |  |  |  | Intron | 538-617 | 80 | 0.00433 | 0.00433 | 0.00621 | 0.00621 | 0.01181 | 0.01181 | 80 |  | 2 | 2 |  |
|  |  |  |  | exon | 618-629 | 12 | 0.01008 | 0 | 0.02069 | 0 | 0.00523 | 0 | 1.04 | 7.96 | 1 | 0 | 1 |
|  |  |  |  | Total Coding | 1-629 | 425 | 0.00441 | 0.013 | 0.00818 | 0.02287 | 0.00873 | 0.03536 | 97.71 | 322.29 | 14 | 9 | 5 |
|  |  |  |  | TOTAL | 1-629 | 619 | 0.00442 | 0.00731 | 0.00762 | 0.01192 | 0.00797 | 0.01592 | 291.71 | 322.29 | 19 | 14 | 5 |
| *mybs3-like* | 32 | 60361156 | regulatory | 5' Flanking | 1-432 | 431 | 0.00029 | 0.00029 | 0.00115 | 0.00115 | 0.00831 | 0.00831 | 431 |  | 2 | 2 |  |
|  |  |  | element | exon | 433-593 | 161 | 0.00223 | 0.00855 | 0.00463 | 0.01433 | 0.00117 | 0.00452 | 34.66 | 124.34 | 3 | 2 | 1 |
|  |  |  |  | TOTAL | 1-593 | 592 | 0.00082 | 0.0009 | 0.0021 | 0.00213 | 0.00636 | 0.00803 | 465.66 | 124.34 | 5 | 4 | 1 |
| *myb3-psd* | 32 | 13536808 | regulatory | 5' Flanking | 1-15 | 15 | 0 | 0 | 0 | 0 | 0 | 0 | 15 |  | 0 | 0 |  |
|  |  |  | element | exon | 16-228 | 213 | 0.0018 | 0.00126 | 0.00588 | 0.00489 | 0.02174 | 0.02043 | 51.23 | 161.77 | 5 | 1 | 4 |
|  |  |  |  | 3' Flanking | 229-412 | 183 | 0.00212 | 0.00212 | 0.00821 | 0.00821 | 0.02783 | 0.02783 | 183 |  | 6 | 6 |  |
|  |  |  |  | TOTAL | 1-412 | 411 | 0.00187 | 0.00181 | 0.0067 | 0.00703 | 0.02364 | 0.0246 | 249.23 | 161.77 | 11 | 7 | 4 |
| *nac1* | 32 | 48107215 | regulatory | 3' Flanking | 1-726 | 726 | 0.00665 | 0.00665 | 0.00665 | 0.00665 | 0.02423 | 0.02423 | 726 |  | 19 | 19 |  |
|  |  |  | element | total | 1-726 | 726 | 0.00665 | 0.00665 | 0.00665 | 0.00665 | 0.02423 | 0.02423 | 726 |  | 19 | 19 |  |
| *cyp450-like* | 32 | 34489089 | enzyme | exon | 1-259 | 259 | 0.00047 | 0 | 0.00096 | 0 | 0.00801 | 0.01752 | 57.75 | 200.26 | 1 | 0 | 1 |
|  |  |  |  | 3' Flanking | 260-505 | 245 | 0.003 | 0.003 | 0.00912 | 0.00912 | 0.0139 | 0.0139 | 245 |  | 9 | 9 |  |
|  |  |  |  | TOTAL | 1-505 | 504 | 0.0017 | 0.00242 | 0.00493 | 0.00738 | 0.01087 | 0.01459 | 302.75 | 200.26 | 10 | 9 | 1 |
| *paeomt* | 32 | 18476475 | promoter | TOTAL | 1-975 | 975 | 0.00382 | 0.00382 | 0.00357 | 0.00357 | NA | NA | 975 | 0 | 14 | 14 | 0 |
| *pagp-like* | 31 | 607775 | promoter | TOTAL | 1-761 | 691 | 0.00905 | 0.00905 | 0.01413 | 0.01413 | NA | NA | 691 | 0 | 39 | 39 | 0 |
| *pal1* | 32 | 57882094 | enzyme | Coding Region | 1-246 | 246 | 0.00097 | 0.00289 | 0.00202 | 0.00409 | 0.02743 | 0.07072 | 60.66 | 185.34 | 2 | 1 | 1 |
|  |  |  |  | 3' Flanking | 247-394 | 148 | 0.00569 | 0.00569 | 0.00671 | 0.00671 | 0.02621 | 0.02621 | 148 |  | 4 | 4 |  |
|  |  |  |  | TOTAL | 1-394 | 394 | 0.00274 | 0.00488 | 0.00378 | 0.00595 | 0.02697 | 0.03888 | 208.66 | 185.34 | 6 | 5 | 1 |
| *pchi* | 32 | 49010353 | promoter | TOTAL | 1-634 | 558 | 0.01657 | 0.01657 | 0.0267 | 0.0267 | NA | NA | 558 | 0 | 60 | 60 | 0 |
| *pcna* | 32 | 34360002 | enzyme | 5' Flanking | 1-16 | 16 | 0.01096 | 0.01096 | 0.01552 | 0.01552 | 0.00588 | 0.00588 | 16 |  | 1 | 1 |  |
|  |  |  |  | exon | 17-335 | 319 | 0.00332 | 0.01519 | 0.00467 | 0.02139 | 0.00304 | 0.01404 | 69.67 | 249.33 | 6 | 6 | 0 |
|  |  |  |  | Intron | 336-452 | 113 | 0.01741 | 0.01741 | 0.01978 | 0.01978 | 0.01058 | 0.01058 | 113 |  | 9 | 9 |  |
|  |  |  |  | exon | 453-563 | 111 | 0.00203 | 0.01148 | 0.00224 | 0.01263 | 0.00113 | 0.00638 | 19.67 | 90.33 | 1 | 1 | 0 |
|  |  |  |  | Total Coding | 1-563 | 430 | 0.00299 | 0.01438 | 0.00404 | 0.01946 | 0.00255 | 0.01234 | 89.33 | 339.67 | 7 | 7 | 0 |
|  |  |  |  | TOTAL | 1-563 | 559 | 0.00613 | 0.0157 | 0.00755 | 0.01933 | 0.00426 | 0.01096 | 218.33 | 339.67 | 17 | 17 | 0 |
| *pccoaomt* | 24 | 34489245 | promoter | TOTAL | 1-741 | 738 | 0.00245 | 0.00245 | 0.00218 | 0.00218 | NA | NA | 738 | 0 | 6 | 6 | 0 |
| *ppcber* | 32 | 49446002 | promoter | TOTAL | 1-517 | 439 | 0.00419 | 0.00419 | 0.00798 | 0.00498 | NA | NA | 439 | 0 | 14 | 14 | 0 |
| *ppr10* |  | 37565538 | promoter | TOTAL | 1-227 | 224 | 0.006 | 0.006 | 0.00333 | 0.00333 | NA | NA | 224 | 0 | 3 | 3 | 0 |
| *pr4.1* | 32 | 34490105 | enzyme | exon | 1-230 | 230 | 0.00335 | 0.00845 | 0.00648 | n.a. | 0.04249 | 0.09348 | 55.11 | 174.89 | 6 | 3 | 3 |
|  |  |  |  | Intron | 231-316 | 86 | 0.00073 | 0.00073 | 0.00289 | 0.00289 | 0.03003 | 0.03003 | 86 |  | 1 | 1 |  |
|  |  |  |  | exon | 317-476 | 160 | 0.00076 | 0 | 0.00155 | 0 | 0.04882 | 0.14173 | 38.72 | 121.28 | 1 | 0 | 1 |
|  |  |  |  | 3' Flanking | 477-520 | 44 | 0.00284 | 0.00284 | 0.01129 | 0.01129 | 0.00142 | 0.00142 | 44 |  | 2 | 2 |  |
|  |  |  |  | Total Coding | 1-520 | 390 | 0.00228 | 0.00496 | 0.00446 | n.a. | 0.04508 | 0.11302 | 93.83 | 296.17 | 6 | 2 | 4 |
|  |  |  |  | TOTAL | 1-520 | 520 | 0.00207 | 0.00292 | 0.00478 |  | 0.03879 | 0.05773 | 223.83 | 296.17 | 9 | 6 | 4 |
| *pr4.3* | 31 | 48946209 | enzyme | exon | 1-134 | 134 | 0.01111 | 0.01367 | 0.00934 | 0.00804 | 0.0417 | 0.09521 | 31.14 | 100.86 | 5 | 1 | 4 |
|  |  |  |  | Intron | 135-220 | 86 | 0.02501 | 0.02501 | 0.01746 | 0.01746 | 0.0724 | 0.0724 | 86 |  | 6 | 6 |  |
|  |  |  |  | exon | 221-382 | 162 | 0.02143 | 0.08575 | 0.01545 | 0.05671 | 0.05119 | 0.15272 | 39.73 | 122.27 | 10 | 9 | 1 |
|  |  |  |  | 3' Flanking | 383-392 | 10 | 0 | 0 | 0 | 0 | 0 | 0 | 10 |  | 0 | 0 |  |
|  |  |  |  | Total Coding | 1-392 | 296 | 0.01675 | 0.05408 | 0.01268 | 0.03532 | 0.04688 | 0.12686 | 70.87 | 223.13 | 15 | 10 | 5 |
|  |  |  |  | TOTAL | 1-392 | 392 | 0.01814 | 0.03585 | 0.01341 | 0.024 | 0.05116 | 0.09037 | 166.87 | 223.13 | 21 | 16 | 5 |
| *enth1-like* | 32 | 49444078 | regulatory | exon | 1-72 | 71 | 0.00088 | 0 | 0.0035 | 0 | 0.00044 | 0 | 17.17 | 51.83 | 1 | 0 | 1 |
|  |  |  | element | 3' Flanking | 72-456 | 384 | 0.00452 | 0.00452 | 0.00648 | 0.0648 | 0.00557 | 0.00557 | 383 |  | 10 | 10 |  |
|  |  |  |  | TOTAL | 1-456 | 454 | 0.00395 | 0.00432 | 0.00602 | 0.0621 | 0.00621 | 0.00476 | 400.17 | 51.83 | 11 | 10 | 1 |
| *set-like-b* | 32 | 12126662 | regulatory | exon | 1-36 | 0 | 0 | 0 | 0 | 0 | 0 | 0 | 0 | 0 | 1 | 1 | 0 |
|  |  |  | element | Intron | 37-220 | 183 | 0.00367 | 0.00367 | 0.00196 | 0.00196 | 0.01066 | 0.01066 | 183 |  | 1 | 1 |  |
|  |  |  |  | exon | 221-325 | 105 | 0.00175 | 0 | 0.00473 | 0 | 0.00089 | 0 | 25.5 | 79.5 | 2 | 0 | 2 |
|  |  |  |  | 3' Flanking | 326-551 | 226 | 0.00055 | 0.00055 | 0.0022 | 0.0022 | 0.00472 | 0.00472 | 226 |  | 2 | 2 |  |
|  |  |  |  | Total Coding | 1-551 | 105 | 0.00175 | 0 | 0.00473 | 0 | 0.00089 | 0 | 25.5 | 79.5 | 2 | 0 | 2 |
|  |  |  |  | TOTAL | 1-551 | 458 | 0.00169 | 0.00156 | 0.00271 | 0.00197 | 0.00548 | 0.00638 | 378.5 | 79.5 | 6 | 4 | 2 |
| *set-like-c* | 32 | 37561780 | regulatory | exon | 1-426 | 426 | 0.00516 | 0.00764 | 0.01107 | 0.01324 | 0.031 | 0.04146 | 93.76 | 329.26 | 19 | 5 | 14 |
|  |  |  | element | TOTAL | 1-426 | 426 | 0.00516 | 0.00764 | 0.01107 | 0.01324 | 0.031 | 0.04146 | 93.76 | 329.26 | 19 | 5 | 14 |
| *tps-like* | 32 | 18111379 | enzyme | 5' Flanking | 1-240 | 239 | 0.00579 | 0.00579 | 0.00727 | 0.00727 | 0.01186 | 0.01186 | 239 |  | 7 | 7 |  |
|  |  |  |  | exon | 241-459 | 218 | 0.00393 | 0.00119 | 0.0057 | 0.00474 | 0.01258 | 0.03985 | 52.34 | 163.66 | 5 | 1 | 4 |
|  |  |  |  | TOTAL | 1-459 | 457 | 0.0049 | 0.00496 | 0.00652 | 0.00682 | 0.0122 | 0.01681 | 291.34 | 163.66 | 12 | 8 | 4 |
| *wrky-like-1* | 32 | 48936517 | regulatory | exon | 1-78 | 78 | 0 | 0 | 0 | 0 | 0.02609 | 0 | 18.29 | 59.71 | 0 | 0 | 0 |
|  |  |  | element | 3' Flanking | 79-304 | 223 | 0.00876 | 0.00876 | 0.00891 | 0.00891 | 0.01916 | 0.01916 | 223 |  | 8 | 8 |  |
|  |  |  |  | TOTAL | 1-304 | 301 | 0.00649 | 0.0081 | 0.0066 | 0.00823 | 0.02095 | 0.01769 | 241.29 | 59.71 | 8 | 8 | 0 |
| *wrky-like-2* | 32 | 18613377 | regulatory | 3'UTR | 1-453 | 453 | 0.0094 | 0.0094 | 0.01316 | 0.01316 | NA | NA | 453 | 0 | 24 | 24 | 0 |
|  |  |  |  | TOTAL | 1-453 | 453 | 0.0094 | 0.0094 | 0.01316 | 0.01316 | NA | NA | 453 | 0 | 24 | 24 | 0 |
