# Supplementary material for: Evolution of Disease Response Genes in Loblolly Pine: Insights from Candidate Genes
Source: PLoS One. 2010 Dec 6;5(12):e14234. doi: 10.1371/journal.pone.0014234 (PMC2997792; doi:10.1371/journal.pone.0014234)
Supplement: Table S6 — MKPRF input table (0.06 MB DOC) [file pone.0014234.s009.doc]

#### Table S6- MKPRF input table

| **CLASS**  **NAME** | **GENE NAME** | **FS*^a^*** | **SS*^b^*** | **FR*^c^*** | **SR*^d^*** | **N1*^e^*** | **N2*^f^*** | **LS*^g^*** | **LR*^h^*** | **H*^i^*** |
| --- | --- | --- | --- | --- | --- | --- | --- | --- | --- | --- |
| NZ^†^ | *4cl* | 2 | 11 | 0 | 0 | 32 | 1 | 246.33 | 228.67 | 1 |
| NZ | *cesa3* | 4 | 4 | 0 | 0 | 32 | 1 | 352.5 | 231.5 | 1 |
| NZ | *comt4* | 7 | 4 | 0 | 0 | 32 | 1 | 171 | 273 | 1 |
| NZ | *cyp450-like* | 0 | 9 | 0 | 1 | 32 | 1 | 302.75 | 200.26 | 1 |
| NZ | *pcna* | 1 | 17 | 0 | 0 | 32 | 1 | 218.33 | 339.67 | 1 |
| NZ | *enth1-like* | 7 | 10 | 0 | 1 | 32 | 1 | 400.17 | 51.83 | 1 |
| NZ | *sams2* | 0 | 6 | 0 | 0 | 32 | 1 | 259.67 | 254.33 | 1 |
| NZ | *lp5* | 1 | 13 | 0 | 6 | 32 | 1 | 130.76 | 267.24 | 1 |
| NZ | *ccoaomt* | 7 | 13 | 1 | 1 | 32 | 1 | 285.06 | 178.94 | 1 |
| NZ | *ldox-a* | 6 | 13 | 1 | 1 | 32 | 1 | 165.66 | 295.34 | 1 |
| NZ | *cpk3* | 6 | 7 | 1 | 1 | 32 | 1 | 312.83 | 245.17 | 1 |
| NZ | *erd3* | 5 | 4 | 2 | 2 | 32 | 1 | 399.99 | 477.01 | 1 |
| NZ | *pal1* | 5 | 5 | 2 | 1 | 32 | 1 | 208.66 | 185.34 | 1 |
| NZ | *rd21a* | 16 | 22 | 2 | 5 | 32 | 1 | 482.87 | 441.13 | 1 |
| NZ | *ldox-c* | 7 | 14 | 5 | 5 | 32 | 1 | 291.71 | 322.29 | 1 |
| NZ | *dhn1* | 11 | 5 | 7 | 2 | 32 | 1 | 224.85 | 407.15 | 1 |
| NZ | *pr4.1* | 7 | 6 | 8 | 4 | 32 | 1 | 223.83 | 296.17 | 1 |
| NZ | *dhn2* | 10 | 9 | 8 | 4 | 32 | 1 | 189.46 | 319.54 | 1 |
| R^‡^ | *gatabp1* | 29 | 0 | 0 | 0 | 32 | 1 | 67.14 | 214.86 | 1 |
| R | *nac1* | 12 | 19 | 0 | 0 | 32 | 1 | 726 | 0 | 1 |
| R | *set-like-b* | 10 | 3 | 0 | 2 | 32 | 1 | 378.5 | 79.5 | 1 |
| R | *wrky-like-1* | 2 | 8 | 0 | 0 | 32 | 1 | 241.29 | 59.71 | 1 |
| R | *bhlh62-like* | 2 | 6 | 1 | 1 | 32 | 1 | 306.17 | 90.83 | 1 |
| R | *gatabp2* | 13 | 10 | 1 | 1 | 32 | 1 | 560.03 | 244.97 | 1 |
| R | *mybs3-like* | 4 | 4 | 1 | 1 | 32 | 1 | 465.66 | 124.34 | 1 |
| R | *axr* | 8 | 0 | 5 | 1 | 32 | 1 | 193.97 | 218.03 | 1 |
| R | *erf1-like* | 5 | 2 | 6 | 0 | 32 | 1 | 51.7 | 179.3 | 1 |
| R | *set-like-c* | 3 | 5 | 9 | 14 | 32 | 1 | 93.76 | 329.26 | 1 |
| R | *myb3-psd* | 6 | 7 | 11 | 4 | 32 | 1 | 249.23 | 161.77 | 1 |
| R | *erebp-like* | 15 | 5 | 19 | 14 | 32 | 1 | 227.04 | 484.96 | 1 |

^†^ NZ: Metabolic Enzyme

^‡^ R : Regulators

*^a^* FS : # fixed silent silents

*^b^* SS : # segregating silent sites

*^c^*. FR : # fixed replacement sites

*^d^*. SR : # segregating replacement sites

*^e^*. N1 : number of sequences sampled in species 1.

*^f^*. N2 : number of sequences sampled in species 2.

*^g^*. LS : total number of silent sites in the alignment.

*^h^*. LR : total number of replacement sites in the alignment.

*^i^*. H: haploid ratio (1.0 for autosomal, 0.75 for X-linked, 0.25 for Y-linked).
