# Supplementary material for: Evolution of Disease Response Genes in Loblolly Pine: Insights from Candidate Genes
Source: PLoS One. 2010 Dec 6;5(12):e14234. doi: 10.1371/journal.pone.0014234 (PMC2997792; doi:10.1371/journal.pone.0014234)
Supplement: Table S7 — WH input table (0.05 MB DOC) [file pone.0014234.s010.doc]

#### Table S7-WH input table

| **Locus** | **Inheritance scalar*^a^*** | **L*^b^*** | **N_1_*^c^*** | **N_2_*^d^*** | **S_1_*^e^*** | **S_2_*^f^*** | **S_S_*^g^*** | **S_F_*^h^*** | **ρ_1_*^i^*** | **ρ_2_ *^j^*** |
| --- | --- | --- | --- | --- | --- | --- | --- | --- | --- | --- |
| *4cl* | 1 | 480 | 32 | 2 | 11 | 2 | 0 | 2 | 2.542 | -1 |
| *axr* | 1 | 413 | 32 | 2 | 1 | 2 | 0 | 13 | 0 | -1 |
| *bhlh62-like* | 1 | 398 | 32 | 2 | 7 | 0 | 0 | 3 | 45.23 | -1 |
| *ccoaomt* | 1 | 501 | 32 | 1 | 14 | 0 | 0 | 10 | 0 | -1 |
| *cesa3* | 1 | 630 | 32 | 2 | 4 | 0 | 0 | 4 | 11.01 | -1 |
| *enth1-like* | 1 | 456 | 32 | 2 | 11 | 0 | 0 | 7 | 5.58 | -1 |
| *erd3* | 1 | 882 | 32 | 1 | 8 | 0 | 0 | 22 | 2.042 | -1 |
| *erebp-like* | 1 | 743 | 32 | 2 | 22 | 1 | 0 | 24 | 0.503 | -1 |
| *erf1-like* | 1 | 306 | 32 | 1 | 6 | 0 | 0 | 14 | 4.02 | -1 |
| *gatabp1* | 1 | 323 | 32 | 2 | 0 | 0 | 0 | 15 | 0 | -1 |
| *gatabp2* | 1 | 824 | 32 | 2 | 11 | 3 | 0 | 12 | 3.518 | -1 |
| *ldox-a* | 1 | 465 | 32 | 2 | 14 | 3 | 0 | 7 | 0 | -1 |
| *ldox-c* | 1 | 630 | 31 | 1 | 18 | 0 | 0 | 12 | 5.528 | -1 |
| *lp5* | 1 | 500 | 32 | 2 | 20 | 2 | 4 | 1 | 0 | -1 |
| *mybs3-like* | 1 | 583 | 32 | 1 | 5 | 0 | 0 | 5 | 0 | -1 |
| *myb3-psd* | 1 | 412 | 31 | 2 | 11 | 1 | 0 | 17 | 0.701 | -1 |
| *nac1* | 1 | 726 | 31 | 2 | 19 | 1 | 0 | 12 | 1.05 | -1 |
| *cyp450-like* | 1 | 505 | 32 | 1 | 10 | 0 | 0 | 0 | 0 | -1 |
| *pal1* | 1 | 394 | 32 | 2 | 6 | 5 | 0 | 6 | 18.09 | -1 |
| *pcna* | 1 | 439 | 32 | 2 | 16 | 0 | 0 | 2 | 17.59 | -1 |
| *pr4.1* | 1 | 542 | 31 | 2 | 18 | 4 | 1 | 15 | 4.02 | -1 |
| *rd21a* | 1 | 1063 | 32 | 1 | 25 | 0 | 0 | 17 | 0 | -1 |
| *sams2* | 1 | 515 | 32 | 2 | 6 | 8 | 0 | 0 | 0 | -1 |
| *set-like-b* | 1 | 553 | 32 | 2 | 6 | 8 | 0 | 11 | 4.02 | -1 |
| *set-like-c* | 1 | 426 | 32 | 2 | 19 | 18 | 0 | 1 | 1.01 | -1 |
| *tps-like* | 1 | 460 | 32 | 2 | 12 | 3 | 0 | 11 | 1.01 | -1 |
| *wrky-like1* | 1 | 304 | 32 | 2 | 7 | 1 | 2 | 1 | 6.53 | -1 |

*^a^* inheritance scalar (1.0 autosomal; 0.75 X –linked; 0.25 mitochondrial or Y-linked)

*^b^* length in base pairs

*^c^* number of sequences for species 1

*^d^* number of sequences for species 2

*^e^* polymorphisms exclusive to species 1

*^f^* polymorphisms exclusive to species 2

*^g^* shared polymorphisms

*^h^* fixed differences

*^i^* recombination rate in S1 estimated by LDhat according to Hudson 2001

*^j^* recombination rate in S2 is unknown, therefore substituted with -1, according to the software’s instructions.
