# Supplementary material for: Evolution of Disease Response Genes in Loblolly Pine: Insights from Candidate Genes
Source: PLoS One. 2010 Dec 6;5(12):e14234. doi: 10.1371/journal.pone.0014234 (PMC2997792; doi:10.1371/journal.pone.0014234)
Supplement: Table S8 — Using the best fit demographic model, common summary statistics of the observed site frequency spectrum are compared with neutral expectations under the model. The quantiles column shows the proportion of simulated values generated under the model which were less than the observed values. Quantiles less than 0.025 or greater than 0.975 indicate significantly low or high values of the respective summary statistics. (0.13 MB DOC) [file pone.0014234.s011.doc]

**Table S8.** Using the best fit demographic model, common summary statistics of the observed site frequency spectrum are compared with neutral expectations under the model. The quantiles column shows the proportion of simulated values generated under the model which were less than the observed values. Quantiles less than 0.025 or greater than 0.975 indicate significantly low or high values of the respective summary statistics.

| **Locus** | **Category** | **N^c^** | **Length** | **summary statistics^a^** | | | | **quantiles^b^** | | | |  |  |
| --- | --- | --- | --- | --- | --- | --- | --- | --- | --- | --- | --- | --- | --- |
|  |  |  |  | **S^d^** | **π^e^** | **D_T_^f^** | **D_FL_^g^** | **S** | **π** | **D_T_** | **D_FL_** | **Comments** |  |
| *aquamip* | DR | 32 | 611 | 5 | 1.05 | -0.44 | 0.17 | 0.063 | 0.183 | 0.476 | 0.714 |  | |
| *cpk3* | DR | 32 | 630 | 8 | 2.23 | 0.34 | 0.48 | 0.230 | 0.470 | 0.718 | 0.853 |  | |
| *dhn1* | DR | 31 | 643 | 12 | 2.63 | -0.44 | -0.01 | 0.493 | 0.518 | 0.458 | 0.614 |  | |
| *dhn2* | DR | 32 | 531 | 14 | 4.02 | 0.47 | 0.73 | 0.730 | 0.790 | 0.747 | 0.929 |  | |
| *erd3* | DR | 32 | 882 | 6 | 0.38 | -2.10 | -2.83 | 0.023 | 0.004 | 0.010 | 0.001 | low diversity, excess rare alleles | |
| *ferritin* | DR | 32 | 605 | 7 | 0.76 | -1.65 | -1.80 | 0.181 | 0.106 | 0.076 | 0.055 |  | |
| *lp31* | DR | 32 | 365 | 16 | 2.60 | -1.19 | -0.28 | 0.938 | 0.764 | 0.195 | 0.476 |  | |
| *lp33* | DR | 32 | 468 | 3 | 0.46 | -0.92 | -0.28 | 0.036 | 0.080 | 0.299 | 0.493 |  | |
| *lp5* | DR | 32 | 496 | 21 | 4.94 | -0.23 | 0.85 | 0.937 | 0.879 | 0.539 | 0.951 |  | |
| *mt-like* | DR | 32 | 403 | 9 | 2.05 | -0.28 | -0.35 | 0.607 | 0.629 | 0.516 | 0.439 |  | |
| *pp2c* | DR | 32 | 638 | 1 | 0.06 | -1.14 | -1.75 | 0.000 | 0.000 | 0.230 | 0.064 | low diversity | |
| *ppAP12* | DR | 32 | 378 | 10 | 3.05 | 0.71 | -0.62 | 0.716 | 0.809 | 0.800 | 0.351 |  | |
| *rd21a* | DR | 32 | 1000 | 26 | 7.13 | 0.33 | 0.07 | 0.742 | 0.772 | 0.721 | 0.698 |  | |
| *sams2* | DR | 32 | 461 | 3 | 1.01 | 0.79 | 0.80 | 0.038 | 0.280 | 0.819 | 0.932 |  | |
| *sodchl* | DR | 32 | 692 | 19 | 5.35 | 0.41 | 0.15 | 0.764 | 0.800 | 0.736 | 0.719 |  | |
| *ug2498* | DR | 32 | 310 | 9 | 1.45 | -1.09 | -1.67 | 0.760 | 0.593 | 0.230 | 0.106 |  | |
| *4cl* | DS | 32 | 480 | 11 | 4.00 | 1.44 | 0.63 | 0.634 | 0.824 | 0.928 | 0.889 |  | |
| *axr* | DS | 32 | 413 | 1 | 0.12 | -0.79 | 0.57 | 0.003 | 0.013 | 0.339 | 0.850 | low diversity | |
| *bhlh62-like* | DS | 32 | 398 | 7 | 0.75 | -1.67 | -1.80 | 0.437 | 0.236 | 0.060 | 0.059 |  | |
| *c3hf1ar1b* | DS | 32 | 552 | 5 | 0.88 | -0.79 | -0.56 | 0.092 | 0.168 | 0.349 | 0.377 |  | |
| *c3hf4r6* | DS | 32 | 829 | 16 | 1.38 | -2.20 | -2.38 | 0.531 | 0.160 | 0.005 | 0.010 | excess rare alleles | |
| *c4h1a* | DS | 32 | 1395 | 36 | 7.82 | -0.50 | -0.02 | 0.744 | 0.672 | 0.478 | 0.666 |  | |
| *c4h1b* | DS | 32 | 437 | 3 | 1.41 | 2.03 | 0.80 | 0.047 | 0.433 | 0.978 | 0.927 | excess mid frequency alleles | |
| *caf1* | DS | 30 | 579 | 3 | 0.70 | -0.21 | 0.81 | 0.015 | 0.098 | 0.544 | 0.951 | low diversity | |
| *ccoaoemt* | DS | 32 | 501 | 13 | 5.65 | 2.38 | 0.70 | 0.717 | 0.912 | 0.992 | 0.912 | excess mid frequency alleles | |
| *cesA3* | DS | 32 | 630 | 4 | 0.47 | -1.34 | -0.88 | 0.026 | 0.034 | 0.163 | 0.260 |  | |
| *cesA7a* | DS | 30 | 541 | 18 | 3.01 | -1.19 | -1.51 | 0.858 | 0.663 | 0.202 | 0.109 |  | |
| *chi4-like* | DS | 32 | 541 | 4 | 1.43 | 1.06 | 0.86 | 0.049 | 0.347 | 0.874 | 0.959 |  | |
| *comt2.1* | DS | 31 | 1201 | 16 | 4.80 | 0.65 | -0.53 | 0.249 | 0.503 | 0.801 | 0.398 |  | |
| *comt4* | DS | 32 | 444 | 4 | 0.37 | -1.60 | -0.88 | 0.099 | 0.065 | 0.079 | 0.254 |  | |
| *dicer-like* | DS | 34 | 315 | 16 | 4.40 | 0.39 | -0.30 | 0.948 | 0.932 | 0.737 | 0.474 |  | |
| *ein2-like* | DS | 32 | 505 | 3 | 0.55 | -0.62 | -0.28 | 0.027 | 0.094 | 0.405 | 0.497 |  | |
| *enth1-like* | DS | 32 | 456 | 11 | 1.79 | -1.11 | -1.21 | 0.666 | 0.518 | 0.231 | 0.185 |  | |
| *erebp-like* | DS | 32 | 743 | 22 | 6.28 | 0.46 | -0.11 | 0.808 | 0.832 | 0.750 | 0.593 |  | |
| *erf1-like* | DS | 32 | 306 | 6 | 2.61 | 2.06 | 0.92 | 0.501 | 0.825 | 0.979 | 0.952 | excess mid frequency alleles | |
| *erf1b* | DS | 32 | 628 | 13 | 4.04 | 0.78 | 0.38 | 0.573 | 0.729 | 0.821 | 0.819 |  | |
| *gatabp1* | DS | 32 | 323 | 1 | 0.06 | -1.14 | -1.75 | 0.009 | 0.009 | 0.221 | 0.065 | low diversity | |
| *gatabp2* | DS | 32 | 824 | 10 | 2.01 | -0.63 | 0.19 | 0.200 | 0.304 | 0.419 | 0.746 |  | |
| *ldoxa* | DS | 31 | 465 | 14 | 2.94 | -0.57 | 0.14 | 0.807 | 0.716 | 0.404 | 0.680 |  | |
| *ldoxc* | DS | 31 | 630 | 19 | 2.93 | -1.36 | 0.16 | 0.821 | 0.578 | 0.144 | 0.713 |  | |
| *mybs3-like* | DS | 32 | 583 | 5 | 0.48 | -1.65 | -1.29 | 0.076 | 0.049 | 0.075 | 0.141 |  | |
| *myb3-psd* | DS | 31 | 412 | 11 | 0.77 | -2.31 | -2.67 | 0.729 | 0.218 | 0.000 | 0.004 | excess rare alleles | |
| *nac1* | DS | 31 | 726 | 19 | 4.83 | 0.00 | 0.16 | 0.743 | 0.741 | 0.612 | 0.723 |  | |
| *cyp450-like* | DS | 32 | 505 | 10 | 0.85 | -2.06 | -1.82 | 0.533 | 0.190 | 0.008 | 0.053 | excess rare alleles | |
| *paeomt* | DS | 32 | 975 | 14 | 3.72 | 0.20 | -1.05 | 0.007 | 0.163 | 0.693 | 0.205 | low diversity | |
| *pagp1519* | DS | 31 | 782 | 47 | 6.99 | -1.53 | -1.06 | 0.812 | 0.529 | 0.108 | 0.182 |  | |
| *pal1* | DS | 32 | 394 | 6 | 1.08 | -0.80 | -0.33 | 0.341 | 0.369 | 0.335 | 0.452 |  | |
| *pchi* | DS | 32 | 634 | 58 | 9.41 | -1.32 | -0.75 | 0.957 | 0.768 | 0.186 | 0.316 |  | |
| *pcna* | DS | 32 | 439 | 16 | 3.20 | -0.69 | -0.02 | 0.886 | 0.774 | 0.377 | 0.585 |  | |
| *ppcber* | DS | 32 | 557 | 15 | 2.24 | -1.35 | -1.48 | 0.227 | 0.194 | 0.173 | 0.094 |  | |
| *ppr10* | DS | 32 | 227 | 4 | 1.41 | 1.02 | -0.01 | 0.077 | 0.392 | 0.865 | 0.592 |  | |
| *pr4.1* | DS | 31 | 542 | 18 | 1.69 | -2.16 | -1.76 | 0.863 | 0.395 | 0.003 | 0.061 | excess rare alleles | |
| *pr4.3* | DS | 31 | 392 | 21 | 7.11 | 1.16 | 0.65 | 0.981 | 0.985 | 0.889 | 0.877 | high diversity | |
| *set-like-b* | DS | 32 | 553 | 6 | 0.90 | -1.14 | -0.95 | 0.153 | 0.171 | 0.227 | 0.235 |  | |
| *set-like-c* | DS | 32 | 426 | 19 | 2.20 | -1.85 | -1.64 | 0.947 | 0.635 | 0.030 | 0.095 |  | |
| *tps-like* | DS | 32 | 460 | 12 | 2.24 | -0.82 | -1.38 | 0.717 | 0.609 | 0.333 | 0.126 |  | |
| *wrky-like-1* | DS | 32 | 304 | 8 | 1.95 | -0.09 | -0.01 | 0.699 | 0.722 | 0.565 | 0.551 |  | |
| *wrky-like-2* | DS | 32 | 453 | 24 | 4.26 | -1.03 | -1.27 | 0.978 | 0.861 | 0.264 | 0.169 | high diversity | |
| *agp4* | W | 32 | 367 | 24 | 6.31 | 0.17 | -0.37 | 0.993 | 0.977 | 0.657 | 0.425 | high diversity | |
| *agp6* | W | 32 | 498 | 14 | 2.59 | -0.87 | -1.05 | 0.765 | 0.639 | 0.317 | 0.200 |  | |
| *agplike* | W | 32 | 459 | 6 | 1.04 | -0.88 | -0.95 | 0.250 | 0.290 | 0.310 | 0.230 |  | |
| *cad* | W | 28 | 440 | 6 | 2.65 | 2.08 | 0.32 | 0.319 | 0.701 | 0.979 | 0.744 | excess mid frequency alleles | |
| *ccr1A* | W | 29 | 432 | 10 | 2.53 | -0.06 | -0.19 | 0.657 | 0.692 | 0.599 | 0.534 |  | |
| *ccr1b* | W | 32 | 434 | 9 | 1.82 | -0.58 | -1.23 | 0.558 | 0.547 | 0.415 | 0.186 |  | |
| *gly-hmt* | W | 32 | 552 | 13 | 3.47 | 0.19 | -0.25 | 0.659 | 0.718 | 0.674 | 0.517 |  | |
| *lims1* | W | 32 | 425 | 3 | 0.25 | -1.55 | -1.37 | 0.052 | 0.043 | 0.088 | 0.126 |  | |
| *lims2* | W | 32 | 449 | 5 | 0.43 | -1.77 | -2.01 | 0.171 | 0.080 | 0.040 | 0.039 |  | |
| *sams1* | W | 32 | 739 | 12 | 1.61 | -1.50 | -1.04 | 0.397 | 0.261 | 0.120 | 0.204 |  | |
| *tubulin* | W | 32 | 792 | 20 | 2.71 | -1.59 | 0.41 | 0.717 | 0.452 | 0.096 | 0.850 |  | |

^a^ Observed values

^b^ Quantile of observed value in simulated empirical distribution

^c^ number of chromosomes

^d^ number of segregating sites

^e^ average pairwise difference between haplotypes

^f^ Tajima's D

^g^ Fu and Li
